# Antioxidation of 2-phenylbenzofuran derivatives: structural-electronic effects and mechanisms†

**DOI:** 10.1039/c9ra10835a

**Published:** 2020-02-11

**Authors:** Phan Thi Thuy, Nguyen Van Trang, Ninh The Son

**Affiliations:** School of Natural Sciences Education, Vinh University Vietnam; Institute for Tropical Technology, Vietnam Academy of Science and Technology (VAST) 18 Hoang Quoc Viet, Caugiay Hanoi Vietnam nguyenvantrangsphn1909@gmail.com +84-985-538-722; Graduate University of Science and Technology, Vietnam Academy of Science and Technology (VAST) 18 Hoang Quoc Viet, Caugiay Hanoi Vietnam; Institute of Chemistry, Vietnam Academy of Science and Technology (VAST) 18 Hoang Quoc Viet, Caugiay Hanoi Vietnam yamantson@gmail.com +84-968-929-304

## Abstract

Stilbenoid-type 2-phenylbenzofuran derivatives, which are widely distributed in nature, are now promising antioxidant agents. In the present study, a quantum computational approach principally based on the DFT/B3LYP method with the 6-311++G(d,p) basis set was used to shed light on free radical scavenging for the isolated compounds stemofurans A-K and S-W. On the basis of the findings and from a thermodynamic perspective, the antioxidant activity of all studied compounds in the gaseous phase was mostly controlled by the O–H bond dissociation enthalpy (BDE), consistent with the hydrogen atom transfer (HAT) mechanism. The solvent effect was investigated, and the hydroxyl radicals of these studied compounds possessed the lowest proton affinity (PA) enthalpy and the sequential proton loss electron transfer (SPLET) pathway occurred in water, methanol and acetone. The studied compounds interacted with DPPH radicals, which is kinetic evidence of the involvement of two intermediates and one transition state. From both thermodynamics and kinetics perspectives, it can be proposed that stemofuran U is likely to be a leader compound in antioxidant drug development due to the presence of a 4′-OH moiety. Regarding the structure–bioactivity relationship, methylation can lead to a decrease in BDE.

## Abstract 2

The plausible antioxidative mechanism of 2-phenylbenzofurans based on DFT calculation.

## Graphical abstract

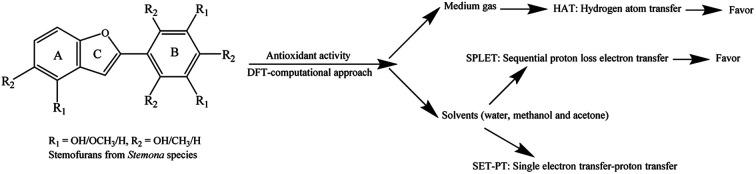

## 1. Introduction

Hydroxyl (˙OH), superoxide (O_2_^−^˙), alkoxyl (RO˙) or peroxyl (ROO˙) are types of reactive oxygen radicals. During conditional stress, *e.g.*, UV irradiation, these agents cause formation of reactive oxygen species (ROS).^1^ They can be present in either living organisms or outside environments. The overabundance of these chemical radicals results in a dramatic increase in ROS levels, which results in significant damage to cell structures and is the main cause of various diseases.^2^

Natural products, especially compounds isolated from plants, have historically proven their positive values in terms of therapeutic potency, whereas the employment of synthetic compounds is accompanied by high costs, a long duration of treatment, adverse effects and limitations in drug efficacy.^3^ Thus, scientific approaches seeking new drug lead compounds are always expected.

**Fig. 1:**
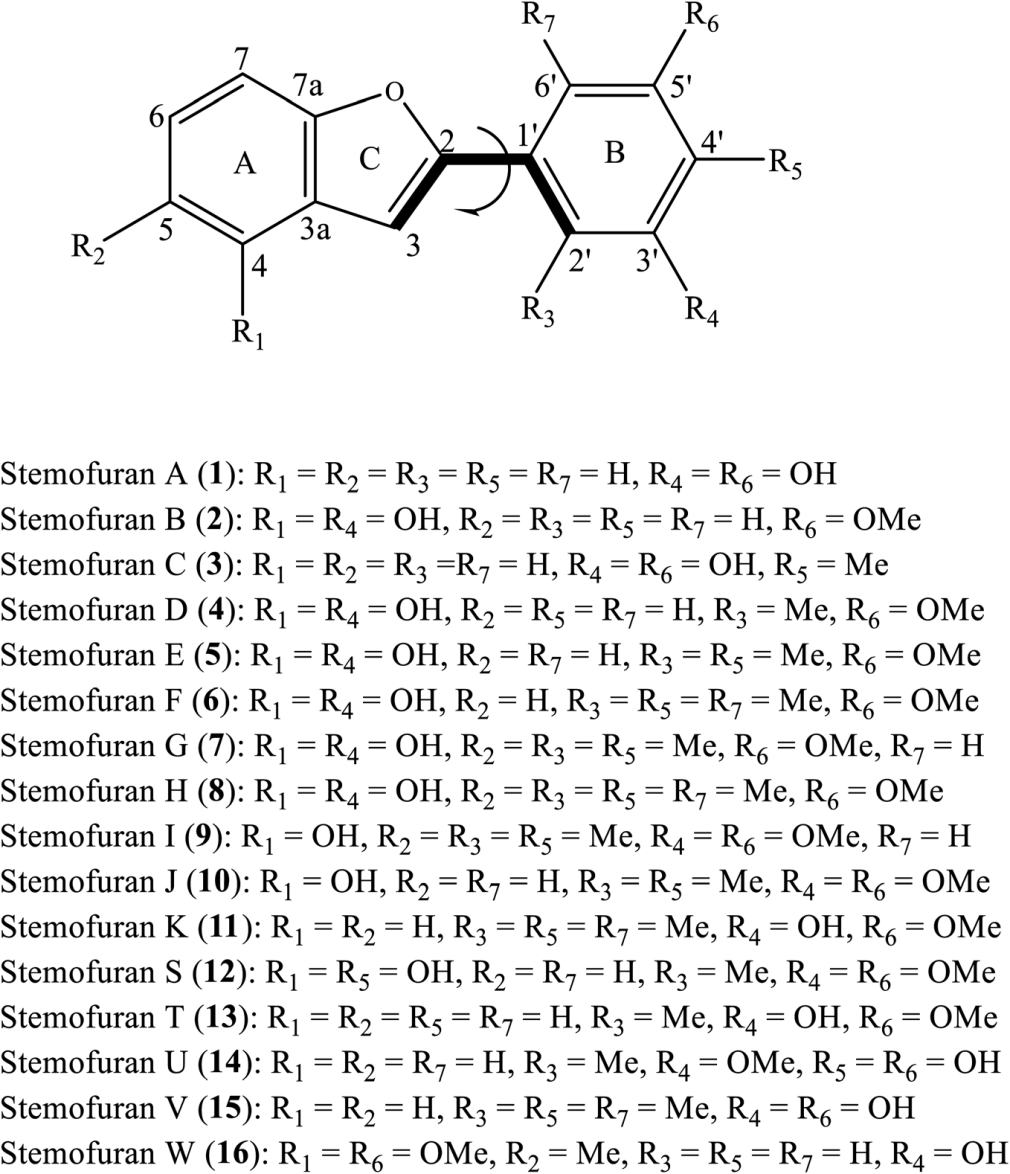
General structure of 2-phenylbenzofurans 1–16 with atom numbering.

Naturally occurring 2-phenylbenzofuran derivative compounds have been categorized as stilbenoids, which are composed of a benzofuran nucleus (systematic rings A–C) and a phenyl unit (ring B) substituted at carbon 2 (Fig. 1).^4–6^ 2-Phenylbenzofurans are now available in nature and have been found to indicate various pharmacological activities, but they are mostly applied to radical scavenging examinations.^7^ As examples, artopithecins A–D, four new prenylated 2-phenylbenzofurans derived from *Artocarpus pithecogallus* twigs, showed significant inhibition of mushroom tyrosinase.^5^ Two new analogues, trivially named regiafurans A-B, were promising candidates as antioxidants due to their remarkable IC_50_ values of 1.9–2.4 μg mL^−1^, compared with that of the positive control trolox (1.1 μg mL^−1^) in DPPH free radical scavenging assay.^7^

The plant extracts of *Stemona* species were reported to contain previously undescribed 2-phenylbenzofuran-type natural products. In a phytochemical investigation conducted by Pacher *et al.* (2002), the serial undescribed antifungal 2-phenylbenzofurans stemofurans A-K (1–11) were successfully precipitated from the methanolic extract of *Stemona collinsiae* roots.^4^ Furthermore, five other members, stemofurans S (12), T-V (13–15), and W (16), were chemical constituents of *S. collinsiae*, *S. burkillii*, and *S. lucida* species, respectively.^6^ With methylation occurring on rings A and/or B, stilbenoid-type methylated 2-phenylbenzofurans are characteristic of the medicinal plant *Stemona*. However, extensive antioxidant experimental assays for these compounds have not yet been performed. To the best of our knowledge, there has been no DFT-theoretical literature on the class of 2-phenylbenzofurans. Therefore, we herein set out to investigate the isolated compounds stemofurans A-K and S-W (1–11 and 12–16) *via* a computational DFT approach, principally based on the B3LYP functional with a 6-311++G(d,p) basis set, to gain insights into the structural features, conformations and electronic properties of these compounds in four media, namely, gas, water, methanol, and acetone. The results focused on explaining their reactivity with free radicals. It is expected that our results will be useful for the use of phenolic compound-type methylated phenylbenzofuran derivatives for antioxidant treatments.

## 2. Theoretical methodology

All calculations were carried out by means of the Gaussian 09 software package.^8^ To obtain the optimized structures, the B3LYP exchange-correlation functional method without constraints was used, coupled with the 6-311++G(d,p) basis set in the gaseous phase (dielectric constant, *ε* = 1.00) and in the solvents water (*ε* = 78.35), methanol (*ε* = 32.61), and acetone (20.70).^9–11^ Vibrational frequencies were calculated at the same level of theory to correct for zero-point energy (ZPE) and confirmed the presence of ground states lacking imaginary frequencies. The integral equation formalism polarizable continuum model (IEF-PCM) has been employed for estimating solvent effects.^14,15^

As mentioned in the literature, there are three known mechanisms, HAT (H atom transfer), SET-PT (single electron transfer-proton transfer), and SPLET (sequential proton loss electron transfer), describing the radical scavenging properties of the parent molecule (R–OH).^10–13^

(1) The HAT mechanism (eqn (1)) is involved in O–H bond breaking of R–OH and subsequent transfer to radicals and is often controlled by the homolytic bond dissociation enthalpy (BDE) (eqn (2)).

1 R–OH + R_1_O˙ → R–O˙ + R_1_OH

2 BDE = H(R–O˙) + H(H˙) − H(R–OH)

H(R–O˙), H(H˙), and H(R–OH) are the enthalpies of R–O˙, hydrogen radical atom, and the parent 2-phenylbenzofuran molecule, respectively.

(2) The SET-PT pathway involves two steps (eqn (3)). In detail, the first step involves loss of an electron to form the molecular radical cation R–OH^+^˙. After that, R–OH^+^˙ is deprotonated. The first reaction is characterized by the sum of the ionization potential (IP), whereas deprotonation is characterized by heterolytic bond dissociation enthalpy (PDE) (eqn (4) and (5)).

3 R–OH + R_1_˙ → R–OH˙^+^ + R_1_^−^ → R–O˙ + R_1_OH

4 IP = H (R–OH^+^˙) + H(e^−^) − H(R–OH)

5 PDE = H(R–O˙) + H(H^+^) − H(R–OH˙^+^)

H (R–OH^+^˙) presents the enthalpies of the 2-phenylbenzofuran radical cation R–OH^+^˙ after electron abstraction of the original 2-phenylbenzofuran molecule. The calculated gaseous-phase enthalpy values of H(e^−^) and H(H^+^) are 0.75 kcal mol^−1^ and 1.48 kcal mol^−1^, respectively, while the IEF-PCM model gave values of −25.08 kcal mol^−1^ and −244.15 kcal mol^−1^ in water, −20.54 kcal mol^−1^ and −247.97 kcal mol^−1^ in methanol and −28.43 kcal mol^−1^ and −255.61 kcal mol^−1^ in acetone, respectively.^16–18^

(3) In the third mechanism, SPLET, 2-phenylbenzofuran is deprotonated to afford a typical anion R–O^−^, and subsequent electron transfer from this anion occurs (eqn (6)). Proton affinity (PA) and the electron transfer enthalpy (ETE) are two conceptual parameters that correspond to deprotonation and electron transfer, respectively (eqn (7) and (8)).

6 R–OH → R–O^−^ + H^+^; R–O^−^ + R_1_˙ → R–O˙ + R_1_^−^; R_1_^−^ + H^+^ → R_1_H

7 PA = H(R–O^−^) + H(H^+^) − H(R–OH)

8 ETE = H(R–O˙) + H(e^−^) − H(R–O^−^)

H(R–O^−^) is the enthalpy of the 2-phenylbenzofuran anion after proton abstraction of the original molecule.

Antioxidant activities have been explained by DFT-based reactivity descriptors,^10,11^ including the energies of the highest occupied molecular orbital (HOMO) and the lowest unoccupied molecular orbital (LUMO), atomic charges, electron affinity *A*, ionization potential *I*_o_, global hardness *η*, electronegativity *χ*, chemical potential *μ*, global electrophilicity index *ω*, and Fukui chemical parameters.

Based on the DFT theoretical approach of, Janak's theorem, and the finite difference approximation, these descriptors can be proposed by the following eqn (9)–(13).^10,11^

9 *I*_o_ ≈ −*E*_H_

10 *A* ≈ −*E*_L_

11 *η* ≈ (*I*_o_ − *A*)/2 ≈ (*E*_L_ − *E*_H_)/2

12 *χ* ≈ (*I*_o_ + *A*)/2 ≈ (*E*_L_ + *E*_H_)/2

13 *μ* ≈ −(*I*_o_ + *A*)/2 ≈ −(*E*_L_ + *E*_H_)/2

where *E*_H_ and *E*_L_ are the energies of the HOMO and LUMO, respectively.

The global electrophilicity index *ω* indicated the stabilization energy of a molecule system when saturated by electrons from outside. Therefore, the higher value of *ω*^+^ (electron accepting) resulted in significant electrophilicity, while the lower value of *ω*^−^ (electron donating) evidently resulted in better nucleophilicity of a compound. These chemical indices were expressed by the following functions (eqn (14)–(16)).^10,11^

14 *ω* = *μ*^2^/2*η* ≈ (*I*_o_ + *A*)^2^/[4(*I*_o_ − *A*)] ≈ (*E*_L_ + *E*_H_)^2^/[4(*E*_L_ − *E*_H_)]

15 *ω*^−^ = (3*I*_o_ + *A*)^2^/[16(*I*_o_ − *A*)]

16 *ω*^+^ = (*I*_o_ + 3*A*)^2^/[16(*I*_o_ − *A*)]

In general, the condensed Fukui parameters evidently provided information on a selective property in a chemical reaction. The atom coupled with the high electronic population was the most reactive site when compared to the surrounding atoms in a molecule.^10,11^ Briefly, Fukui descriptors have been shown to associate with nucleophilic (*f*_k_^+^), electrophilic (*f*_k_˙^−^), and/or radical attacks (*f*_k_^0^) and were possibly described by the following equilibria (eqn (17)–(19)).^10,11^

17 *f*_k_^+^ = *q*_k_(N + 1) − *q*_k_(N)

18 *f*_k_˙^−^ = *q*_k_(N) − *q*_k_(N − 1)

19 *f*_k_^0^ = [*q*_k_(N + 1) − *q*_k_(N − 1)]/2

where *q*_k_(N): electronic population of atom k in a neutral molecule.*q*_k_(N + 1): electronic population of atom k in an anionic molecule.*q*_k_(N − 1): electronic population of atom k in a cationic molecule.

For radical scavenging actions, the B3LYP functional has always been recommended for either thermodynamics or kinetic calculations.^19,20^ Based on conventional transition state theory (TST),^21^ the rate constant *k* in a radical reaction was described as follows:20
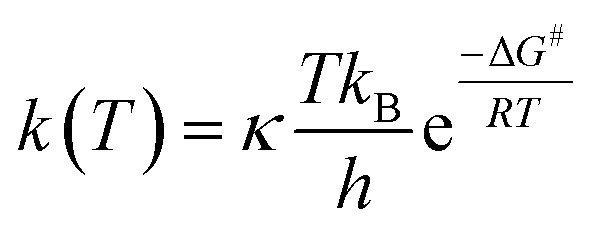
where *κ*, *T*, *k*_B_ and *h* are the Wigner coefficient, temperature, Boltzmann constant and Planck constants, respectively. The Gibbs activation energy Δ*G*^#^ was obtained at 298.15 K and demonstrated the differential energy between reactant and the transition state.

The Wigner coefficient *κ* is also related to the imaginary frequency of the transition state structure *v* (cm^−1^) and the electronic barrier height Δ*E*_B,0_ of a considerable reaction through eqn (21):21
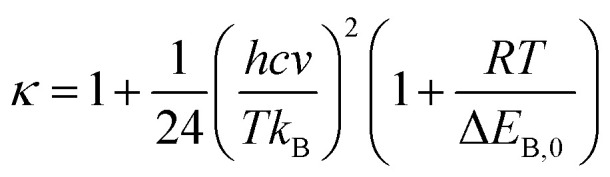


## 3. Results and discussion

### 3.1. Geometrical analysis

The structural differences, molecular size, stereochemistry, length of atomic bonds, and extra- or intramolecular hydrogen bonds, especially in terms of the behavior of hydroxyl groups, can be seen as basic characteristics to prove the pharmacological effects of a studied molecule.^3^ As a consequence, a comprehensive analysis of structural and electronic descriptors of 2-phenylbenzofuran derivatives 1–16 is always recognized as the best way to forecast the radical scavenging capacity, along with the three antioxidant mechanisms. In the current paper, we provide computational outcomes for all studied compounds 1–16 at the DFT-B3LYP/6-311++G(d,p) level. As shown in Fig. S1,† the optimized structure of each compound was similar in all four studied media, and the systematic π-electrons were delocalized throughout the whole molecule, particularly from ring B to ring C through a 2,3-double bond. Optimized stemofurans 1–16 differ in their dihedral angles *θ*_2_ (C3–C2–C1′–C2′) (Table S1†). Interestingly, methylation at carbons C-2′ and/or C-6′ was the main reason why the coplanarity between the benzofuran ring and the phenyl unit of compounds 4–15 was lost, while the dihedral angle *θ*_2_ ≈ 0° was key evidence for the coplanarity of rings AC and B in molecules 1–3 and 16. Taking bond lengths into consideration, the O–H bond lengths of hydroxyl groups at carbons C-4, C-2′, C-4′, and C-5′ fluctuated around 0.962–0.968 Å in the gaseous phase. However, there was a slight increase of 0.001–0.002 Å when the molecules were transferred from gas to solvents. There was no hydrogen bond between 4′-OH and 5′-OH in compound 14, but the O–H bond at these sites may be destabilized because the length reached a maximum 0.966–0.969 Å in the studied media.

**Fig. 2:**
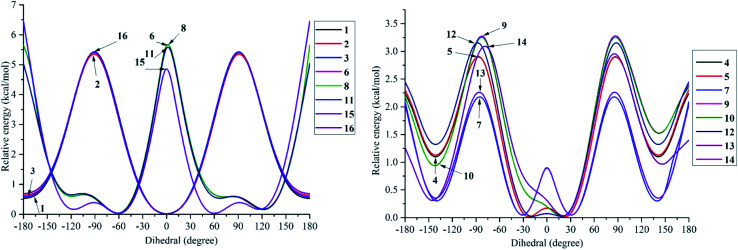
Potential energy curves *versus* the dihedral angle *θ*_2_ (C3–C2–C1′–C2′) in the gaseous medium at B3LYP/6-311G(d,p) level of theory.

To systematically determine the relative position between rings B and AC, potential energy curves *versus* torsional angles *θ*_2_ in the gaseous state were plotted. In all studied compounds 1–16, *θ*_2_ has been explored by scanning from −180° to 180° in 10° increments at the B3LYP/6-31G(d,p) level (Table S2†). For accuracy, without any constraints, molecules 1–16 have thus far been optimized around each conformational potential minimum, and the results are plotted in Fig. 2.

Considering phenylbenzofuran derivatives 1–3 and 16, the local minimum conformers located at ±180° were destabilized by 0.52–0.69 kcal mol^−1^ with respect to the global minimum ones (at 0°). Additionally, the conformational barriers peaked at ±90° with interconversion energies of Δ*E* = 5.45–5.53 kcal mol^−1^ relative to the global conformers.

Likewise, compounds 4–5, 7, 12, and 13 each consisted of global minima at ±20° (or ±30° in 7 and 13) and local minima at ±140°, whereas three barriers were located at ±180°, ±90° (or ±80° in 7), and 0°. Similar behavior can be found in molecules 9–10 and 14. However, there is one small difference, whether they possessed only global minima at 20°. Briefly 2′,4′,6′-trimethylated compounds 6, 8, 11, and 15 also showed the same model of potential energy curves, including two minima conformers and three interchangeable energy barriers (Fig. 2). Due to symmetrical ring B, optimized structure 15 caused slight differences in the energies of local and global conformers, especially energy barriers at ±180° and ±90°, compared with compounds 6, 8 and 11.

### 3.2. Frontier molecular orbital theory and spin density

**Fig. 3:**
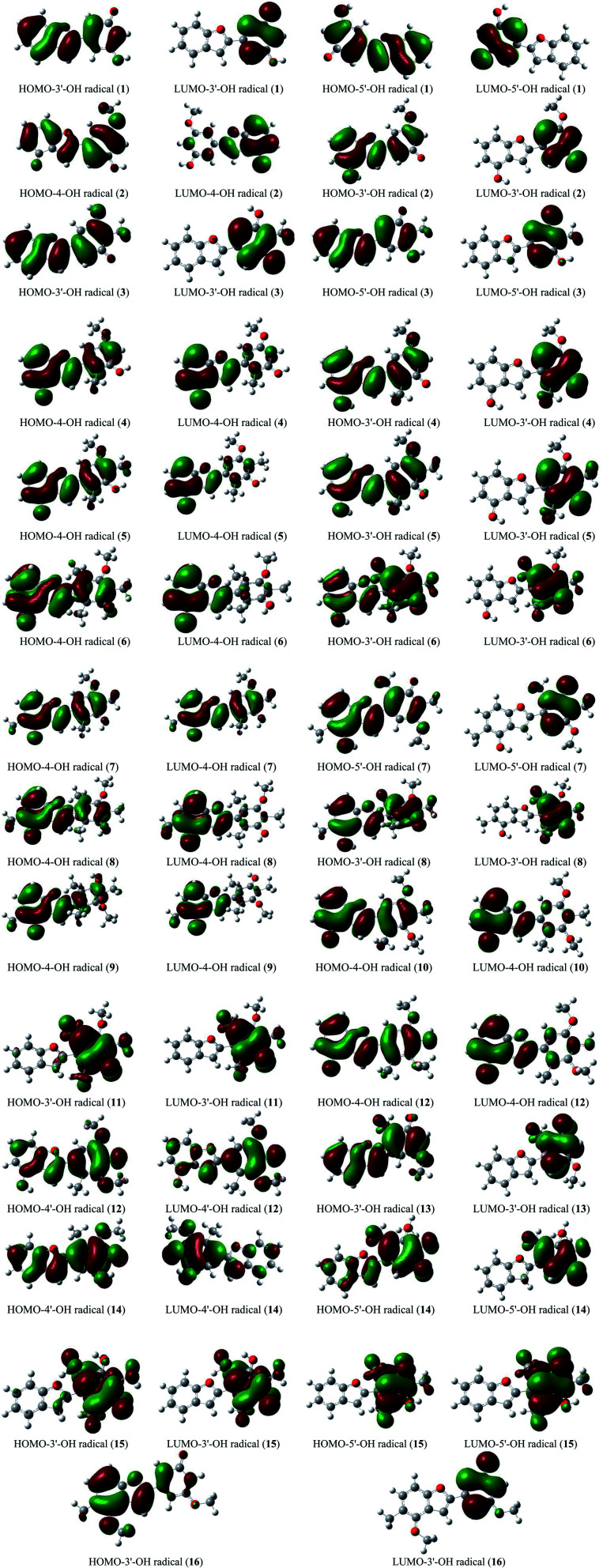
HOMO and LUMO images of structural radicals 1–16 in all studied mediums at B3LYP/6-311++G(d,p) level of theory (Iso-contour value = 0.02).

The stabilization of neutrals and radicals after proton abstraction depends on π-electron delocalization.^9,10^ Considering the frontier molecular orbital distribution would not only help to explain the relationship between neutral and radical forms in terms of the electron contribution but also support the identification of donor–acceptor reactive sites.^22^ At the theoretical level of DFT-B3LYP/6-311++G(d,p), we found that the neutral HOMO and LUMO illustrations of each studied compound were not different when the medium was changed from gas to liquid (Fig. S2†). Additionally, the neutral HOMO and LUMO profiles also showed that the electrons delocalized over the benzofuran and phenyl fragment of each compound, except for the HOMO neutral pictorial representation of stemofuran V (15). The shapes of the radical HOMOs calculated for compounds 1–10, and 12–16 were identical to those of neutral ones (Fig. 3). However, the electrons density was found to be highly concentrated on ring B of HOMO-3′-OH radical (11). This finding suggested that the phenyl units of compounds 11 and 15 facilitated antioxidant reactions rather than the benzofuran nuclei. With regards to the radical LUMO images, in general, the systematic ring AC displayed more charges in the case of 4-OH radicals, whereas this phenomenon was associated with ring B in the case of 3′-OH and 5′-OH radicals.

**Fig. 4:**
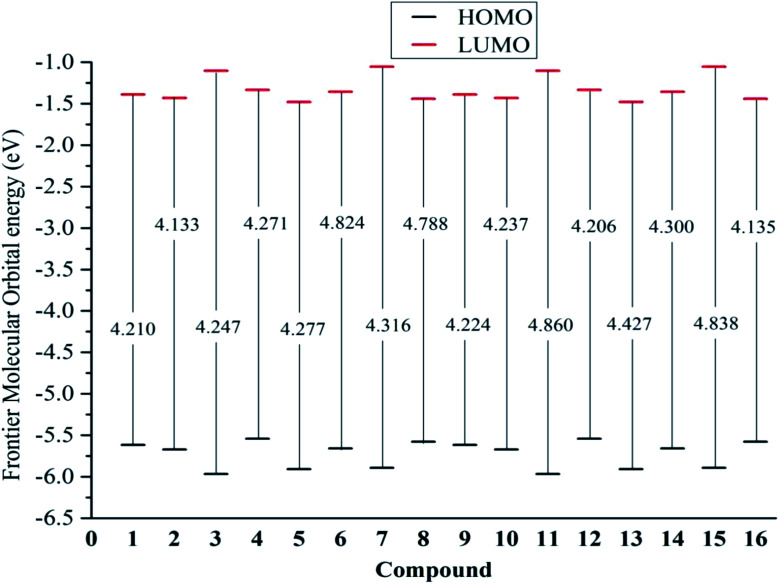
*E*_gap_ = *E*_L_ − *E*_H_ of the neutral structures 1–16 in the gaseous medium at B3LYP/6-311++G(d,p) level of theory.

From eqn (9) and (10), a higher *E*_HOMO_ (the lower ionization potential *I*_o_) and a lower *E*_LUMO_ (the higher electron affinity A) result in a better electron-donating capacity and a better sensitivity to receive electrons, respectively, whereas easier electron transfer indicates a lower *E*_gap_ = *E*_LUMO_ − *E*_HOMO_ and thus better antioxidant reactivity.^9,10^Table 1 reveals that the *E*_HOMO_ values were always higher in the gaseous phase, except for that of stemofuran A (1). Additionally, the *E*_HOMO_ values of 3–16 followed a clear order: gas > acetone > methanol ≥ water. The *E*_LUMO_ values occurred in the following order for all studied compounds: gas > acetone > methanol > water. The *E*_gap_ rates of the studied compounds in the gaseous phase and polar solvents were normally observed to be lower than those in solvents of intermediate polarity. The different energy *E*_gap_ in the gaseous medium between compounds 6, 8, 11, and 15 and the remaining compounds was evidently due to 2′,6′-dimethylation (Fig. 4).

**Table:** Chemical reactivity indices obtained using DFT method in the studied mediums at B3LYP/6-311++G(d,p) level of theory

No	Medium	*η* (eV)	*χ* (eV)	*μ* (eV)	*I*_o_ (eV)	*A* (eV)	*ω* (eV)			Polarizability (au)	Energy (au)	*E*_HOMO_ (eV)	*E*_LUMO_ (eV)	*E*_gap_
							*ω*	*ω* ^−^	*ω* ^+^					
1	Gas	2.105	3.818	−3.818	5.923	1.713	3.462	5.634	1.816	193.493	−765.183	−5.923	−1.713	4.210
	Water	2.000	3.760	−3.760	5.760	1.760	3.534	5.663	1.904	266.904	−765.196	−5.760	−1.760	4.000
	Methanol	2.135	3.891	−3.891	6.026	1.755	3.544	5.756	1.866	264.303	−765.196	−6.026	−1.755	4.271
	Acetone	2.135	3.852	−3.852	5.987	1.717	3.475	5.668	1.816	261.714	−765.195	−5.987	−1.717	4.270
2	Gas	2.066	3.703	−3.703	5.769	1.637	3.318	5.428	1.725	212.048	−879.706	−5.769	−1.637	4.132
	Water	2.077	3.799	−3.799	5.877	1.722	3.474	5.634	1.834	290.958	−879.720	−5.877	−1.722	4.155
	Methanol	2.077	3.795	−3.795	5.872	1.718	3.468	5.625	1.830	288.088	−879.720	−5.872	−1.718	4.154
	Acetone	2.077	3.791	−3.791	5.868	1.714	3.459	5.614	1.824	285.279	−879.719	−5.868	−1.714	4.154
3	Gas	2.123	3.671	−3.671	5.794	1.548	3.173	5.274	1.603	209.319	−804.483	−5.794	−1.548	4.246
	Water	2.123	3.798	−3.798	5.922	1.675	3.397	5.562	1.763	286.801	−804.495	−5.922	−1.675	4.247
	Methanol	2.123	3.792	−3.792	5.915	1.669	3.387	5.549	1.757	284.051	−804.494	−5.915	−1.669	4.246
	Acetone	2.125	3.790	−3.790	5.915	1.664	3.379	5.539	1.749	281.312	−804.494	−5.915	−1.664	4.251
4	Gas	2.135	3.638	−3.638	5.773	1.503	3.099	5.185	1.547	222.587	−919.000	−5.773	−1.503	4.270
	Water	2.158	3.732	−3.732	5.890	1.574	3.227	5.363	1.631	304.796	−919.014	−5.890	−1.574	4.316
	Methanol	2.158	3.728	−3.728	5.886	1.570	3.220	5.354	1.626	301.716	−919.014	−5.886	−1.570	4.316
	Acetone	2.158	3.724	−3.724	5.881	1.566	3.213	5.344	1.621	298.759	−919.013	−5.881	−1.566	4.315
5	Gas	2.138	3.532	−3.532	5.670	1.394	2.917	4.950	1.418	238.055	−958.299	−5.670	−1.394	4.276
	Water	2.158	3.645	−3.645	5.803	1.486	3.077	5.169	1.524	324.439	−958.312	−5.803	−1.486	4.317
	Methanol	2.158	3.639	−3.639	5.798	1.481	3.069	5.158	1.519	321.276	−958.312	−5.798	−1.481	4.317
	Acetone	2.160	3.605	−3.605	5.766	1.445	3.008	5.081	1.475	318.106	−958.311	−5.766	−1.445	4.321
6	Gas	2.412	3.487	−3.487	5.900	1.075	2.521	4.566	1.079	238.523	−997.589	−5.900	−1.075	4.825
	Water	2.423	3.566	−3.566	5.989	1.143	2.624	4.709	1.144	323.164	−997.603	−5.989	−1.143	4.846
	Methanol	2.424	3.561	−3.561	5.986	1.137	2.616	4.699	1.138	319.803	−997.603	−5.986	−1.137	4.849
	Acetone	2.425	3.557	−3.557	5.983	1.132	2.609	4.691	1.133	316.643	−997.602	−5.983	−1.132	4.851
7	Gas	2.158	3.509	−3.509	5.667	1.352	2.854	4.878	1.369	252.800	−997.598	−5.667	−1.352	4.315
	Water	2.170	3.604	−3.604	5.774	1.434	2.993	5.066	1.462	342.233	−997.611	−5.774	−1.434	4.340
	Methanol	2.170	3.600	−3.600	5.770	1.429	2.985	5.056	1.456	338.686	−997.611	−5.770	−1.429	4.341
	Acetone	2.171	3.595	−3.595	5.766	1.425	2.977	5.046	1.451	335.251	−997.610	−5.766	−1.425	4.341
8	Gas	2.394	3.429	−3.429	5.823	1.035	2.456	4.470	1.041	253.782	−1036.889	−5.823	−1.035	4.788
	Water	2.404	3.522	−3.522	5.925	1.118	2.580	4.641	1.119	341.658	−1036.902	−5.925	−1.308	4.617
	Methanol	2.307	3.615	−3.615	5.922	1.308	2.832	4.927	1.313	338.176	−1036.902	−5.922	−1.118	4.804
	Acetone	2.406	3.512	−3.512	5.918	1.106	2.564	4.620	1.108	334.863	−1036.901	−5.918	−1.106	4.812
9	Gas	2.112	3.504	−3.504	5.616	1.392	2.907	4.923	1.419	267.746	−1036.875	−5.616	−1.392	4.224
	Water	2.131	3.623	−3.623	5.754	1.493	3.081	5.159	1.535	361.075	−1036.887	−5.754	−1.493	4.261
	Methanol	2.130	3.619	−3.619	5.749	1.489	3.074	5.150	1.531	357.548	−1036.886	−5.749	−1.489	4.260
	Acetone	2.129	3.614	−3.614	5.743	1.486	3.068	5.142	1.527	354.217	−1036.886	−5.743	−1.486	4.257
10	Gas	2.119	3.552	−3.552	5.670	1.433	2.977	5.018	1.466	251.888	−997.575	−5.670	−1.433	4.237
	Water	2.139	3.655	−3.655	5.793	1.516	3.123	5.217	1.563	342.064	−997.588	−5.793	−1.516	4.277
	Methanol	2.138	3.651	−3.651	5.788	1.513	3.118	5.210	1.559	338.757	−997.587	−5.788	−1.513	4.275
	Acetone	2.136	3.647	−3.647	5.783	1.512	3.114	5.205	1.558	335.536	−997.587	−5.783	−1.512	4.271
11	Gas	2.430	3.536	−3.536	5.966	1.105	2.572	4.643	1.108	234.216	−922.347	−5.966	−1.105	4.861
	Water	2.440	3.656	−3.656	6.096	1.216	2.740	4.873	1.216	317.271	−922.357	−6.096	−1.216	4.880
	Methanol	2.440	3.651	−3.651	6.091	1.210	2.731	4.861	1.210	314.059	−922.357	−6.091	−1.210	4.881
	Acetone	2.440	3.646	−3.646	6.085	1.206	2.724	4.852	1.206	311.010	−922.356	−6.085	−1.206	4.879
12	Gas	2.103	3.439	−3.439	5.542	1.336	2.812	4.794	1.355	244.528	−1033.519	−5.542	−1.336	4.206
	Water	2.114	3.547	−3.547	5.661	1.434	2.976	5.014	1.467	332.309	−1033.533	−5.661	−1.434	4.227
	Methanol	2.113	3.543	−3.543	5.656	1.429	2.969	5.005	1.462	329.030	−1033.533	−5.656	−1.429	4.227
	Acetone	2.113	3.538	−3.538	5.651	1.425	2.962	4.995	1.457	325.849	−1033.532	−5.651	−1.425	4.226
13	Gas	2.214	3.694	−3.694	5.907	1.480	3.082	5.205	1.512	216.181	−843.756	−5.907	−1.480	4.427
	Water	2.219	3.809	−3.809	6.027	1.590	3.270	5.451	1.642	296.452	−843.767	−6.027	−1.590	4.437
	Methanol	2.219	3.804	−3.804	6.022	1.585	3.260	5.439	1.636	293.487	−843.767	−6.022	−1.585	4.437
	Acetone	2.218	3.799	−3.799	6.017	1.580	3.252	5.429	1.630	290.572	−843.767	−6.017	−1.580	4.437
14	Gas	2.150	3.509	−3.509	5.659	1.359	2.863	4.887	1.378	224.268	−918.999	−5.659	−1.359	4.300
	Water	2.142	3.644	−3.644	5.787	1.502	3.099	5.189	1.545	307.391	−919.010	−5.787	−1.502	4.285
	Methanol	2.142	3.638	−3.638	5.781	1.496	3.090	5.177	1.538	304.476	−919.010	−5.781	−1.496	4.285
	Acetone	2.142	3.633	−3.633	5.775	1.490	3.080	5.164	1.531	301.419	−919.009	−5.775	−1.490	4.285
15	Gas	2.419	3.474	−3.474	5.893	1.055	2.494	4.534	1.060	221.060	−883.071	−5.893	−1.055	4.838
	Water	2.427	3.583	−3.583	6.010	1.155	2.644	4.738	1.156	299.087	−883.082	−6.010	−1.155	4.855
	Methanol	2.427	3.577	−3.577	6.004	1.150	2.636	4.728	1.151	296.135	−883.082	−6.004	−1.150	4.854
	Acetone	2.427	3.572	−3.572	5.999	1.145	2.629	4.718	1.146	293.278	−883.081	−5.999	−1.145	4.854
16	Gas	2.067	3.511	−3.511	5.578	1.443	2.981	4.995	1.484	241.717	−958.282	−5.578	−1.443	4.135
	Water	2.068	3.673	−3.673	5.741	1.605	3.262	5.357	1.684	325.948	−958.295	−5.741	−1.605	4.136
	Methanol	2.068	3.667	−3.667	5.734	1.599	3.251	5.342	1.676	324.142	−958.295	−5.734	−1.599	4.135
	Acetone	2.068	3.660	−3.660	5.728	1.593	3.240	5.329	1.668	321.084	−958.294	−5.728	−1.593	4.135

**Fig. 5:**
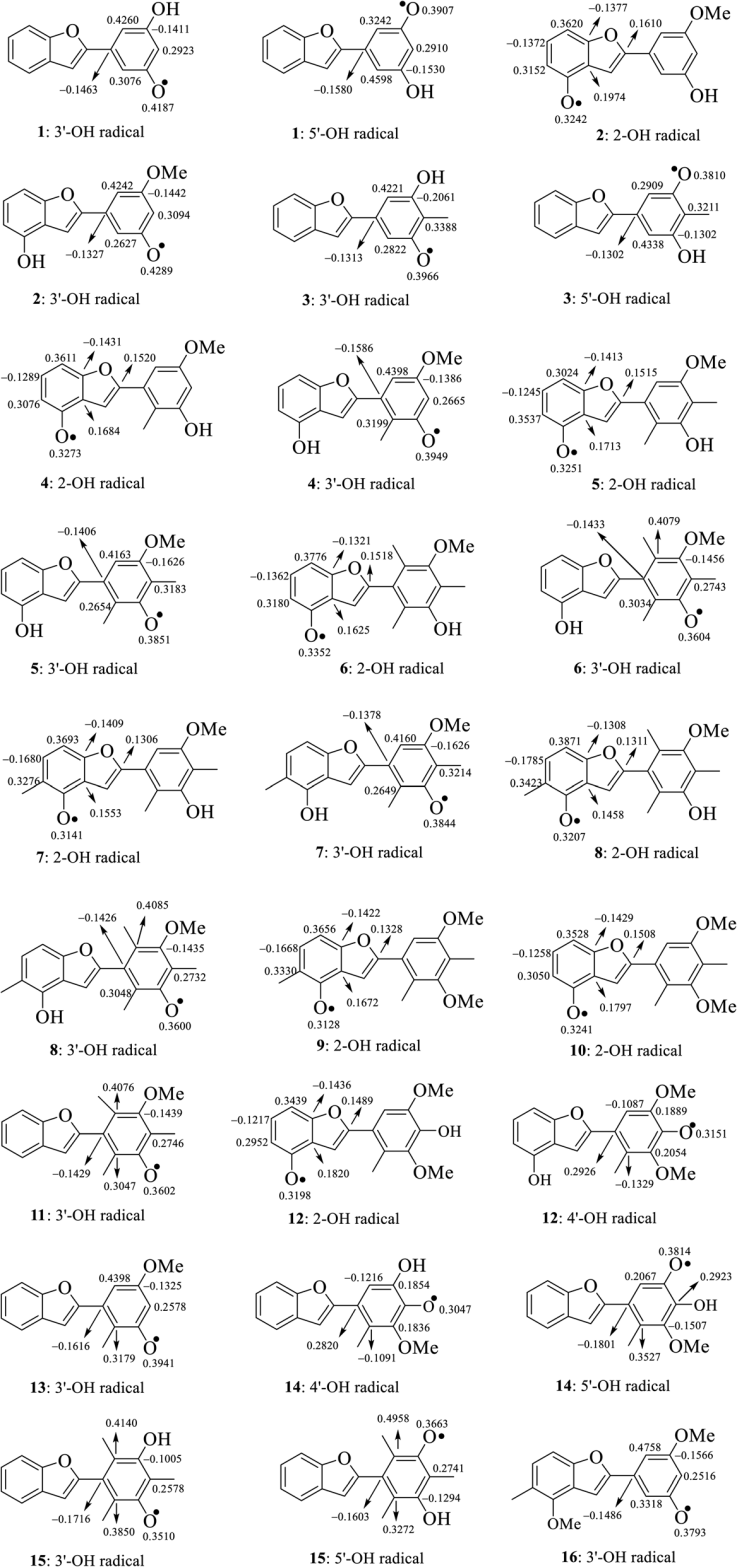
Spin density distribution of structural radicals of compounds 1–16 in the gaseous medium at B3LYP/6-311++G(d,p) level of theory.

The calculated atomic spin density populations of various radicals after H-abstraction from sixteen considered phenylbenzofurans 1–16 in the gaseous phase are available in Fig. 5. It was noted that the more spin density was delocalized on radicals, the more easily the radical formed, thereby resulting in lower BDE values.^9,10^ In general, the computed results revealed that the strong spin distribution in secondary metabolites 1–16 remained on the radical oxygen atoms, aromatic carbons of ring B, carbon C-3a and fragment C-5–C-6–C-7–C-7a of ring A, and C-2 of ring C but had a deeper dependence on the medium used. Among carbons with significant spin density values, a consistent rule was found: 4-OH radicals had of negative spin density at carbons C-6 and C-7a and positive spin density at carbons C-3a, C-5, C-7 and C-2. In the cases of 3′-OH and 5′-OH radicals, ring B was stabilized with negative spin density on carbons C-1′, C-3′, and C-5′ and positive spin density on carbons C-2′, C-4′ and C-6'. Of the 4′-OH radicals of compounds 12 and 14, the stabilization of ring B may be due to the negative spin density at carbons C-2′, and C-6′ and the positive spin density at C-1′, C-3′ and C-5'. This result suggested that ring B radicals were stabilized more than ring A radicals and that the phenyl unit of ring B was a suitable site for radical formation.

We continued to discuss the gaseous spin density values at O-atom radicals, and the lower spin density values might be related to the lower BDE values. First, the studied compounds caused by di- or tri-methylation at ring B were shown to have lower spin densities than the groups of compounds formed by mono-methylation and without substitution. The most striking feature is that 4-O˙ and 4′-O˙ gave rise to lower spin density than 3′-O˙, and 5′-O˙. As a representative example, compound 14 4′-O˙ radicals induced the lowest spin density of 0.3047, in contrast to the largest values for compounds 1 3′-O˙ (0.4187) and 5′-O˙ (0.3907), compound 2 3′-O˙ (0.4289), compound 4 3′-O˙ (0.3949), and compound 13 3′-O˙ (0.3941). Therefore, as expected, the gaseous BDE values varied correspondingly.

### 3.3. Electronic properties

The global hardness *η* has been employed to measure the resistance to charge transfer.^9,10^ Most compounds possessed higher *η* values in solvents than in the gas phase. In accordance with the gaseous *E*_gap_ energy, the *η* descriptor of compounds 6, 8, 11 and 15 increased considerably due to 2′,4′,6′-trimethylation. Table 1 also shows the difference in polarizability. All studied compounds in the polar solvents water and methanol always had larger polarizabilities than those in intermediate-polarity acetone or nonpolar gas. According to the theoretical hard and soft acids and bases (HSAB) rule, it can be concluded that environmental factors can reasonably induce a change from “high oxidation state and low polarizability” to “low oxidation state and high polarizability”.^23^

The electronegativity *χ* describes the tendency of an atom to attract electrons towards itself, and the chemical potential *μ* is equivalent to this parameter with a negative sign.^24^ According to Sanderson's theory, a compound with a high electronegativity is associated with a low reactivity.^23^ Therefore, it is worth noting that a lower value of *χ* is better for antioxidant reactions. In all studied compounds, a gaseous medium was used to reduce *χ* rather than acidic environments of methanol and acetone. It should also be noted that stemofurans 5–12 and 14–15 mostly yielded lower electronegativity *χ* values than the other compounds in the four studied media (Table 1).

In addition to descriptors such as electron affinity, ionization potential, global hardness, and global electronegativity, increases in the global electrophilicity indices *ω*, *ω*^−^, and *ω*^+^ have further been observed when a gas is replaced by high-polarity solvents. The *ω*^−^ values of molecules 1–16 were approximately 3–3.5 times higher than the *ω*^+^ values in all phases. This finding was identical to the results of previous DFT studies of other phenolic compounds, of which phenylbenzofurans 1–16 tended to donate rather than capture electrons.^9,10^

In addition to the frontier molecular orbital consideration and analysis of electronic structure, Fukui indices are also among the most important parameters and a quick method to analyze the powerful reactive site of each atom. In general, electrophilicity has been shown to be associated with Δ*f*_k_ = *f*_k_^+^ − *f*_k_˙^−^ > 0. However, Δ*f*_k_ < 0 was associated with nucleophilic character.^25^ The Fukui indices given in Tables S3–S4† were calculated in a gaseous medium based on the theoretical HSAB principle. Interestingly, it was concluded that in phenylbenzofurans 1–16, aromatic carbons should serve as good sites for both electrophilic and nucleophilic attacks. Furthermore, the hydroxyl groups at carbons C-4, C-4′, and C-5′ served as marginal electrophiles.^26^ In the same manner, 3′-OH served as nucleophilic sites in molecules 1–2, 4–5, 9–10, and 12–15 and showed marginal electrophilic properties in the remaining compounds.^27^ Most importantly, the negative *f*_k_^0^ condensed Fukui values suggested the production of radicals. All studied compounds were characterized by negative values at the oxygen atoms of the hydroxyl and methoxyl groups. It is expected that radical scavenging reactions deriving from hydroxyl sites are easier.

Finally, we discuss the electronic features of stemofurans 1–16 by using a molecular electrostatic potential model. At different points on the electron density isosurface contours, the electrostatic potential is represented by different colors. The potential clearly increased in the order red < orange < yellow < green < blue, in which blue indicates the zone of the most positive electrostatic potential, red and orange indicate areas of the most negative potentials, and green indicates regions with zero potential.^28^ In rings A and B of stemofurans 1–16, oxygen was represented by yellow, and blue and green indicated hydrogens and methyl groups (Fig. S3†). This finding, once again, indicates that the hydroxyl groups of 2-phenylbenzofurans serve as nucleophiles.^28^

### 3.4. NMR spectroscopy

NMR (nuclear magnetic resonance) spectroscopy was extensively used for the structural analysis of naturally occurring and synthetic organic compounds. An advantage was that the combination of the experimental NMR data and quantum chemical DFT calculations was an exceptional tool to elucidate the structure and determine molecular conformations. Currently, available methods have been employed to calculate chemical shifts, *e.g.*, IGLO (individual gauge localized orbital), but GIAO (gauge-independent or invariant or including atomic orbitals) has provided high accuracy with different nuclei.^9,10^ In the current account, the GIAO procedure provided the ^1^H and ^13^C-NMR computational spectra of optimized structures 1–12 in acetone-*d*_6_ and 13–16 in CDCl_3_ using corresponding TMS shielding to estimate the chemical shifts as *δ* (ppm) = *δ*_calc(TMS)_ − *δ*_calc_ (*σ* is the absolute shielding constant).^9,10^ As shown in Tables S5–S6,† at the B3LYP/6-311++G(d,p) level, the model NMR prediction was in good agreement with the experimental results, in which the observed and calculated ^1^H-NMR chemical shifts of each compound established correlation values *R*^2^ ≥ 0.9860, while similar results of *R*^2^ ≥ 0.9929 were found for the ^13^C-NMR chemical shifts of compounds 2–7, 9–10, and 13–15. In all studied compounds, our calculation indicated that nonsubstituted positions of systematic ring AC were generally observed at chemical shifts of *δ*_H_ 6.64–7.66 ppm (H-3)/*δ*_C_ 100.0–112.9 ppm (C-3), *δ*_H_ 7.25–7.57 ppm (H-6)/*δ*_C_ 128.2–132.1 ppm (C-6), and *δ*_H_ 7.08–7.77 ppm (H-7)/*δ*_C_ 105.4–115.3 ppm (C-7). The methyl group in compounds 3–16 was observed at chemical shifts of *δ*_H_ 1.85–2.55 ppm/*δ*_C_ 5.69–17.6 ppm, while the NMR-predicted values of the methoxyl group of 2, 4–14, and 16 were *δ*_H_ 3.63–4.42 ppm/*δ*_C_ 55.5–61.3 ppm. Significantly, we fully assigned the ^13^C-NMR data of stemofuran K (11), as well as several values in compounds 1, 8, 12, and 16 (Table 4), since the experimental values did not provide complete identification.

### 3.5. UV spectroscopy

**Fig. 6:**
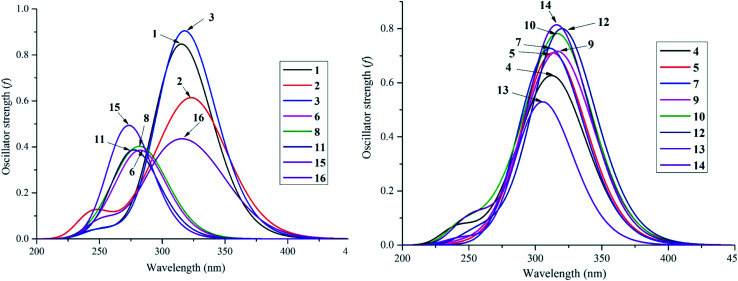
UV-Vis absorption spectroscopies of studied compounds 1–16 in methanol medium at TD-DFT/B3LYP/6-311++G(d,p) level of theory.

Despite the fact that experimental UV-visible spectra of almost all isolated compounds are now available, the calculation procedure has not yet been performed, to the best of our knowledge.^4,6,29^ We continue to provide quantum analytical results to achieve a detailed comparison between theoretical and experimental results. Utilizing a reliable time-dependent density functional theory (TD-DFT) method at the B3LYP/6-311++G(d,p) level to predict the UV spectra of polyphenolic derivatives,^10,11^ the observed (in methanol) and the predicted (in gas and methanol) electronic excitations (energy, wavelength, oscillator and transition assignment) of stemofurans 1–16 are presented in Fig. 6 and Table 2. With reference to the experimental values, the theoretical outcomes using methanol as the solvent normally showed higher accuracy than those in a gas medium. For example, because of the H → L (98%) transition, the calculated UV-spectral data of stemofuran O (14) exhibited an absorption band at *λ*_max_ of 317 nm (*E*_vert_ = 3.913 eV, and *f* = 0.875) in methanol, in contrast to those of the gaseous phase [*λ*_max_ of 309 nm, *E*_vert_ = 4.010 eV, *f* = 0.723, and H → L (95%)] and the experimental *λ*_max_ of 323 nm. However, according to the TD-DFT method, the UV spectra of stilbenoids 1–16 were composed of one main broad peak at approximately *λ*_max_ 320 nm for compounds 1–5, 7, 9–10, and 12 and at approximately *λ*_max_ 300 nm for compounds 6, 8, 11, 13, and 15–16 due to the H → L excited transition in both studied media. The difference in *λ*_max_ values can be explained by the number and positions of CH_3_ groups, especially the large number of such groups in compounds 6, 8, 11, and 15. Furthermore, in most cases, H-1 → L and H-2 → L transitions are responsible for the two small remaining peaks (*λ*_max_ values were found to be less than that of the main peak) (Table 2). Furthermore, regarding phenylbenzofurans 1–16, based on the solvent influence, all *λ*_max_ values were redshifted and were attributed to π → π* natural electronic excitations on the whole molecule.

**Table:** Selectively experimental (methanol) and calculated electronic transitions (methanol and gas) of the studied compounds 1–16: wavelength *λ*_max_ (nm), vertical transition energy *E* (eV), oscillator strength *f*, at TD-DFT/B3LYP/6-311++G(d,p) level of theory

No	Exp.	Calculated								
		Methanol				Gas				Transition type
	*λ*_max_ (nm)	*λ*_max_ (nm)	*E*_vert_ (eV)	Oscillator strength *f*	Transition	*λ*_max_ (nm)	*E*_vert_ (eV)	Oscillator strength *f*	Transition	
1	318	317	3.917	0.870	H → L (98%)	310	4.000	0.688	H → L (94%)	π → π*
	304	302	4.110	0.074	H-1 → L (90%)	300	4.137	0.113	H-1 → L (85%)	
	286	275	4.504	0.029	H-2 → L (81%)	275	4.503	0.021	H-2 → L (81%)	
2	326	328	3.778	0.663	H → L (98%)	325	3.818	0.472	H → L (94%)	
	308	301	4.113	0.080	H-1 → L (90%)	300	4.135	0.065	H-1 → L (89%)	
	298	291	4.256	0.185	H-2 → L (89%)	292	4.244	0.238	H-2 → L (87%)	
3	320	319	3.892	0.969	H → L (98%)	311	3.986	0.814	H → L (96%)	
	306	301	4.120	0.052	H-1 → L (89%)	299	4.149	0.065	H-1 → L (86%)	
	278	273	4.541	0.023	H-2 → L (73%)	273	4.536	0.018	H-2 → L (75%)	
4	292	317	3.910	0.621	H → L (95%)	315	3.930	0.455	H → L (92%)	
		304	4.080	0.111	H-1 → L (91%)	303	4.088	0.073	H-1 → L (91%)	
		284	4.364	0.172	H-2 → L (87%)	286	4.331	0.235	H-2 → L (86%)	
5	324	316	3.918	0.741	H → L (95%)	314	3.949	0.571	H → L (93%)	
	306	302	4.101	0.122	H-1 → L (90%)	301	4.112	0.078	H-1 → L 89%)	
	292	283	4.374	0.112	H-2 → L (85%)	286	4.333	0.190	H-2 → L (86%)	
6	290	287	4.327	0.403	H → L (89%)	286	4.332	0.302	H → L (87%)	
	258	278	4.456	0.083	H-1 → L (84%)	278	4.456	0.039	H-1 → L (84%)	
	220	263	4.719	0.107	H-2 → L (65%)	263	4.718	0.124	H-2 → L (69%)	
7	326	315	3.933	0.754	H → L (97%)	312	3.971	0.564	H → L (94%)	
	296	298	4.156	0.055	H-1 → L (89%)	298	4.163	0.037	H-1 → L (72%)	
		291	4.264	0.185	H-2 → L (88%)	293	4.237	0.237	H-2 → L (69)	
8		289	4.294	0.400	H → L (93%)	289	4.297	0.277	H → L (92%)	
		279	4.443	0.023	H-1 → L (89%)	280	4.436	0.021	H-1 → L (90%)	
		268	4.626	0.208	H-2 → L (79%)	268	4.630	0.201	H-2 → L (78%)	
9	328	321	3.862	0.775	H → L (97%)	318	3.894	0.583	H → L (93%)	
	312	298	4.165	0.070	H-1 → L (91%)	299	4.145	0.127	H-1 → L (89%)	
	298	289	4.297	0.193	H-2 → L (85%)	289	4.283	0.178	H-2 → L (78%)	
10	326	320	3.880	0.780	H → L (97%)	317	3.917	0.620	H → L (95%)	
	312	294	4.211	0.061	H-1 → L (89%)	295	4.205	0.032	H-1 → L (87%)	
	294	282	4.391	0.152	H-2 → L (82%)	285	4.355	0.191	H-2 → L (78%)	
11	282	287	4.318	0.227	H → L (89%)	287	4.316	0.162	H → L (85%)	
	276	274	4.523	0.437	H-1 → L (83%)	272	4.561	0.372	H-1 → L (78%)	
	256	257	4.832	0.021	H-2 → L (40%)	257	4.822	0.021	H → L+1 (61%)	
12	326	321	3.864	0.863	H → L (98%)	316	3.922	0.721	H → L (97%)	
	312	287	4.328	0.019	H-1 → L (77%)	288	4.305	0.054	H-1 → L (84%)	
	298	282	4.390	0.062	H → L (62%)	285	4.345	0.035	H → L (61%)	
13	310	308	4.024	0.594	H → L (84%)	305	4.070	0.207	H-1 → L (54%); H → L (41%)	
	298	301	4.124	0.290	H-1 → L (80%)	299	4.148	0.524	H-1 → L (39%); H → L (57%)	
	254	269	4.604	0.016	H-2 → L (72%)	269	4.615	0.013	H-2 → L (68%)	
14	323	317	3.913	0.875	H → L (98%)	309	4.010	0.723	H → L (95%)	
	286	286	4.335	0.055	H-1 → L (65%)	286	4.332	0.066	H-1 → L (54%)	
	277	270	4.599	0.032	H → L (50%)	270	4.596	0.025	H → L (58%)	
15	284	291	4.257	0.030	H → L (95%)	292	4.246	0.027	H → L (95%)	
	277	274	4.527	0.567	H-1 → L (94%)	274	4.522	0.491	H-1 → L (93%)	
	255	254	4.879	0.025	H-3 → L (33%); H-2 → L (13%); H-1 → L+1 (17%); H-1 → L+3 (15%)	255	4.855	0.016	H-3 → L (29%); H-2 → L (26%)	
16	304	333	3.726	0.494	H → L (96%)	329	3.771	0.335	H → L (91%)	
	296	299	4.140	0.283	H-1 → L (59%)	298	4.159	0.345	H-1 → L (55%)	
	230	254	4.885	0.151	H → L+2 (69%)	252	4.911	0.146	H-1 → L+4 (67%)	

### 3.6. Antioxidant mechanisms

#### 3.6.1. HAT mechanism

Apparently, the HAT pathway occurred when a spontaneous H atom was extracted from the hydroxyl group and produced a free 2-phenylbenzofuran radical since the process of bond dissociation was essentially driven by the physicochemical enthalpy BDE.

As shown in Table 3, in each radical of compounds 1–16, the lowest BDE values were always found in water, while the nonpolar gas medium resulted in the second lowest values rather than the intermediate-polarity solvent acetone. This result indicates that radical reactions may be thought of as a consequence of heterolytic (intracellular environment) and homolytic processes (heat beam or UV attacks).^10,11,29^

Studied phases reaction enthalpies at 298 K for radicals of the studied compounds 1–16 at B3LYP/6-311++G(d,p) level of theory (in kcal mol^−1^)

**Table:** 

Compounds	BDE				IP				PDE			
	Gas	Water	Methanol	Acetone	Gas	Water	Methanol	Acetone	Gas	Water	Methanol	Acetone
1					168.89	108.46	113.71	107.02				
3′-OH	83.37	82.42	83.85	84.33					230.29	83.37	16.24	7.88
5′-OH	84.33	81.46	84.10	84.57					231.25	84.33	16.48	8.12
2					164.59	105.11	110.13	102.96				
4-OH	76.44	74.77	76.92	76.68					227.66	76.45	12.66	4.30
3′-OH	84.33	82.66	84.81	84.81					235.55	84.33	20.78	12.42
3					164.34	105.83	110.85	104.16				
3′-OH	81.22	79.79	81.70	82.89					232.68	81.22	16.72	9.32
5′-OH	81.46	79.55	81.94	82.89					232.92	81.46	16.96	9.32
4					163.88	105.11	110.37	102.96				
4-OH	76.44	74.77	76.92	76.92					228.62	76.45	12.66	4.54
3′-OH	81.70	80.03	82.18	81.94					233.64	81.70	17.68	9.56
5					160.53	102.96	107.98	100.81				
4-OH	76.44	74.53	76.68	76.44					231.49	76.45	14.57	6.45
3′-OH	79.07	77.64	79.79	79.55					234.35	79.07	17.68	9.56
6					163.88	105.59	110.61	103.44				
4-OH	76.92	75.25	77.40	77.16					228.62	76.92	12.66	4.54
3′-OH	77.16	75.73	77.88	77.88					228.86	77.16	13.14	5.02
7					159.34	102.00	107.26	99.86				
4-OH	74.30	72.86	75.01	75.01					230.53	74.30	13.86	5.73
3′-OH	78.35	77.16	79.31	79.07					234.59	78.36	18.16	9.79
8					162.21	104.40	109.65	102.25				
4-OH	74.77	73.34	75.49	75.49					228.38	74.77	11.94	3.82
3′-OH	77.16	75.73	77.88	77.64					230.53	77.16	14.33	5.97
9					158.38	101.77	106.78	99.62				
4-OH	74.29	72.86	75.01	74.77					231.72	74.30	14.09	5.97
10					160.06	102.72	107.74	100.57				
4-OH	76.20	74.53	76.68	76.44					231.96	76.21	14.81	6.69
11					164.83	107.50	112.52	105.11				
3′-OH	77.16	75.73	77.88	77.64					227.90	77.16	11.23	3.11
12					156.47	99.38	104.63	97.23				
4-OH	75.97	74.06	76.20	76.20					235.31	75.97	17.68	9.56
4′-OH	76.20	72.86	75.25	75.25					235.31	76.21	16.48	8.60
13					166.51	107.74	112.76	105.59				
3′-OH	80.50	79.31	81.46	81.22					229.81	80.51	14.57	6.21
14					160.54	102.48	107.74	100.33				
4′-OH	71.66	70.95	73.82	73.17					226.71	71.67	12.18	4.06
5′-OH	82.66	77.40	80.50	80.50					237.94	82.66	18.87	10.75
15					165.55	107.50	113.00	105.59				
3′-OH	77.88	75.72	77.88	77.64					228.14	77.88	10.75	2.63
5′-OH	76.44	75.01	77.16	76.92					226.71	76.45	10.03	1.91
16					159.34	102.01	107.02	107.02				
3′-OH	82.66	80.74	82.18	82.18					239.13	82.66	21.02	12.90

**Table:** 

Compounds	PA				ETE			
	Gas	Water	Methanol	Acetone	Gas	Water	Methanol	Acetone
1								
3′-OH	337.79	46.82	43.96	37.98	61.40	82.90	85.76	76.92
5′-OH	338.27	46.82	44.19	38.22	61.87	82.18	86.00	77.16
2								
4-OH	335.16	45.39	42.52	36.07	57.10	76.68	80.27	71.43
3′-OH	337.79	48.02	45.15	37.98	62.35	82.18	85.76	77.40
3								
3′-OH	337.31	47.54	44.91	39.42	59.48	79.55	82.90	74.06
5′-OH	337.79	47.54	44.67	39.18	59.48	79.31	83.13	74.30
4								
4-OH	334.69	45.87	43.00	36.31	57.57	76.45	80.03	71.19
3′-OH	338.03	48.73	45.87	39.18	59.48	78.60	82.18	73.34
5								
4-OH	336.36	46.11	43.24	36.55	55.66	75.97	79.55	70.47
3′-OH	337.55	49.69	46.82	40.13	57.33	75.25	78.83	70.00
6								
4-OH	336.60	46.11	43.72	37.03	55.90	76.68	79.55	70.71
3′-OH	338.27	50.17	47.30	40.85	54.71	73.10	76.45	67.61
7								
4-OH	334.93	46.82	43.48	37.27	55.18	73.34	76.92	68.08
3′-OH	337.31	49.45	46.58	39.89	56.86	75.01	78.83	70.00
8								
4-OH	336.36	47.54	44.67	37.98	54.23	73.34	76.92	68.08
3′-OH	338.03	50.17	47.30	40.61	54.71	72.86	76.45	67.61
9								
4-OH	335.40	46.82	43.96	37.27	54.71	73.34	76.92	68.08
10								
4-OH	335.64	46.11	43.24	36.55	56.38	75.97	79.55	70.71
11								
3′-OH	339.23	50.17	47.54	40.85	53.75	72.86	76.45	67.37
12								
4-OH	336.36	46.34	43.48	36.79	55.18	75.25	78.83	70.00
4′-OH	335.40	47.78	45.15	38.46	56.38	72.62	76.21	67.37
13								
3′-OH	338.51	48.26	45.39	38.70	57.81	78.60	81.94	73.10
14								
4′-OH	327.76	42.76	40.37	33.68	59.72	75.49	79.55	70.71
5′-OH	344.24	48.97	48.02	41.33	54.23	75.73	78.60	69.76
15								
3′-OH	340.18	50.41	47.54	41.09	53.27	72.62	76.21	67.37
5′-OH	338.99	50.17	47.30	40.61	53.27	72.38	75.97	66.89
16								
3′-OH	338.99	48.26	45.39	38.94	59.48	79.79	82.66	73.82

In detail, the results showed good agreement with the findings of 4′-OH bond length and spin density, in which the stemofuran U (14) 4′-OH radical had the smallest enthalpy BDE values of 71.66 kcal mol^−1^ and 70.95 kcal mol^−1^ in gas and water, respectively. The agreement between spin density and BDE was further observed when the hydroxyl radicals at carbon C-4 or C-4′ of 2, 4–8, and 14 often exhibited lower BDE values than those at carbon C-3′ or C-5′ in all four studied media.

Stemofuran S (12) 4′-OH radicals exhibited a lower gaseous BDE value than 4-OH, but the opposite phenomenon was observed in solvents. 4′-Hydroxylation was also required for the lower BDE in the stemofuran S (12) 4-OH radical case when compared with that of the stemofuran J (10) 4-OH radical case (4′-methylation). For compounds 12 and 14 that only differed in functional groups substituting at carbons C-4 and C-5′, the reactive BDE of the stemofuran U (14) 4′-OH radical was always lower than that of stemofuran S (12) 4′-OH in all studied media. This finding resembled the cases of compounds 5 and 7 and of compounds 6 and 8; in comparison, for compounds 9 and 10, 5-methylation was responsible for reducing the BDE value of the stemofuran I (9) 4-OH radical by an average of 1.67 kcal mol^−1^ in all studied media. Similarly, among 11, 13, and 16, the stemofuran K (11) 3′-OH radical was recognized to have the lowest BDE values, thereby suggesting that methylation at carbons C-2′, C-4′, and C-6′ induced a positive signal, whereas the introduction of 4-OCH_3_ and 5-CH_3_ cannot be considered as such. In the same manner, because of methylation at ring B, stemofuran V (15) 3′-OH and 5′-OH radicals yielded the lowest BDE values in all studied media compared with the corresponding cases in stemofurans A (1) and C (3). The structure and bioactivity relationship has also been observed from a comparison among compounds 2, and 4–6; the 3′-OH radical BDE values followed the clear order stemofuran F (6) < stemofuran E (5) < stemofuran F (4) < stemofuran B (2). This finding, once again, encouraged the introduction of a methyl group onto ring B to achieve a lower BDE value. Finally, upon 6′-methylation, the same result was obtained, with the 3′-OH radical BDE value of stemofuran H (8) < the 3′-OH radical BDE value of stemofuran G (7). Coplanarity would help to delocalize electrons in compounds 1–3 and 16, thereby resulting in the highest BDE values from 3′-OH and/or 5′-OH. Finally, our results also suggested that 2-phenylbenzofurans 1–16 are promising antioxidant agents because their BDE values were comparable to those of flavonoids or other analogous phenolics.^10,11,30^

#### 3.6.2. SET-PT and SPLET mechanisms

It is known that in solvent environments, SET-PT and SPLET mechanisms might play important roles in the free radical scavenging reaction of natural products.^10,11^ As mentioned above, these two pathways consist of two steps but are preferentially determined by the first step.^31^ As a consequence, the calculated IP values appeared to indicate the SET-PT method. From Table 3, it is confirmed that the polarity of the solvent greatly affected the charged species. The enthalpy IP values of compounds 1–16 in the liquid phase ranged from 97.23 kcal mol^−1^ to 113.71 kcal mol^−1^ and were less than those in the gaseous phase (156.47–168.89 kcal mol^−1^). In addition, the order of IP values for each compound was as follows: acetone < water < methanol ≪ gas. Therefore, it is assumed that if the SET-PT mechanism occurs, acetone and water solvents are the best choices for antioxidant activity. The largest IP values were assigned to stemofuran A (1); in contrast, the lowest IP values were found for stemofuran S (12). Considering the structure-bioactivity relation, 2-phenylbenzofurans with 3′ and/or 5′-hydroxylations, such as compounds 1, 3, 11, 13, 15, and 16, evidently possessed larger IP values than the remaining compounds in all media. Similar to the BDE analysis, 5-methylation continued to be a major reason for decreasing the IP values between compounds 5 and 7, between compounds 6 and 8, and between 9 and 10. However, methylation at ring B did not promote a decrease in IP values, such as in compound 8 in all studied media, which was shown to have larger IP enthalpies than compound 7 due to 6′-methylation. Compound 6 did not have the lowest IP values among compounds 2 and 4–6, inconsistent with the BDE outcome described above. Herein, the IP values were found to follow the order stemofuran C (3) < stemofuran V (15) < stemofuran A (1).

The second step of SET-PT was the deprotonation of the radical cation and had normally been defined by the lowest PDE. The intermediate-polarity solvents acetone and methanol drastically decreased the PDE values in comparison with nonpolar gas and strongly polar water, in which the PDE values of each radical always followed the order acetone < methanol < water ≪ gas. Both the 3′-OH and 5′-OH radicals of stemofuran V (15) in acetone achieved the lowest PDE values of 2.63 kcal mol^−1^ and 1.91 kcal mol^−1^, respectively. Furthermore, the largest acetone PDE values of 12.42 kcal mol^−1^ and 12.90 kcal mol^−1^ were observed for the 3′-OH radicals of compounds 2 and 16, respectively. In all studied media, the 4-OH radical PDE values of three pairs of compounds 5 and 7, 6 and 8, and 9 and 10 showed the same trend as the BDE analysis when 5-methylation was taken into account. The next evidence indicated the same tendency between BDE and PDE enthalpies that methylation of ring B was responsible for the order of the PDE values: compound 2 3′-OH radical > compounds 4–5 3′-OH radicals > compound 6 3′-OH radical; compound 7 4-OH radical > compound 8 4-OH radical; and compound 13 3′-OH radical > compound 11 3′-OH radical.

The SPLET mechanism was taken into account. In the first step of this process, the PA value was quite sensitive to changes in the environment. It was clear that the deviation in the PA values reached up to 7.0–9.0 times between the gaseous medium and the use of solvents in all radical cases. Similar to the trend of the PDE outcomes, the order of the PA values was as follows: acetone < methanol < water ≪ gas.

The stemofuran U (14) 4′-OH radical not only exhibited the lowest BDE values but also revealed the lowest PA values of 40.37 kcal mol^−1^ and 33.68 kcal mol^−1^ in methanol and acetone, respectively. The trend of PA enthalpies was opposite the trend of BDE values in the radical cases of similar compounds due to the effect of the hydrophobic methyl group. For instance, 5-methylation at ring A could be the main reason for the different PA values between compound 9 4-OH radical and compound 10 4-OH radical, or considering the important role of 2′,4′,6′-trimethylation at ring B, the PA energy values of the 3′-OH and 5′-OH radicals increased dramatically when compound 1 was modified as compound 15 in polar solvents.

In the second step, the electron transfer enthalpy (ETE) values exhibited an order of gas < acetone < methanol in each of the investigated radicals, and the lowest values of 53.75 kcal mol^−1^ (gas), 72.86 kcal mol^−1^ (water), 76.45 kcal mol^−1^ (methanol), and 67.37 kcal mol^−1^ (acetone) were derived from stemofuran K (11) 3′-OH radicals. The ETE value of the 3′-OH radical was not substantially different from that of the 5′-OH radical in compounds 1, 3, and 15 in all studied media, but radicals of stemofuran V (15) with a higher number of methyl groups were recognized to have the lowest ETE values. The difference between the number of methyl groups and the ETE values was also observed in the 3′-OH radicals of compounds 7 and 8 and compounds 11 and 13 and especially among compounds 2 and 4–6. Due to 5-methylation, we can identify the same trend for BDE and ETE calculations by the last piece of evidence, in which hydroxyl radicals at ring A of stemofurans G (7), H (8) and I (9) displayed ETE values lower than those of stemofurans E (5), F (6) and J (10), respectively.

#### 3.6.3. Preferential mechanisms

The favorable antiradical mechanisms of stemofurans 1–16 might be discussed in terms of the thermodynamically preferential BDE values of HAT, IP values of SET-PT, and PA values of SPLET.^31^ A minimal value of BDE, IP, an PA indicated that the mechanism occurred easily. It should be noted that a low BDE enthalpy is related directly to radical scavenging. The same applies to IP value. However, PA value is characteristic of anions forming, therefore SPLET represents for the radical scavenging potency. From Table 3, as seen by analyzing the enthalpies of reactions of all studied compounds, the BDE values are always lower than the IP and PA values in the gaseous phase and occurred in the order BDE < IP < PA. Consequently, the thermodynamically preferred mechanism HAT is dominant for stemofurans 1–16 in a gas environment.^32^ Regarding the application of liquids, stemofurans 1–16 have been documented to exhibit PA enthalpies approximately 2–2.5 times lower than the BDE and IP values, so the SPLET mechanism is the most likely reaction in water, methanol, and acetone.^32^

#### 3.6.4. The kinetic reactions of DPPH radical scavenging

**Fig. 7:**
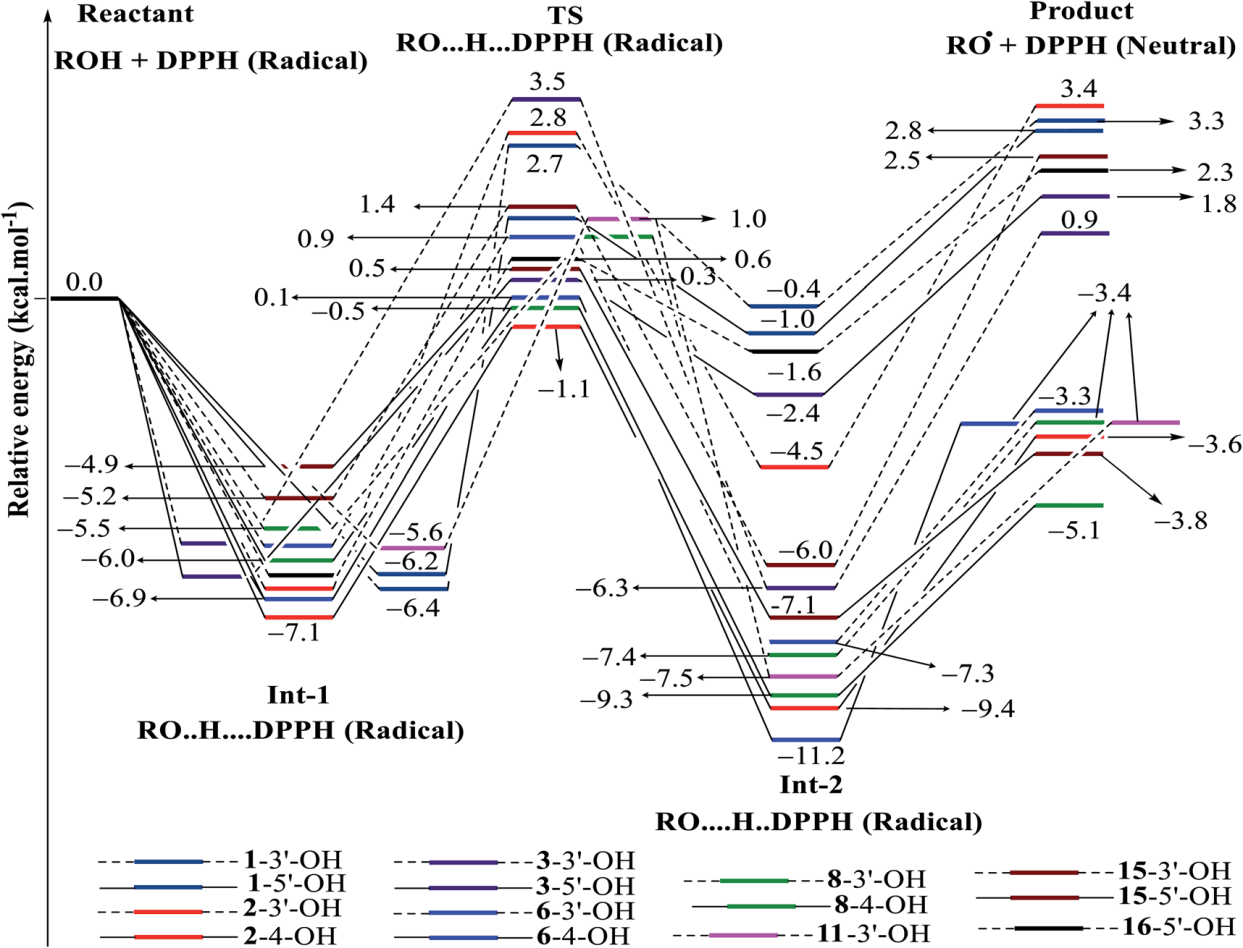
Energy diagram for the reaction of DPPH radicals attack to the studied compounds 1–3, 6, 8, 11, 15 and 16 at B3LYP/6-311G level of theory.

**Fig. 8:**
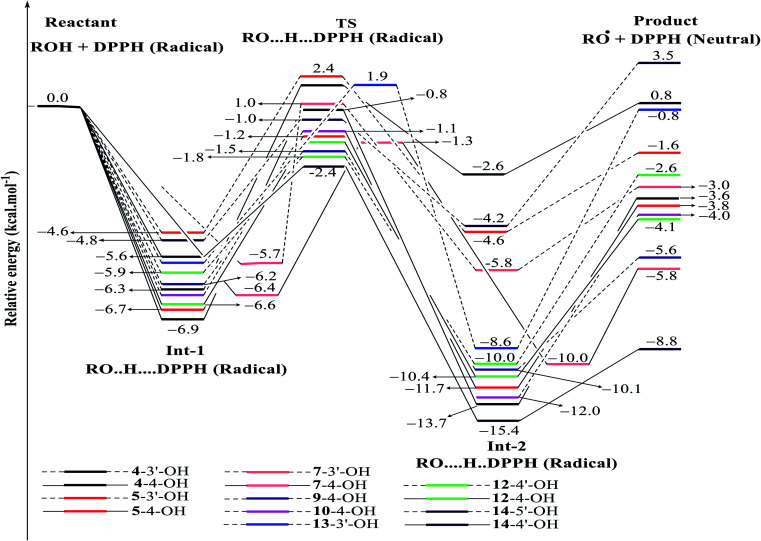
Energy diagram for the reaction of DPPH radicals attack to the studied compounds 4, 5, 7, 9, 10 and 12–14 at B3LYP/6-311G level of theory.

DPPH radicals are familiar agents in experiment laboratories that prove the efficacies of natural products in antioxidant targets.^33–39^ Our current account has employed this agent to discover the kinetic pathway for the radical scavenging treatment of a class of phenylbenzofurans 1–16 by using DFT calculations (Fig. 7, 8, S4† and Table 4). The first feature is easy to recognize, where the DPPH radical was able to isolate H atoms of OH groups in most studied compounds *via* an exothermic reaction (Δ*G* < 0), except for compounds 1, 3, 16, 2-3′-OH, 4-3′-OH and 14-5′-OH.

**Table:** In the gaseous phase and 298.15 K, the calculated ΔG^#^ and *k* at the B3LYP/6-311G level for DPPH radical attacks

Reaction	Δ*G* (kcal mol^−1^)	Δ*G*^#^ (kcal mol^−1^)	*k* (L mol^−1^ s)	Reaction	Δ*G* (kcal mol^−1^)	Δ*G*^#^ (kcal mol^−1^)	*k* (L mol^−1^ s)
1-3′-OH	3.07	10.7	1.004 × 10^8^	8-3′-OH	−3.67	7.8	2.192 × 10^9^
1-5′-OH	2.55	8.9	6.562 × 10^8^	8-4-OH	−5.24	7.5	2.939 × 10^9^
2-3′-OH	3.11	11.0	7.343 × 10^7^	9-4-OH	−5.77	6.0	1.432 × 10^10^
2-4-OH	−3.68	6.4	9.620 × 10^9^	10-4-OH	−3.98	6.3	1.061 × 10^10^
3-3′-OH	0.64	8.7	8.364 × 10^8^	11-3′-OH	−3.67	8.3	1.274 × 10^9^
3-5′-OH	1.44	11.1	6.602 × 10^7^	12-4-OH	−3.06	6.7	6.569 × 10^9^
4-4-OH	−3.70	7.9	1.820 × 10^9^	12-4′-OH	−3.06	4.8	5.330 × 10^10^
4-3′-OH	0.65	9.6	3.154 × 10^8^	13-3′-OH	−1.26	9.3	4.251 × 10^8^
5-4-OH	−3.88	6.7	7.183 × 10^9^	14-4′-OH	−9.51	4.7	5.666 × 10^10^
5-3′-OH	−1.76	9.7	2.867 × 10^8^	14-5′-OH	3.20	6.6	7.494 × 10^9^
6-4-OH	−3.32	8.3	1.248 × 10^9^	15-3′-OH	−2.85	8.8	7.149 × 10^8^
6-3′-OH	−3.64	9.6	3.283 × 10^8^	15-5′-OH	−3.97	8.3	1.325 × 10^9^
7-4-OH	−6.08	6.4	9.890 × 10^9^	16-5′-OH	2.14	8.5	9.626 × 10^8^
7-3′-OH	−3.11	9.1	5.419 × 10^8^				

In detail, the reaction between 14-4′-OH and DPPH was again found to have the lowest Δ*G* = −9.51 kcal mol^−1^ and the largest *k* = 5.666 × 10^10^ L mol^−1^ s (Table 4). The 14-4′-OH⋯N (DPPH) bond length of TS was 1.297 Å, and the relative energies of TS, Int-2 and Pr achieved minimum values of −2.4 kcal mol^−1^, −15.4 kcal mol^−1^ and −8.8 kcal mol^−1^, respectively (Fig. 8 and S4†), but 14-4′-OH and 12-4′-OH failed to do so.

The most striking feature arose from the considerable rate constant *k*. In agreement with the higher BDE enthalpies and the higher relative energies, coplanar compounds 1–3 and 16 mostly exhibited lower *k* values, thereby demonstrating that the interaction between the hydroxyl groups of coplanar phenylbenzofuran derivatives and the amine N(Ph)_3_ radical center of DPPH was not facilitated. The 3′-OH radical always exhibited higher BDE values than the 4-OH radical in compounds 2 and 4–8, which high Δ*G*^#^ values and low *k* values were assigned to the 3′-OH + DPPH radical and, in contrast, low Δ*G*^#^ values and high *k* values were assigned to the 4-OH + DPPH radical. In addition, several cases, such as the hydroxyl groups of 9, 10 and 12, yielded TSs with DPPH radicals with significant *k* values. Once again, this result reflected the good agreement between the mechanistic and kinetic studies, which indicated that 4′-OH and 4-OH seemed to be good sites for radical reactions rather than 3′-OH and 5′-OH.

We then highlighted the effect of methyl groups; the protons abstracted from 4-OH and 3′-OH of compounds 7, 8, 9 and 11 and transferred to the DPPH radical always produced TSs with better relative energies, Δ*G*^#^ and *k* than those of isolated compounds 5, 6, 10 and 13, respectively. Likewise, the rate constant of compound 8-3′-OH was 10 times higher than that of 7-3′-OH, and the Gibbs activation energy Δ*G*^#^ was 1.3 kcal mol^−1^ lower. Apparently, 5- and 6′-methylations play a critical role in this process. Among stemofurans 2 and 4–6, the 3′-OH forms of 4–6 exhibit equivalent values of Δ*G*^#^ = 9.6 kcal mol^−1^ and *k* = 3.101 × 10^10^ L mol^−1^ s, which are much better than those of compound 2 3′-OH (Δ*G*^#^ = 11.0 kcal mol^−1^ and *k* = 7.343 × 10^7^ L mol^−1^ s). These results suggest that mono-, di-, and tri-methylations are key factors for improving the antioxidant activity of 2-phenylbenzofurans. Finally, on the basis of additional evidence derived from three isolated compounds, 1, 3 and 15, 4′-methylation or 2′,4′,6′-trimethylations, was highly likely to be the main reason for the decrease in Δ*G* and Δ*G*^#^ values and the increase in *k* when comparing compound 1 and compound 3 or compound 15.

## 4. Conclusion

The antioxidant hypothesis of naturally occurring stilbenoid-type 2-phenylbenzofurans has successfully been investigated by density functional theory-based methods. The results indicated that the HAT pathway was preferentially closely related to the antioxidant action of these studied compounds in the gaseous state, but the SPLET model was the favorable mechanism in liquids, especially in terms of acetone. Numerous parameters, for instance, hydroxyl distribution, π-electron delocalization, potential polarizability, ionization potential (IP), proton affinity (PA), spin density, and especially BDE and PA values, provided supportive information to confirm that the radical scavenging processes favorably occurred *via* O–H bond breaking in rings A and B. Methylation at carbon C-5 of ring A and at carbons C-2′, C-4′ and C-6′ of ring B took contributed to decreasing the BDE values. The kinetic reactions between DPPH radicals and the studied compounds involve two intermediates and one transition state, in which attack by these radicals on most of the studied compounds was an exothermic reaction. Both mechanics and kinetics studies suggested that because of the lowest gaseous-phase BDE enthalpy of 71.66 kcal mol^−1^ and acetone-phase PA enthalpy of 33.68 kcal mol^−1^ and the lowest Δ*G*^#^ value of 4.7 kcal mol^−1^ and Δ*G* value of −9.51 kcal mol^−1^ but the largest rate constant *k* value of 5.666 × 10^10^ L mol^−1^ s, stemofuran U (14), with an unusual hydroxyl group at carbon C-4′, was a promising antioxidant agent. For the studied compounds, good antioxidant sites generally follow the order 4′-OH > 4-OH > 3′-OH and 5′-OH. This manuscript provides necessary guidelines for future research.

## Abbreviations

DFT: Density functional theory
TD-DFT: Time dependent density functional theory
HSAB: Hard and soft acids and bases
HOMO: Highest occupied molecular orbital
LUMO: Lowest unoccupied molecular orbital
BDE: Homolytic bond dissociation enthalpy
PDE: Heterolytic bond dissociation enthalpy
IP: Ionization potential
PA: Proton affinity
ETE: Electron transfer enthalpy
HAT: Hydrogen atom transfer
SET-PT: Single electron transfer-proton transfer
SPLET: Sequential proton loss electron transfer
Reactants: Res
Ints: Intermediates
TSs: Transition states
Products: Prs
DDPH: 1,1-Diphenyl-2-picryl hydrazine
NMR: Nuclear magnetic resonance

## Conflicts of interest

The authors declare that we do not have any conflict of interest.

## Supplementary Material

RA-010-C9RA10835A-s001
