# Supplementary material for: Antioxidation of 2-phenylbenzofuran derivatives: structural-electronic effects and mechanisms
Source: RSC Adv. 2020 Feb 11;10(11):6315–32. doi: 10.1039/c9ra10835a (PMC9049690; doi:10.1039/c9ra10835a)
Supplement: RA-010-C9RA10835A-s001 [file RA-010-C9RA10835A-s001.pdf]

Electronic Supplementary Material (ESI) for RSC Advances.
This journal is © The Royal Society of Chemistry 2020

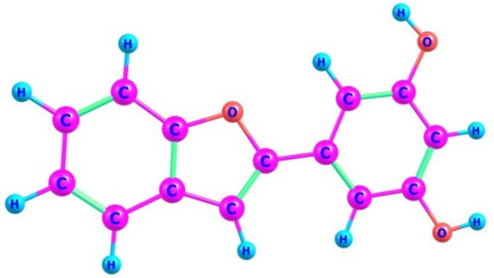	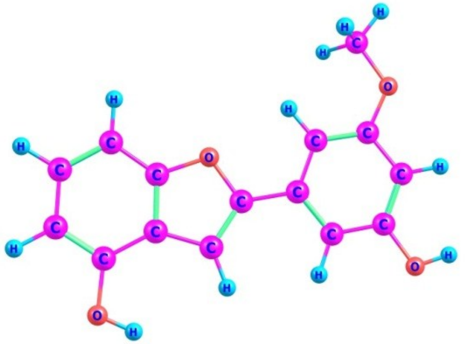	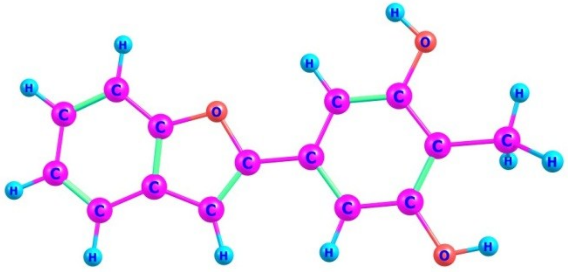
Compound **1**	Compound **2**	Compound **3**
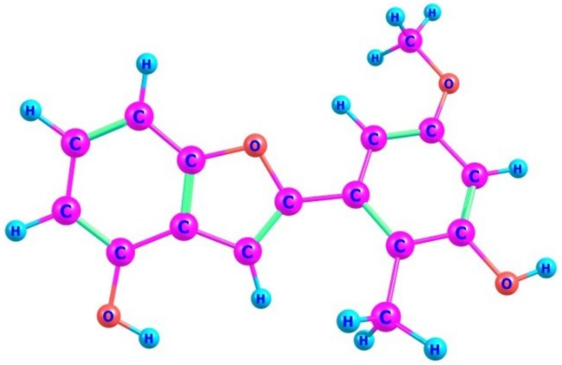	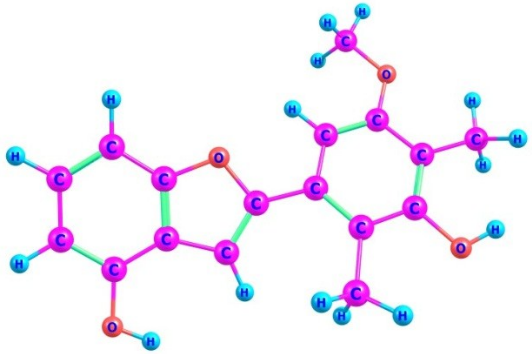	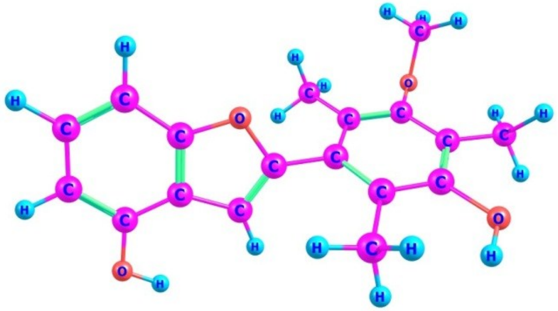
Compound **4**	Compound **5**	Compound **6**

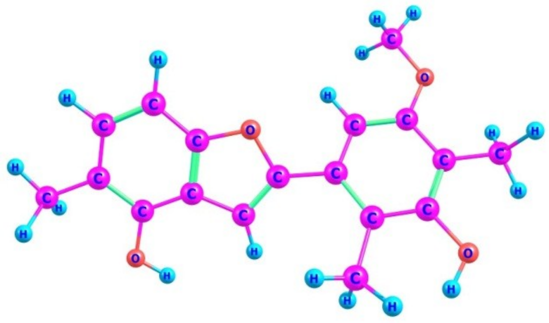	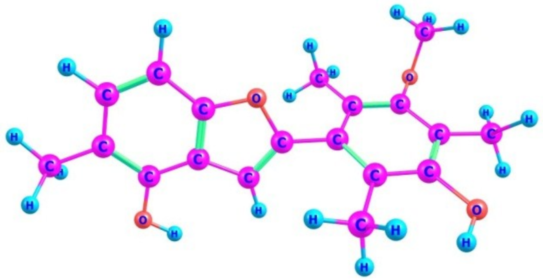	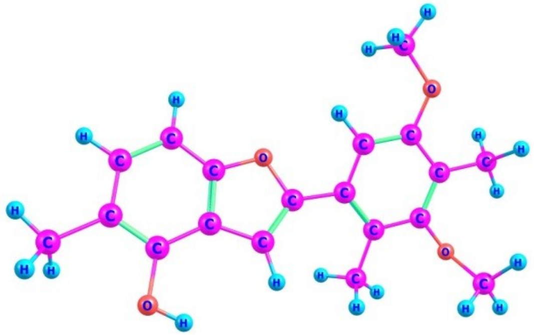
Compound **7**	Compound **8**	Compound **9**
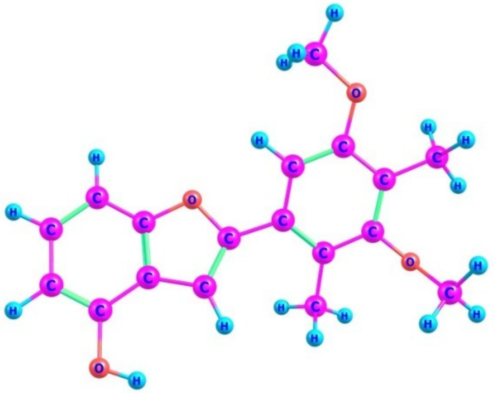	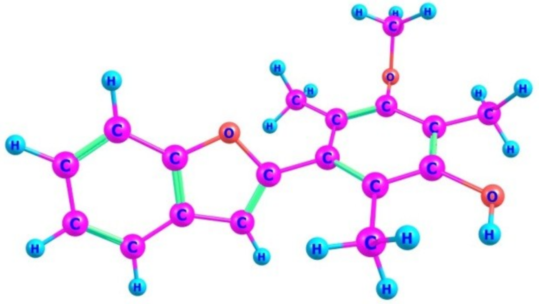	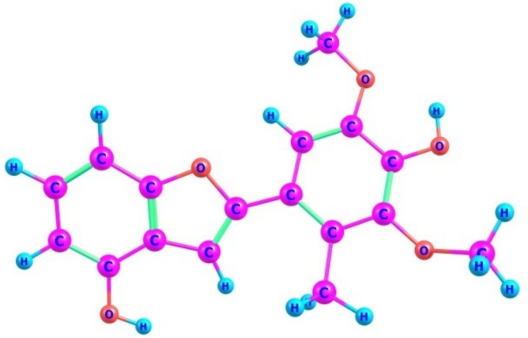
Compound **10**	Compound **11**	Compound **12**

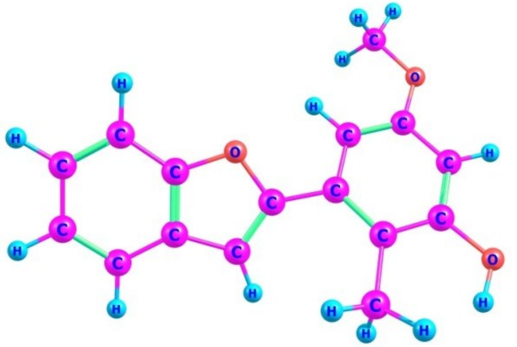

Compound **13**

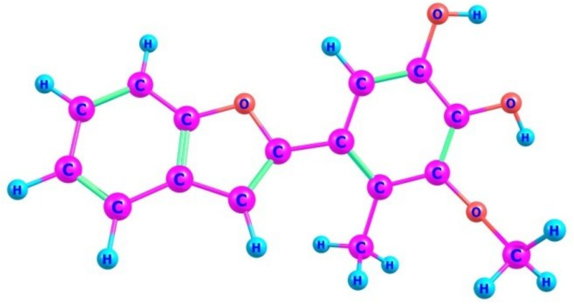

Compound **14**

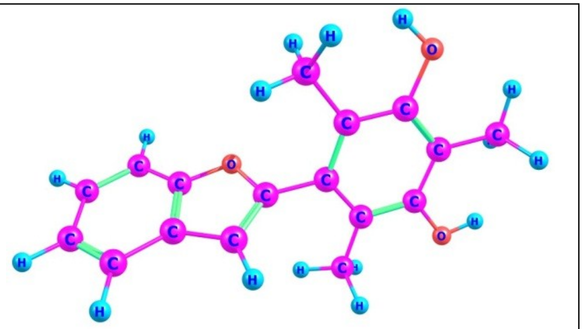

Compound **15**

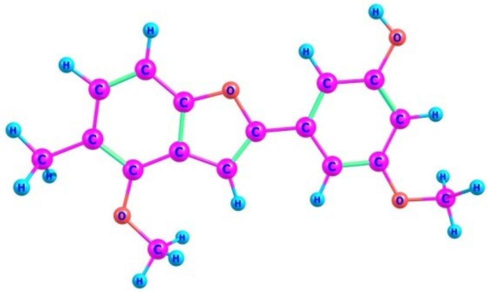

Compound **16**

Fig. S1. The state forms of the structures **1-16** in all studied mediums at B3LYP/6-311++G(d,p) level of theory

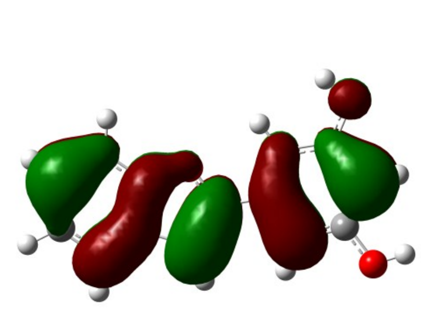	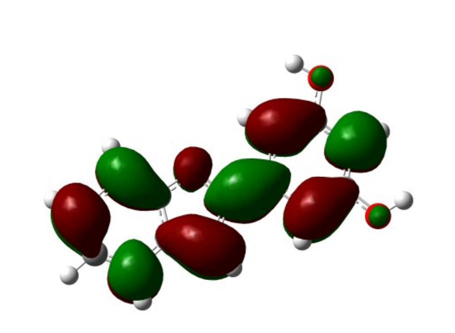	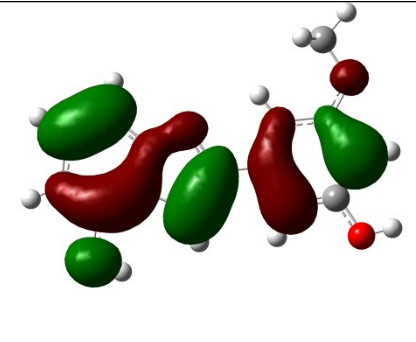	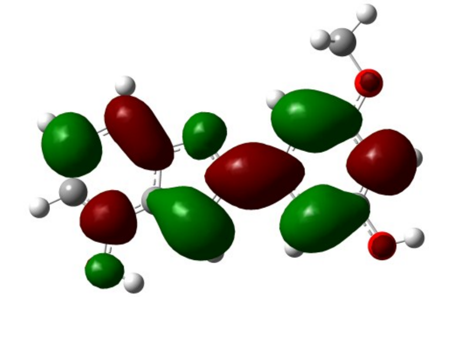
**1**-HOMO	**1**-LUMO	**2**-HOMO	**2**-LUMO
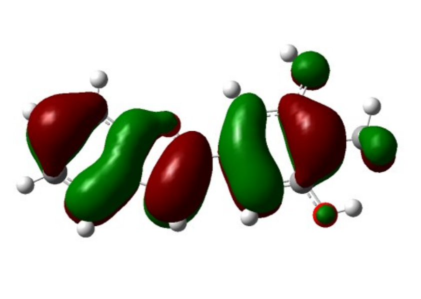	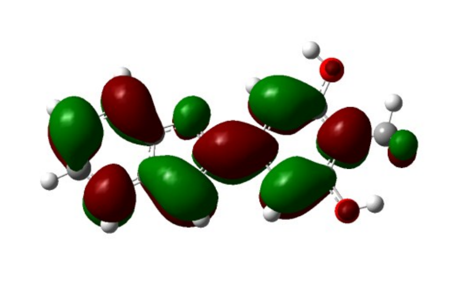	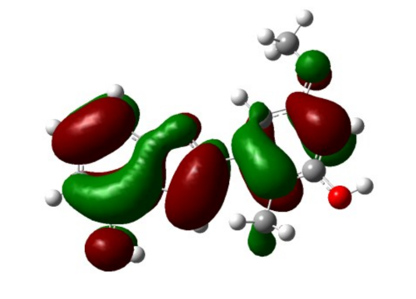	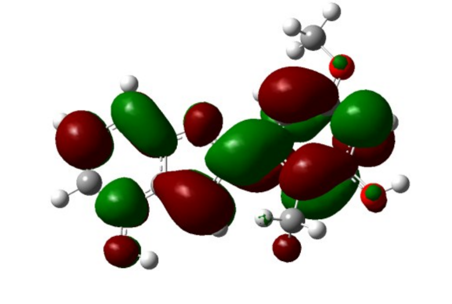
**3**-HOMO	**3**-LUMO	**4**-HOMO	**4**-LUMO
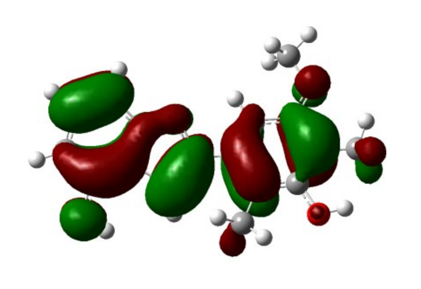	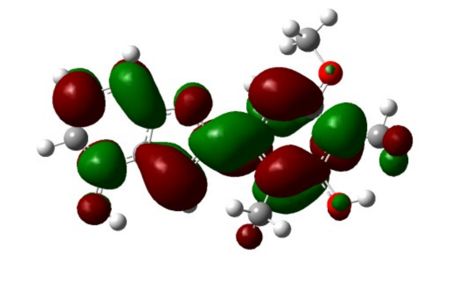	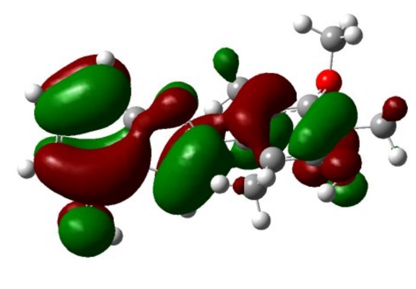	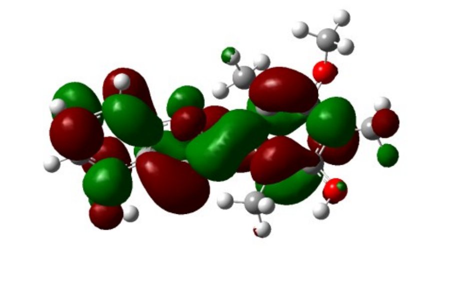

**5**-HOMO	**5**-LUMO	**6**-HOMO	**6**-LUMO
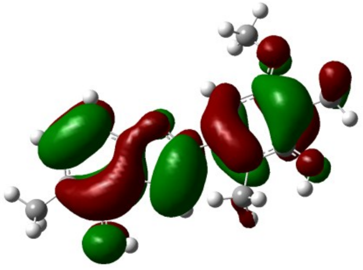	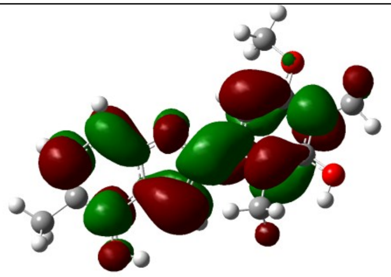	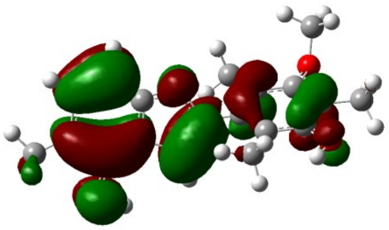	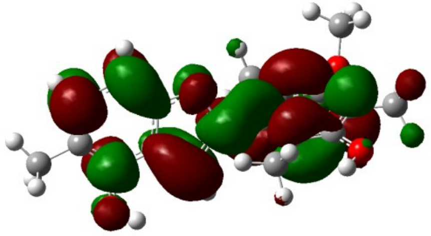
**7**-HOMO	**7**-LUMO	**8**-HOMO	**8**-LUMO
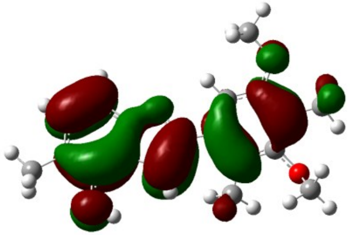	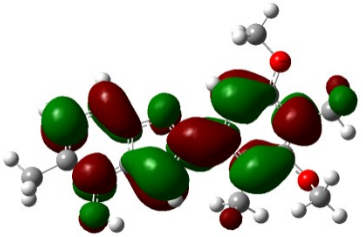	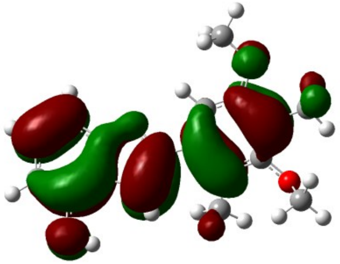	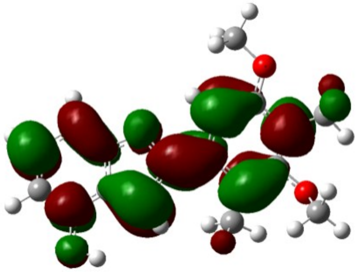
**9**-HOMO	**9**-LUMO	**10**-HOMO	**10**-LUMO
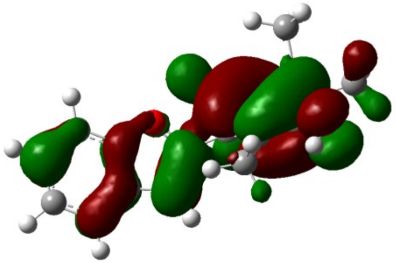	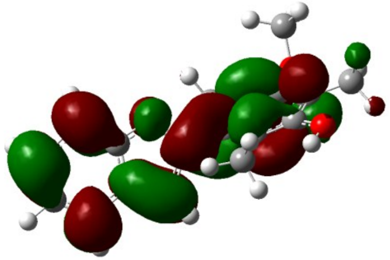	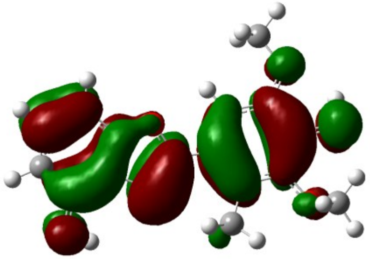	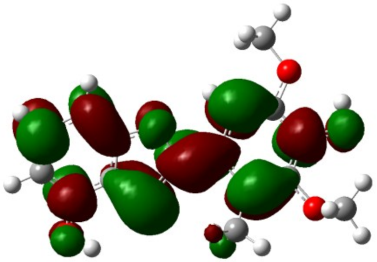

**11**-HOMO	**11**-LUMO	**12**-HOMO	**12**-LUMO
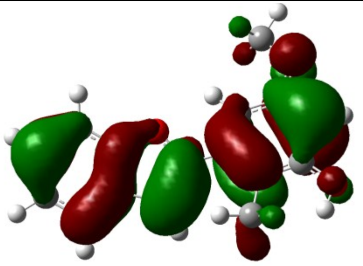	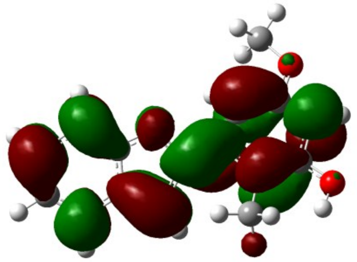	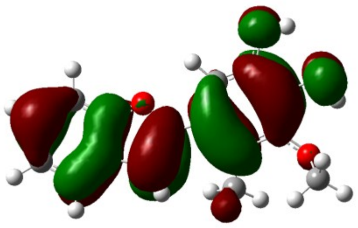	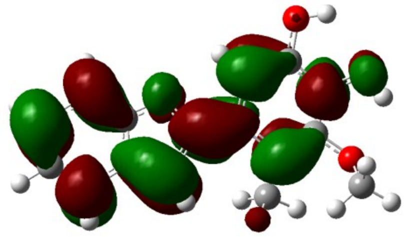
**13**-HOMO	**13**-LUMO	**14**-HOMO	**14**-LUMO
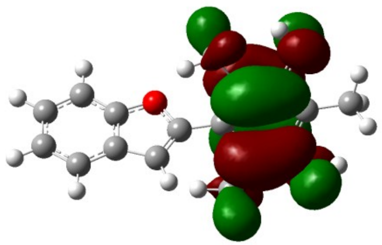	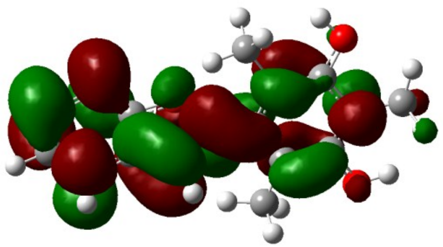	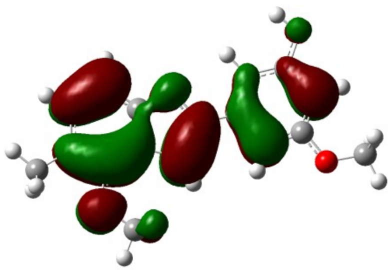	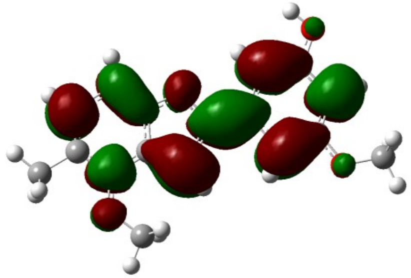
**15**-HOMO	**15**-LUMO	**16**-HOMO	**16**-LUMO

Fig. S2. HOMO and LUMO images of the neutral structures **1-16** in all studied mediums at B3LYP/6-311++G(d,p) level of theory

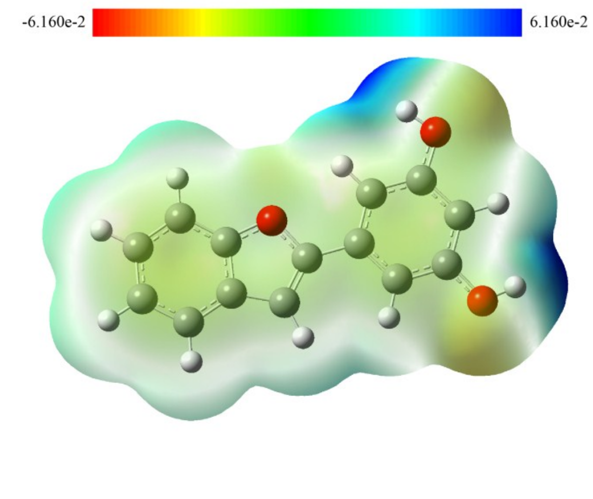

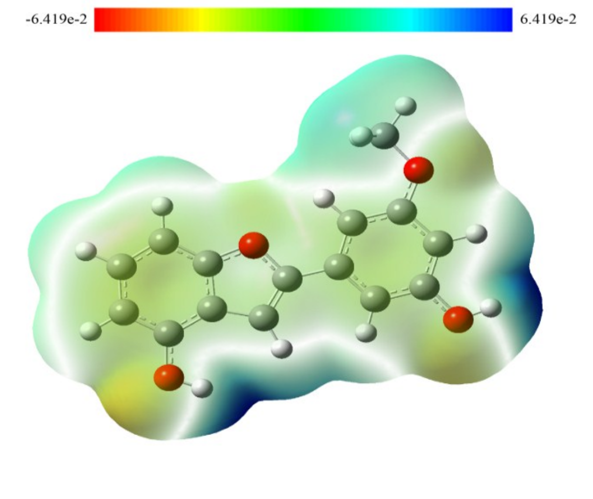

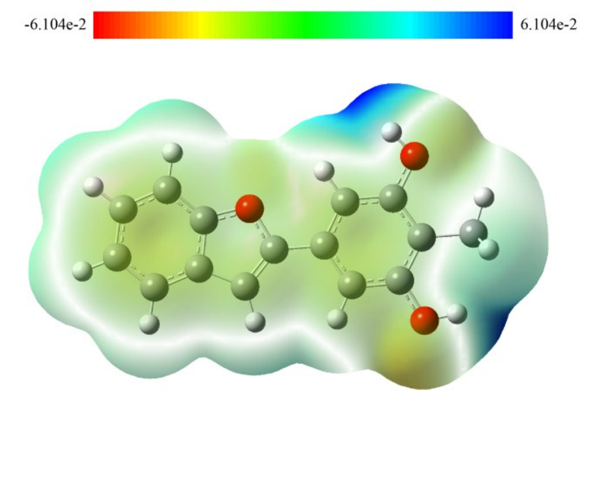

Compound **1**	Compound **2**	Compound **3**

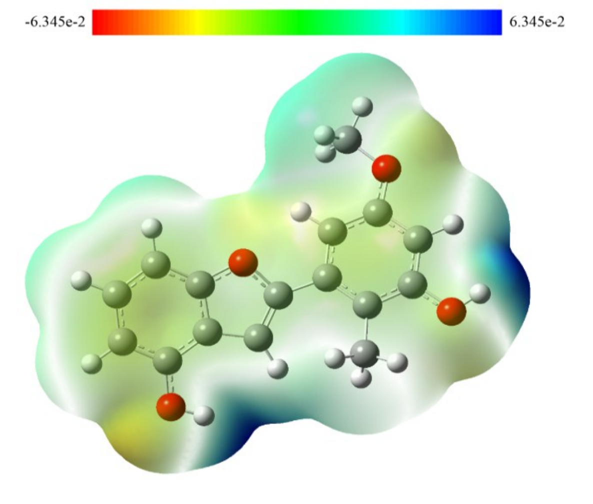

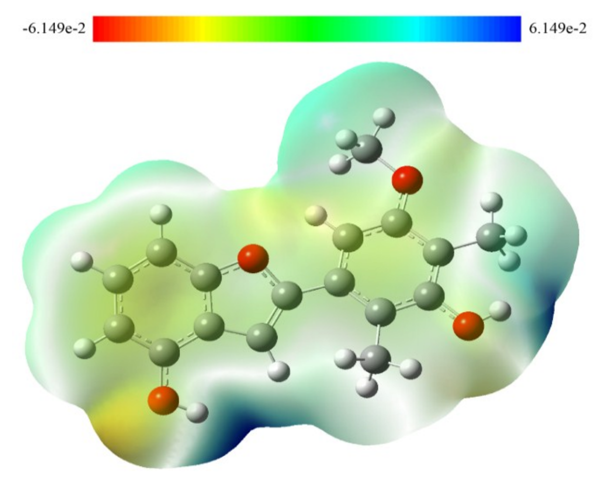

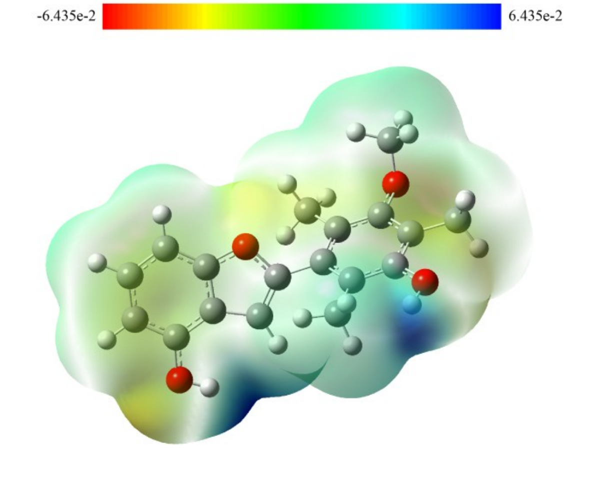

Compound **4**	Compound **5**	Compound **6**
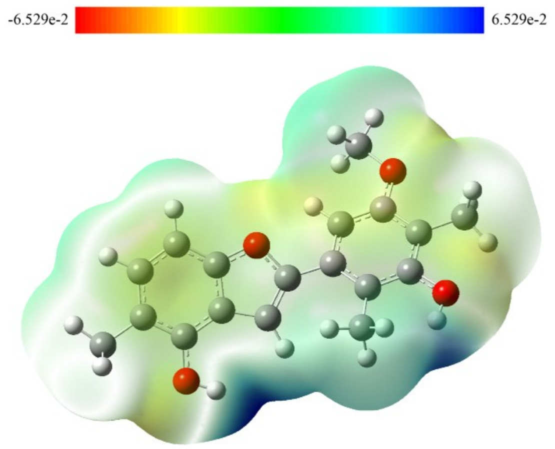	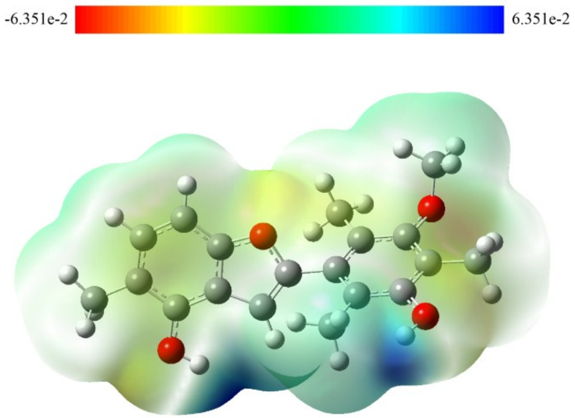	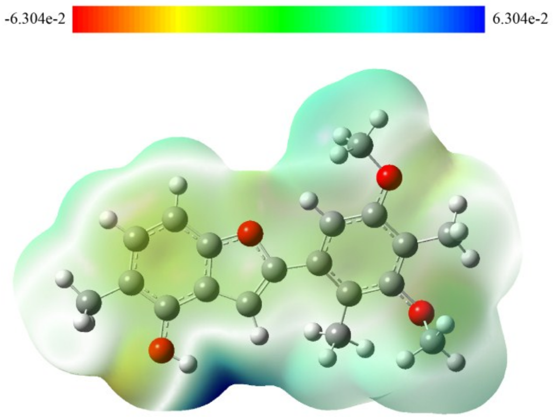
Compound **7**	Compound **8**	Compound **9**
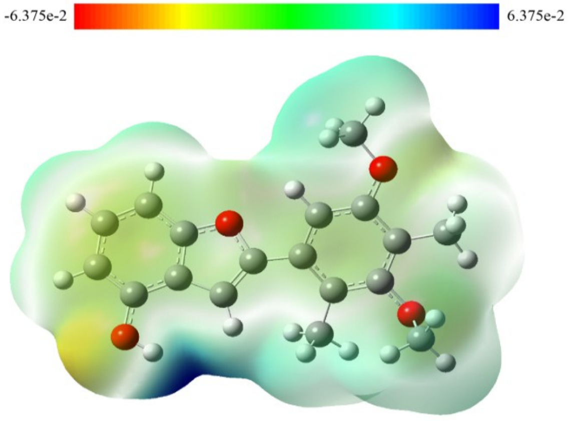	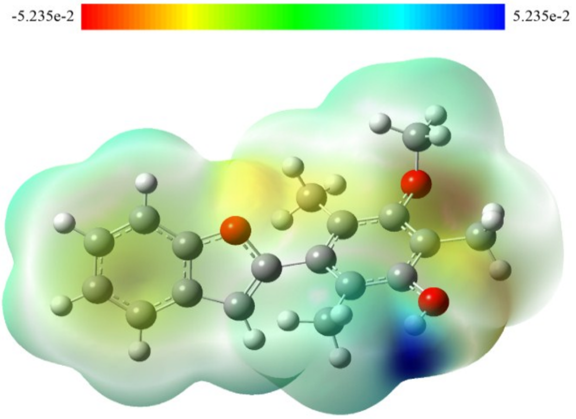	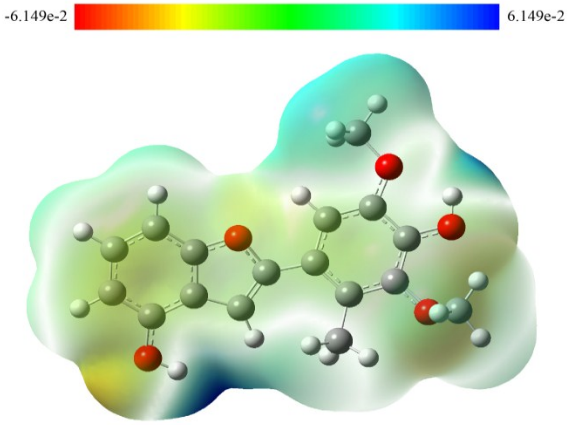
Compound **10**	Compound **11**	Compound **12**

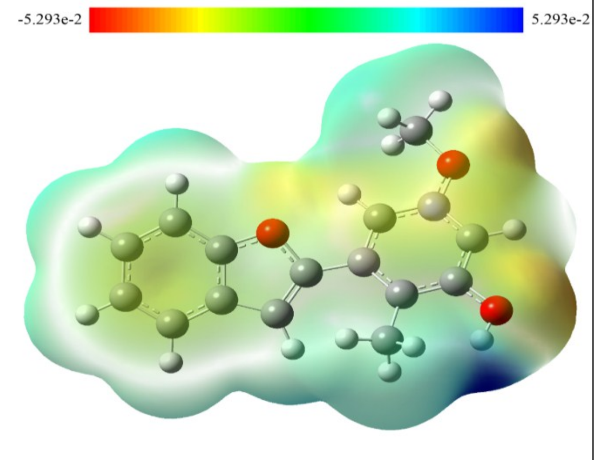

Compound **13**

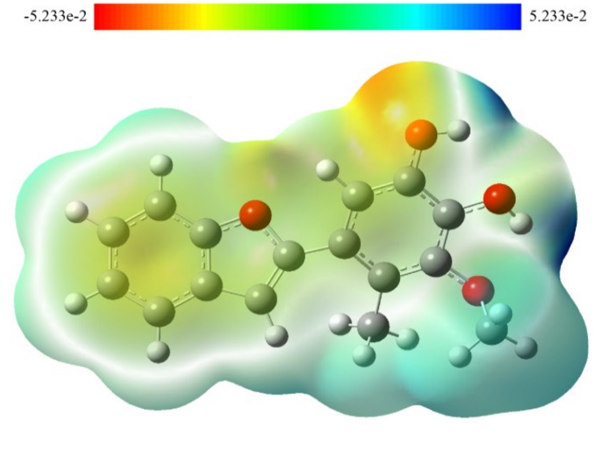

Compound **14**

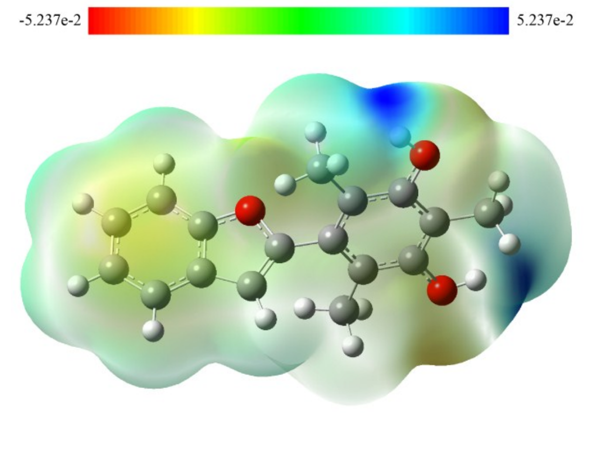

Compound **15**

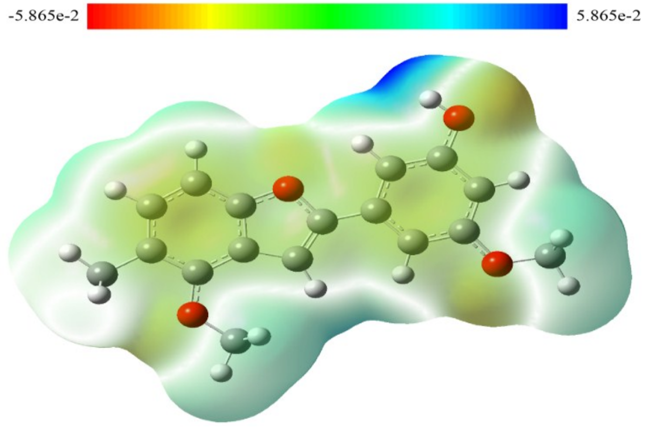

Compound **16**

Fig. S3. Molecular electrostatic potential mapped on the isodensity surface for the neutral structures **1-16** at B3LYP/6-311++G(d,p) level of theory in gaseous phase

1-3’ -OH (Int-1)

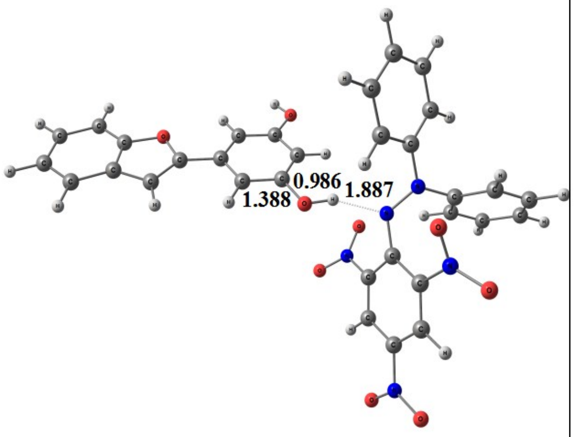

1-3’ -OH (TS)

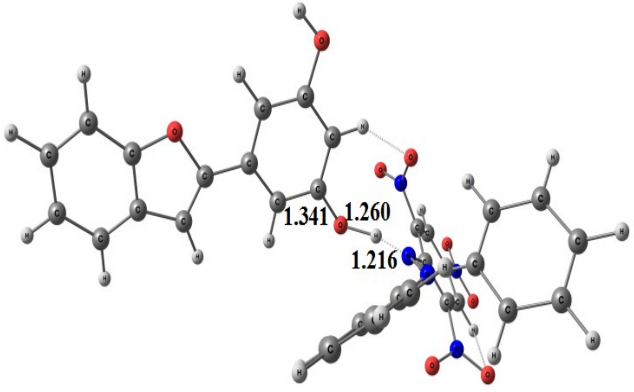

1-3’ -OH (Int-2)

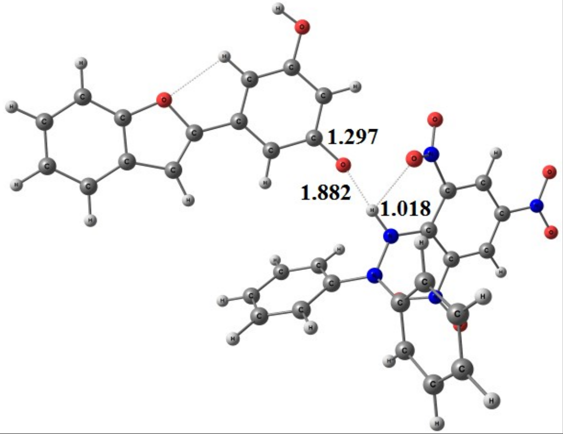

1-5’ -OH (Int-1)

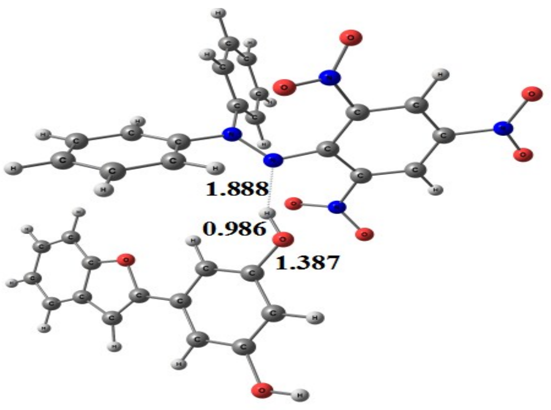

1-5’ -OH (TS)

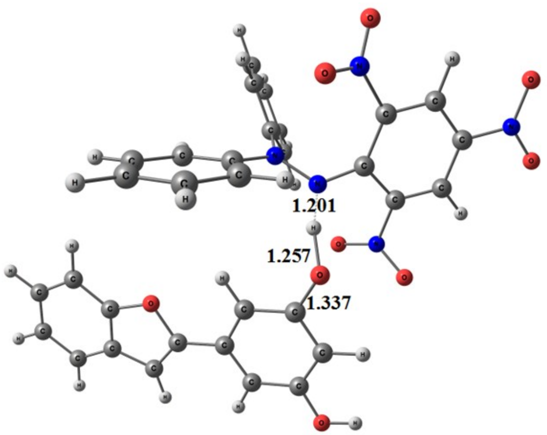

1-5’ -OH (Int-2)

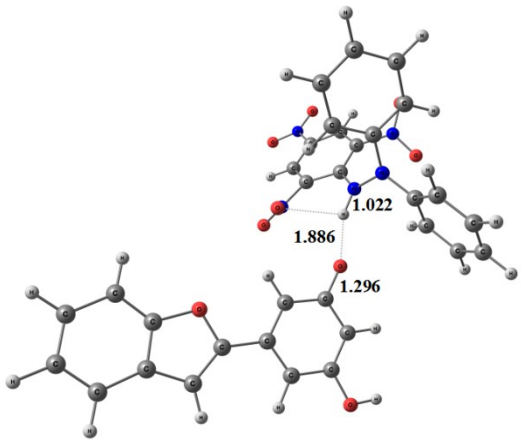

2-3’ -OH (Int-1)

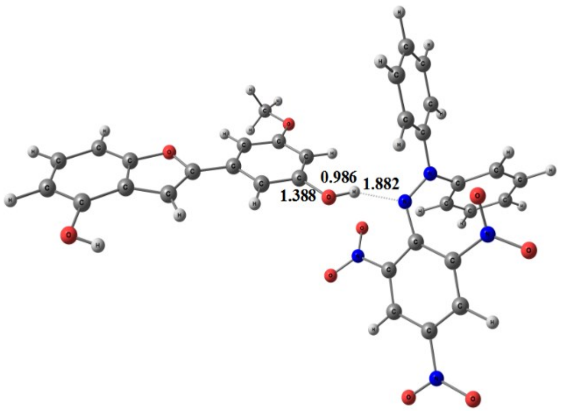

2-3’ -OH (TS)

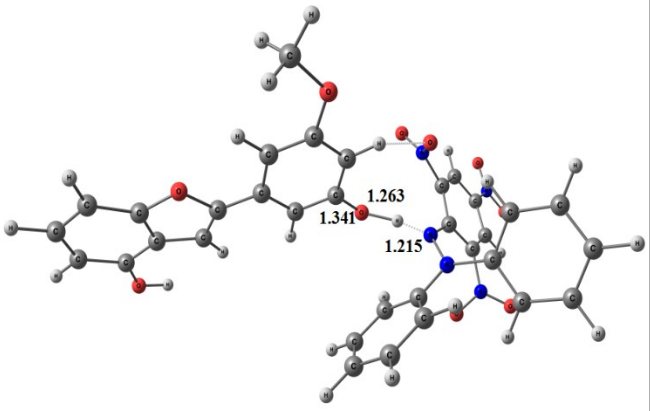

2-3’ -OH (Int-2)

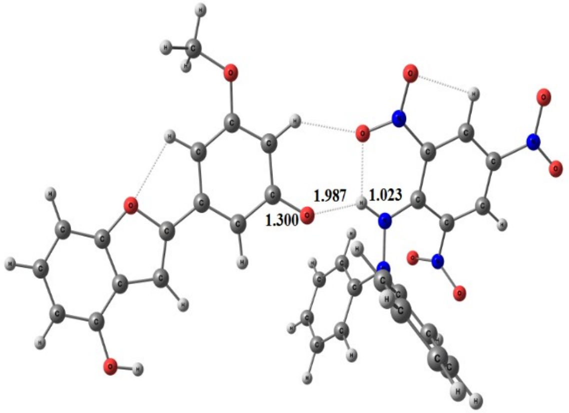

2-4-OH (Int-1)

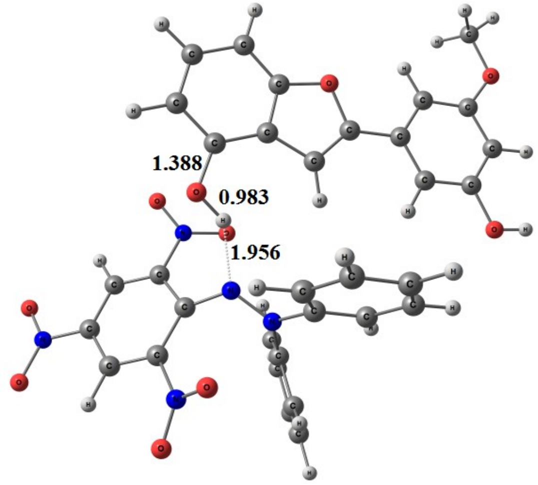

2-4-OH (TS)

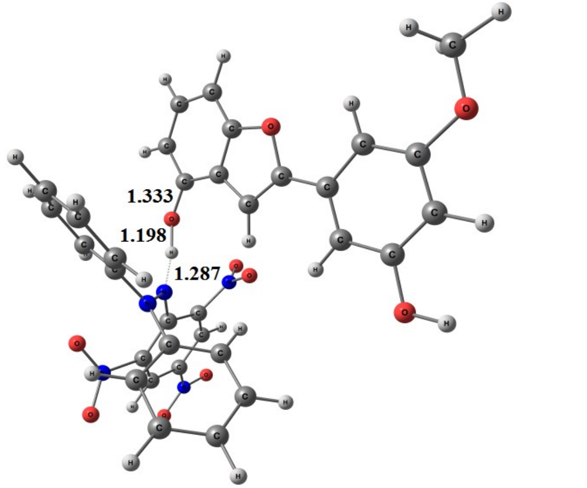

2-4-OH (Int-2)

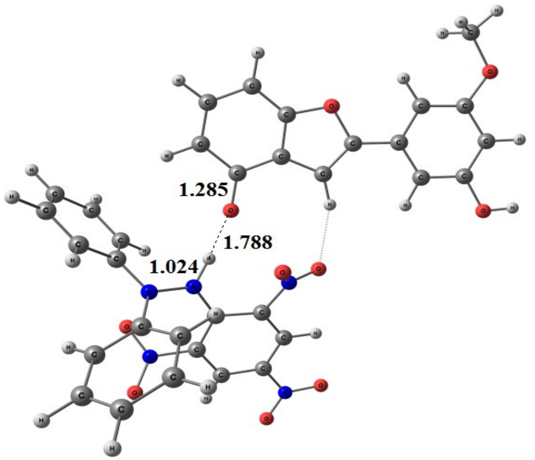

3-3’ -OH (Int-1)

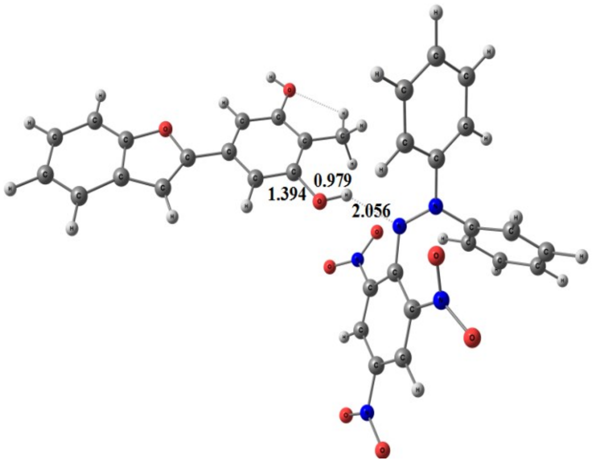

3-3’ -OH (TS)

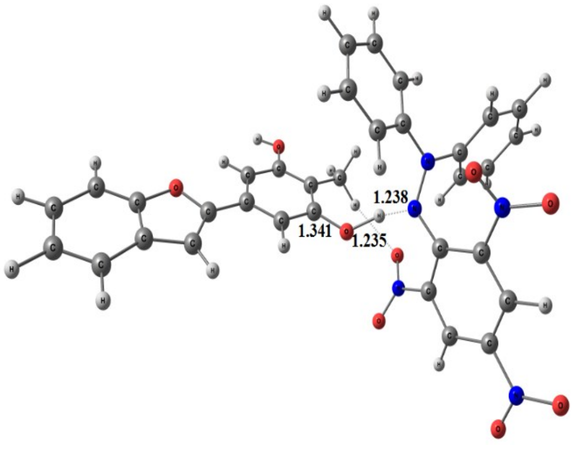

3-5’ -OH (Int-2)

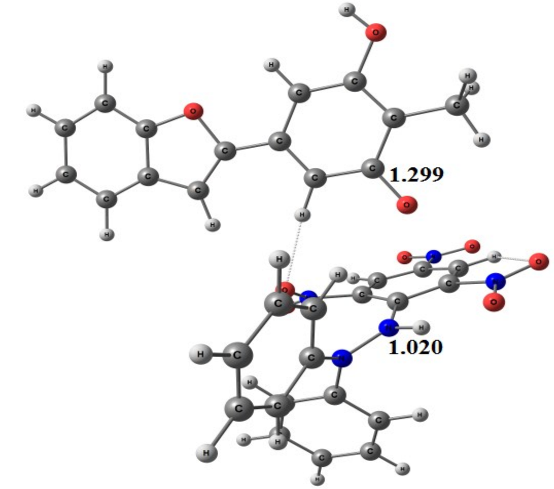

3-5’ -OH (Int-1)

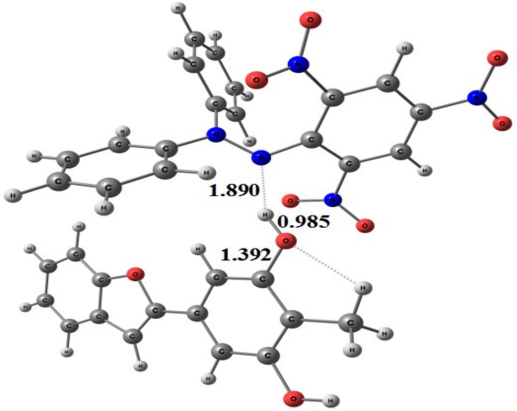

3-5’ -OH (TS)

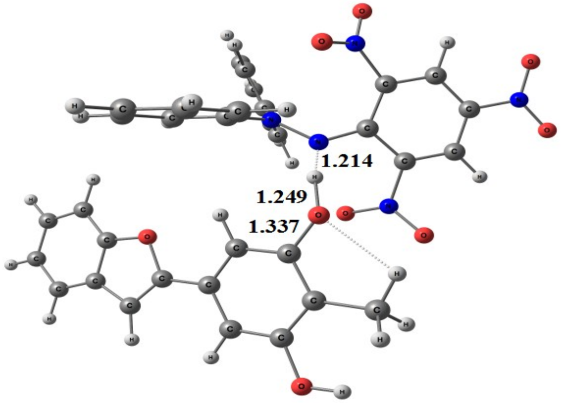

3-5’ -OH (Int-2)

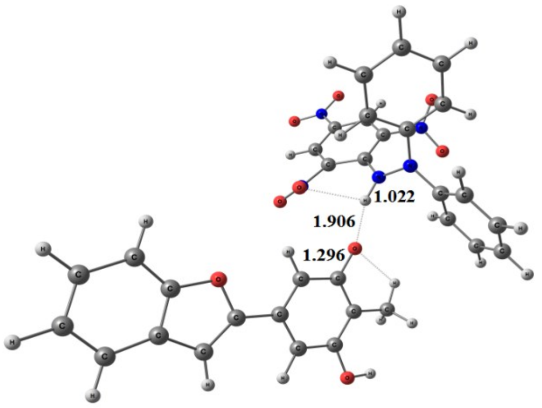

4-3’ -OH (Int-1)

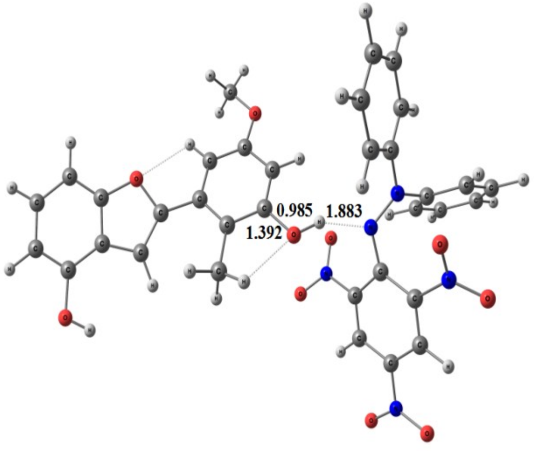

4-3’ -OH (TS)

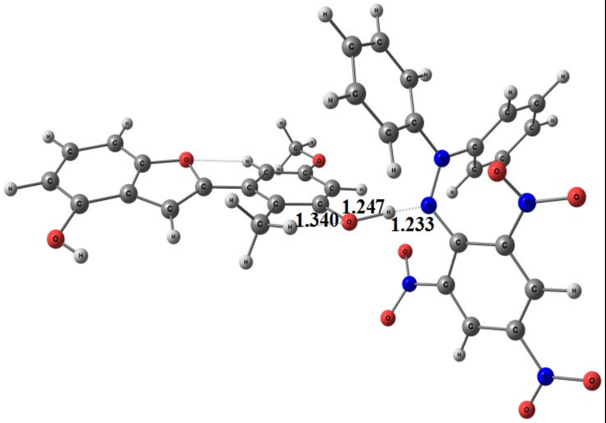

4-3’ -OH (Int-2)

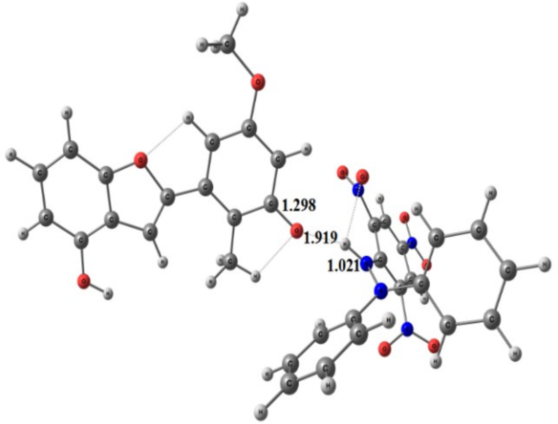

4-4-OH (Int-1)

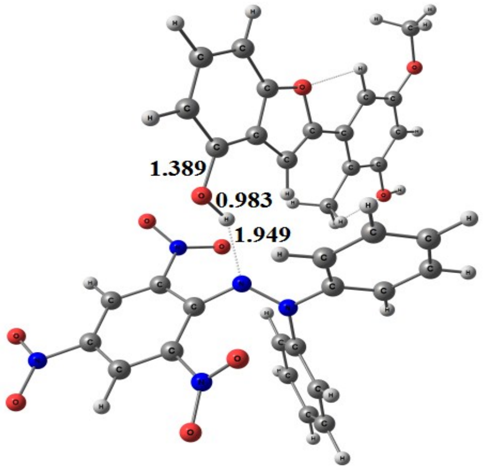

4-4-OH (TS)

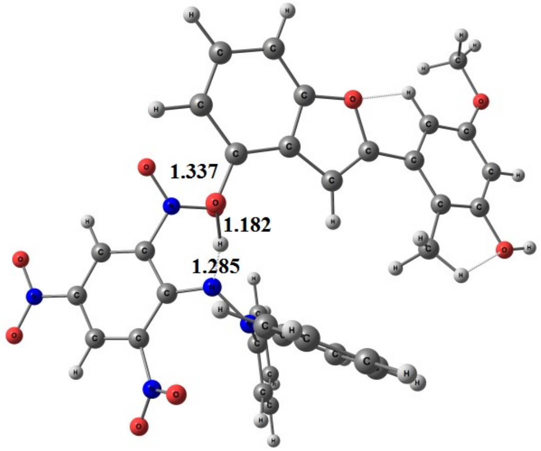

4-4-OH (Int-2)

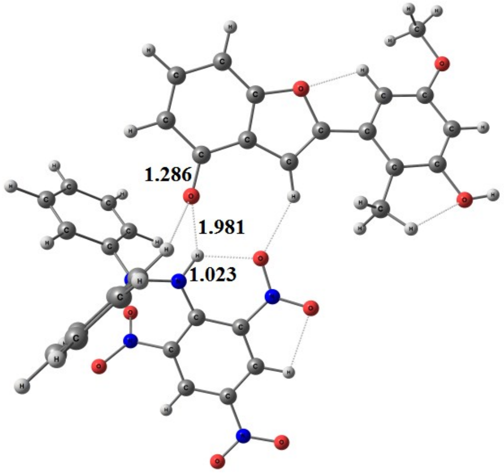

5-3’ -OH (Int-1)	5-3’ -OH (TS)	5-3’ -OH (Int-2)
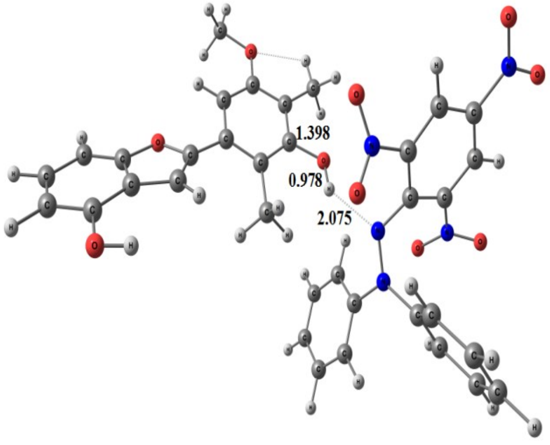 	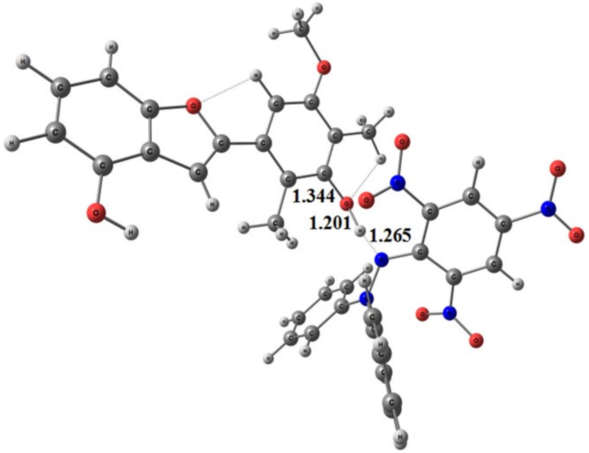 	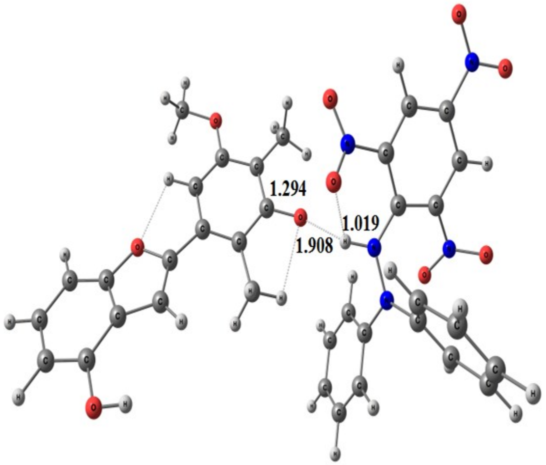
**5-**4-OH (Int-1)	**5-**4-OH (TS)	**5-**4-OH (Int-2)
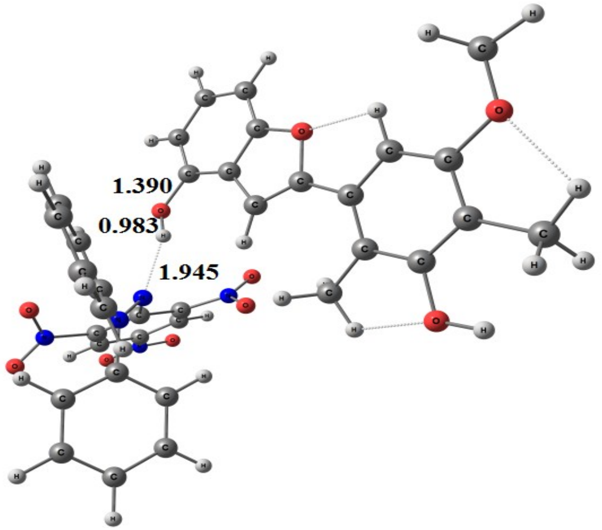 	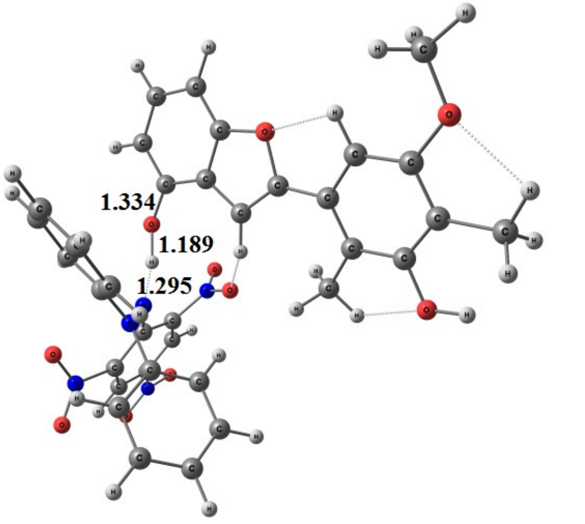 	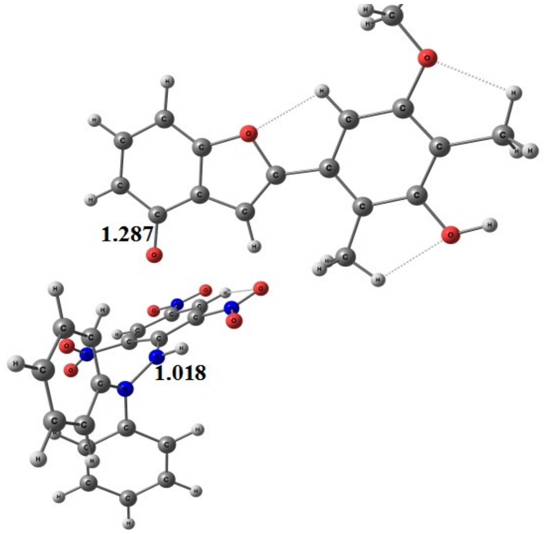

6-4-OH (Int-1)

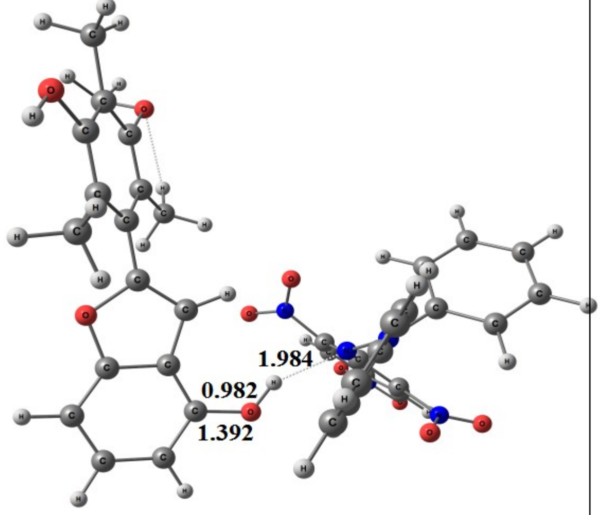

6-4-OH (TS)

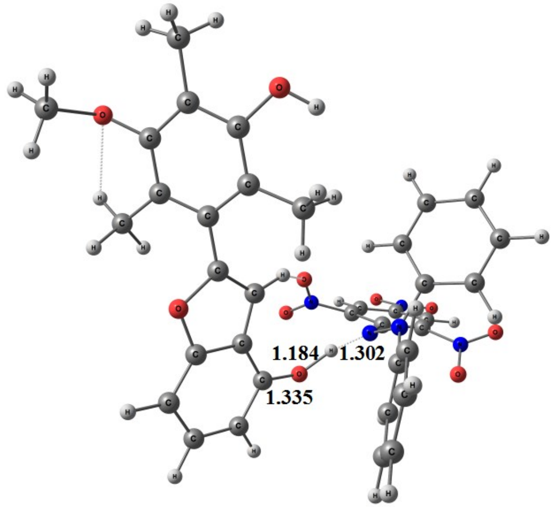

6-4-OH (Int-2)

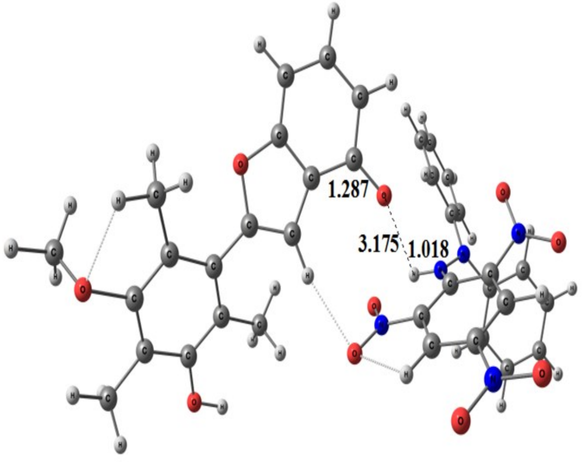

6-3’-OH (Int-1)

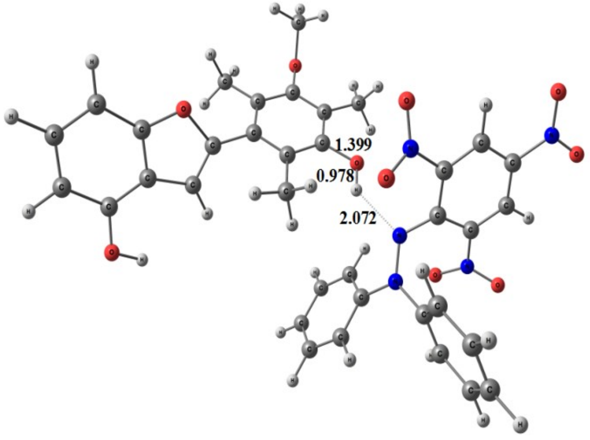

6-3’-OH (TS)

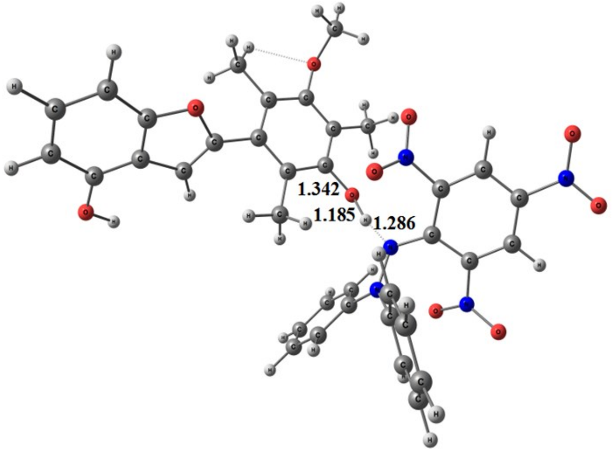

6-3’-OH (Int-2)

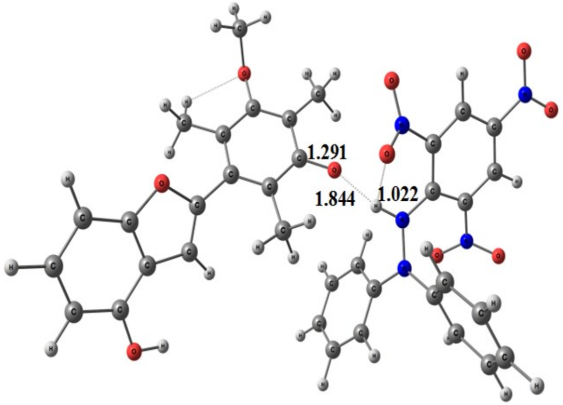

7-4-OH (Int-1)

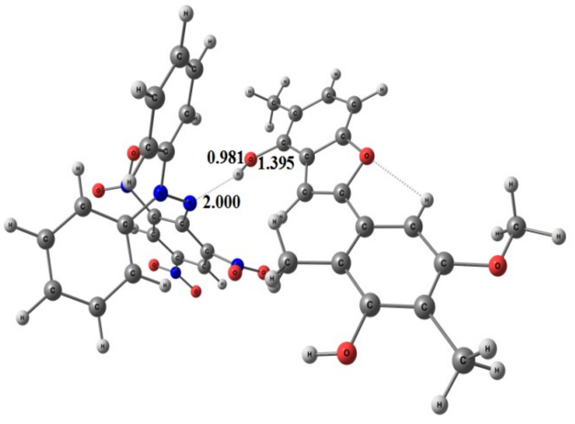

7-4-OH (TS)

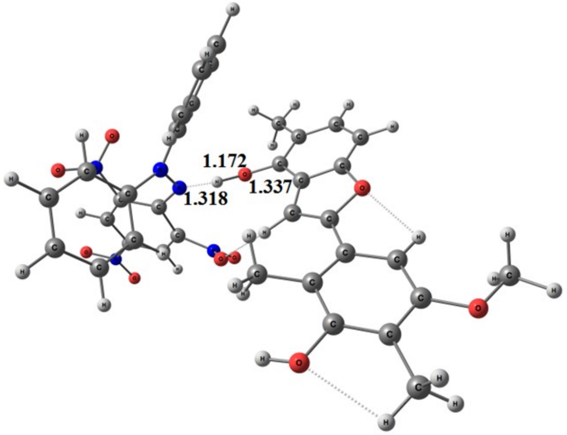

7-4-OH (Int-2)

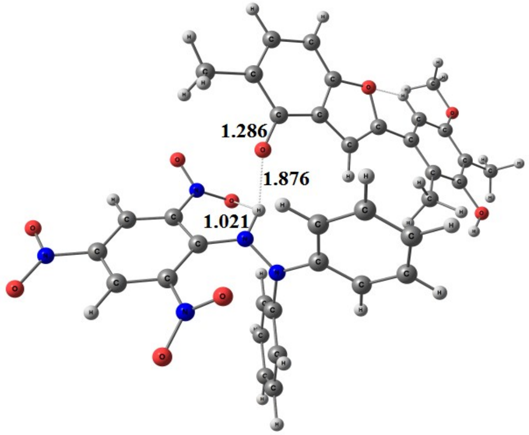

7-3’-OH (Int-1)

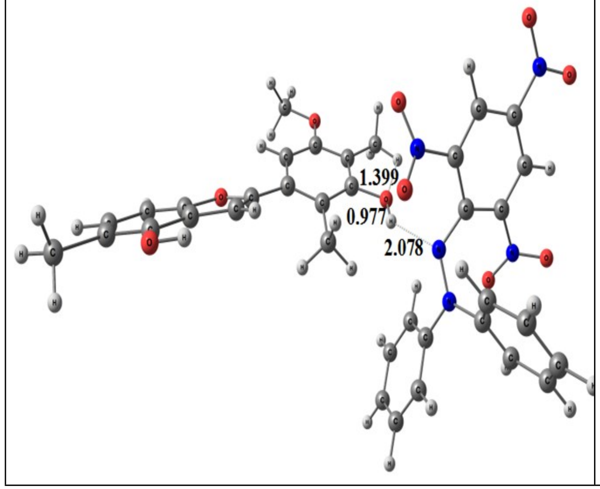

7-3’-OH (TS)

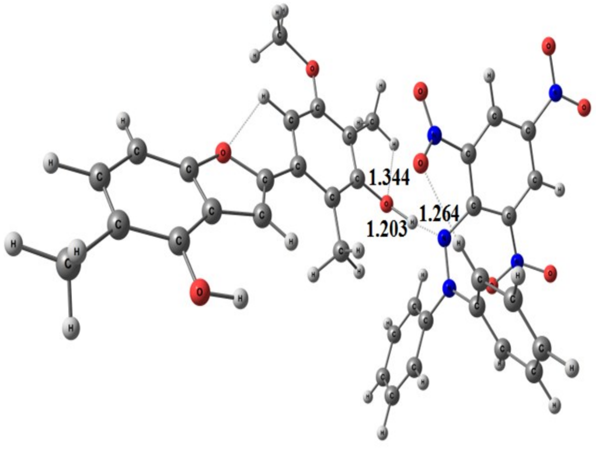

7-3’-OH (Int-2)

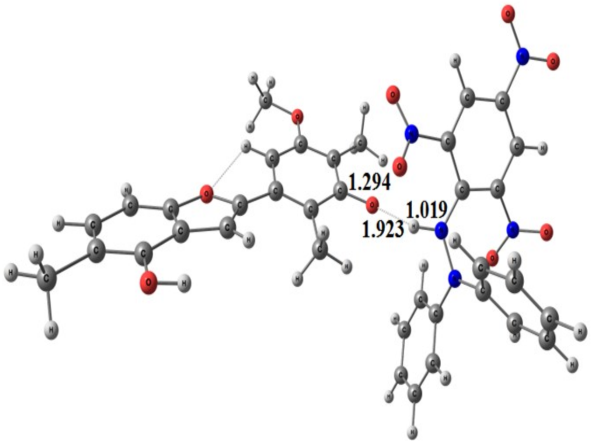

8-4-OH (Int-1)

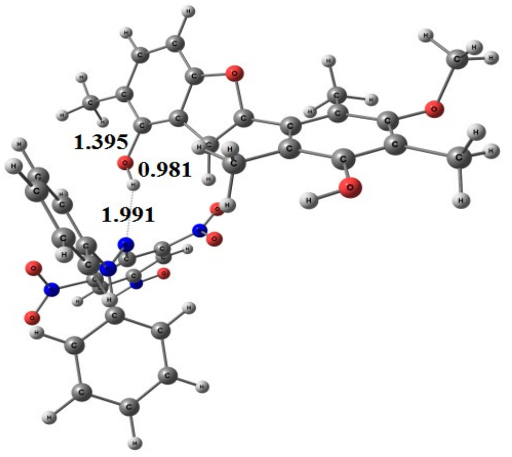

8-4-OH (TS)

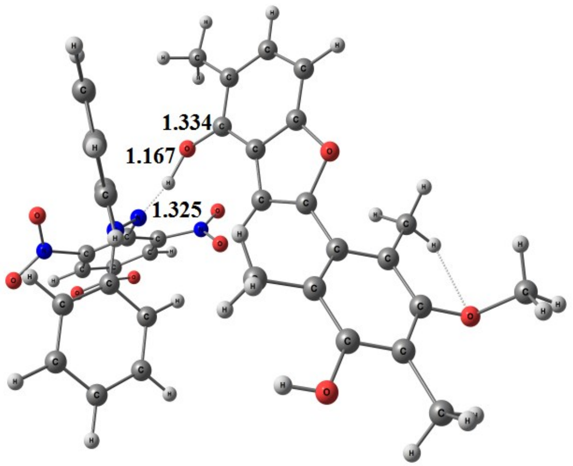

8-4-OH (Int-2)

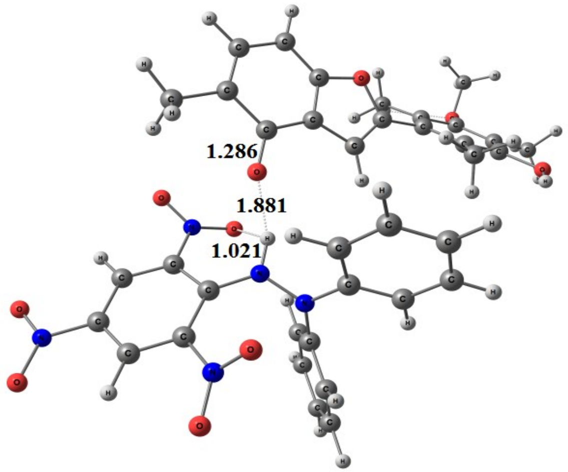

8-3’ -OH (Int-1)

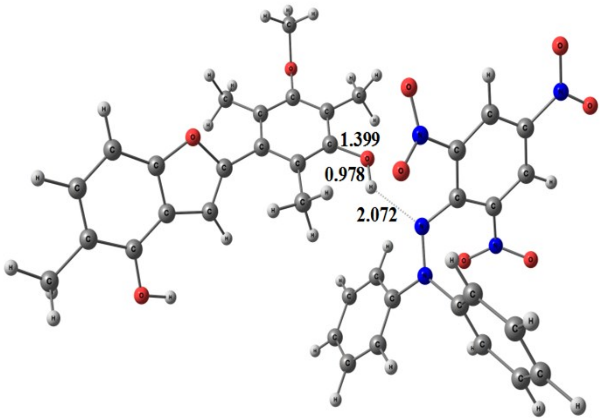

8-3’ -OH (TS)

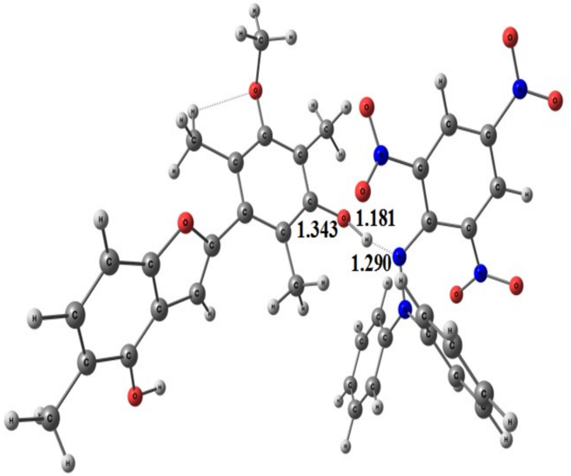

8-3’ -OH (Int-2)

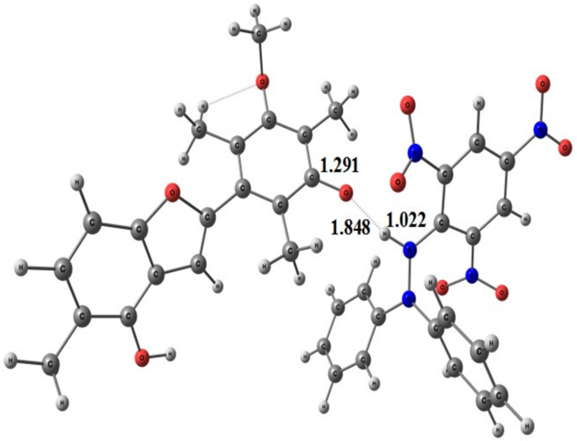

9-4-OH (Int-1)

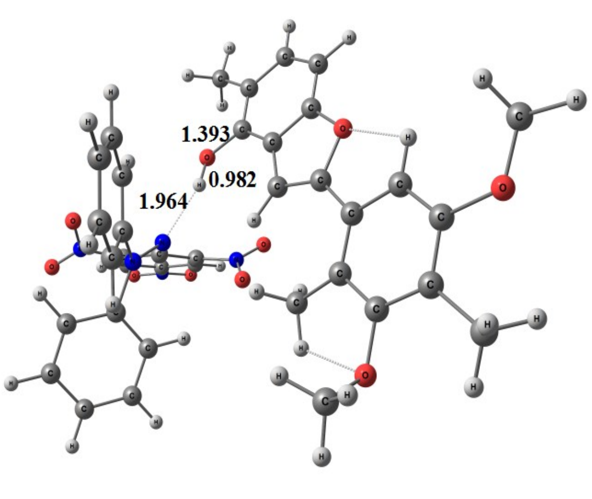

9-4-OH (TS)

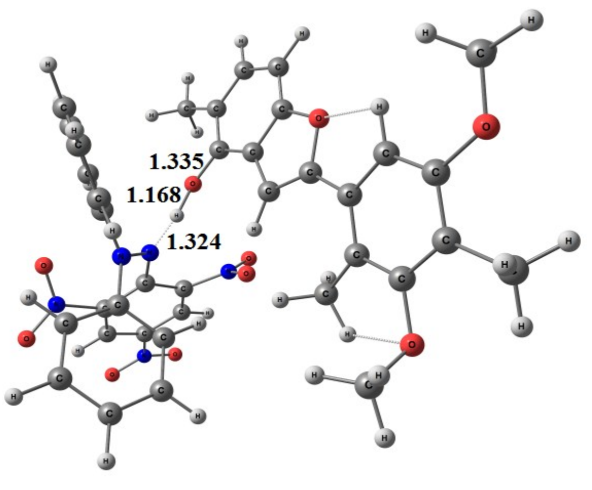

9-4-OH (Int-2)

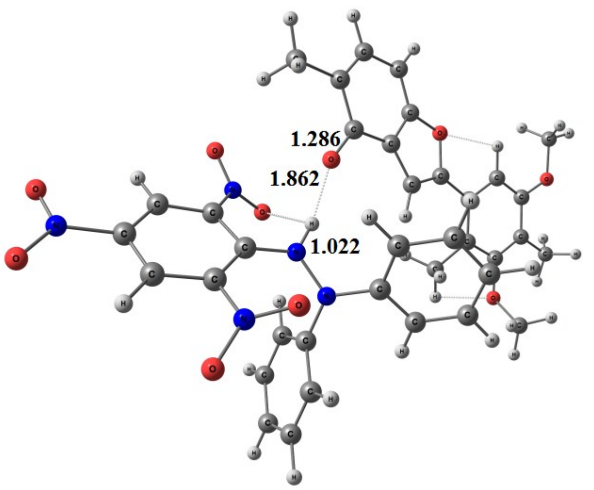

10-4-OH (Int-1)

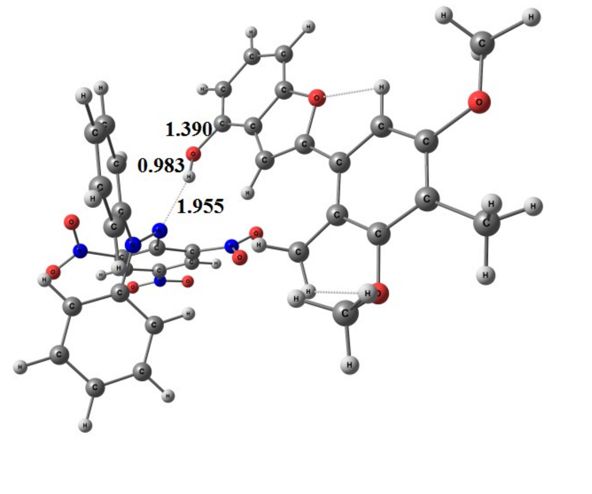

10-4-OH (TS)

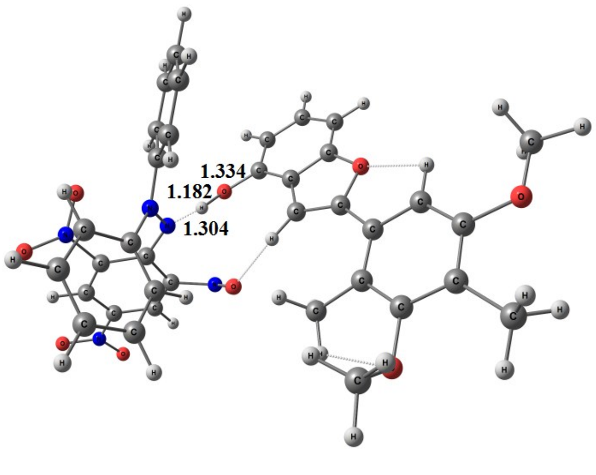

10-4-OH (Int-2)

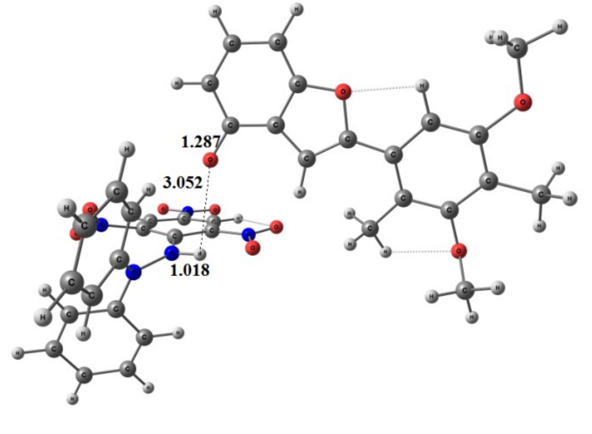

11-3’ -OH (Int-1)

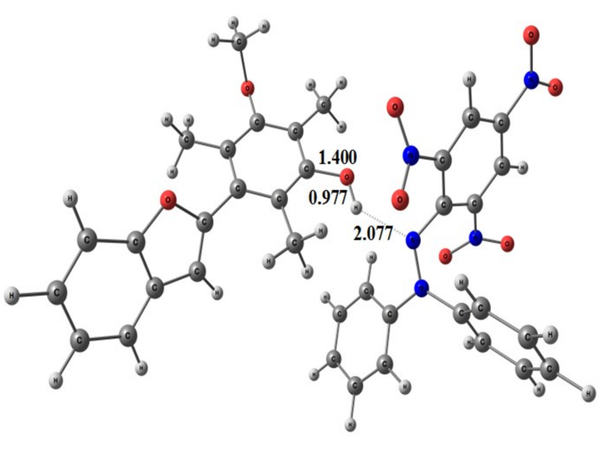

11-3’ -OH (TS)

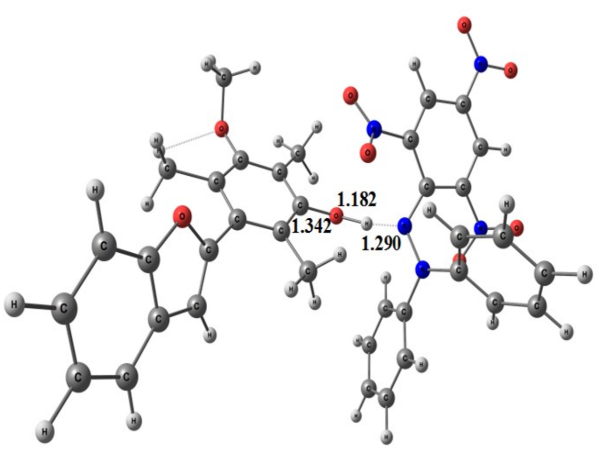

11-3’ -OH (Int-2)

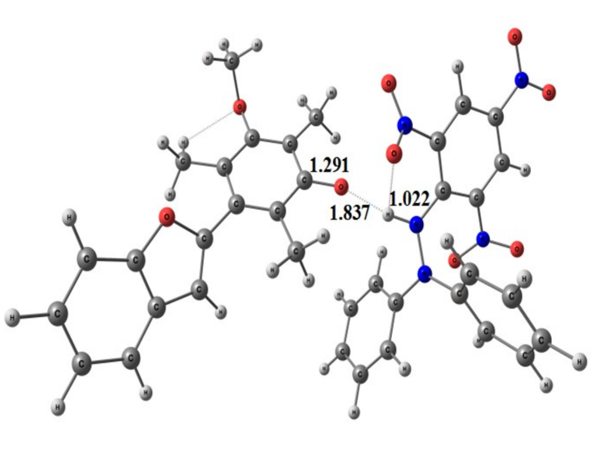

12-4-OH (Int-1)

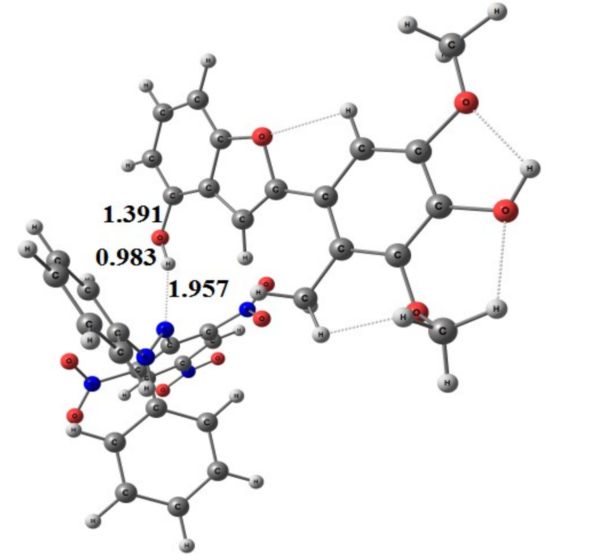

12-4-OH (TS)

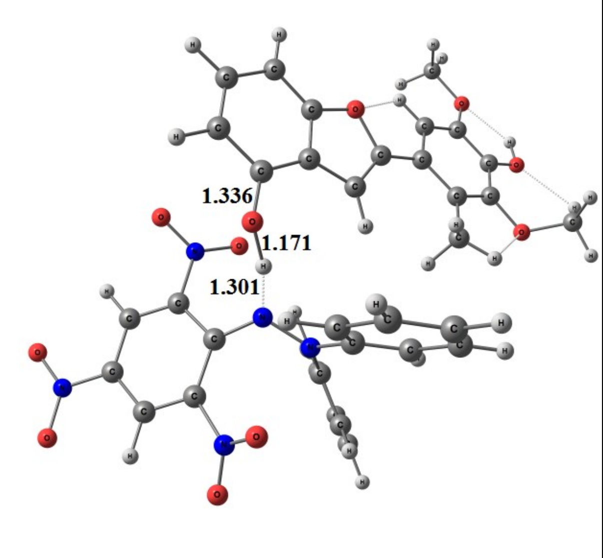

12-4-OH (Int-2)

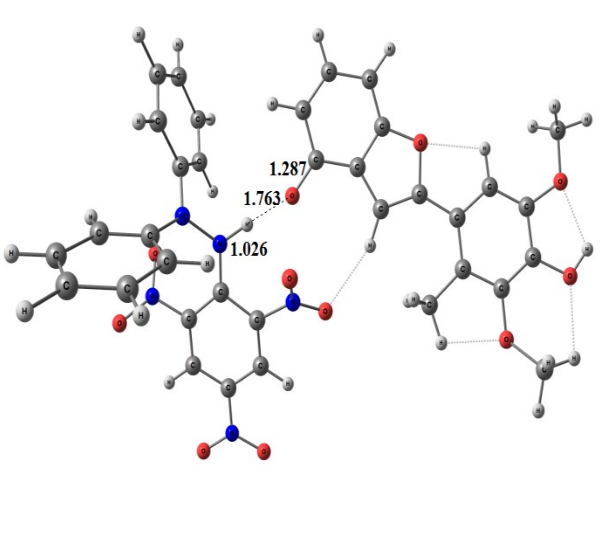

**12-**4’ -OH (Int-1)	**12-**4’ -OH (TS)	**12-**4’ -OH (Int-2)
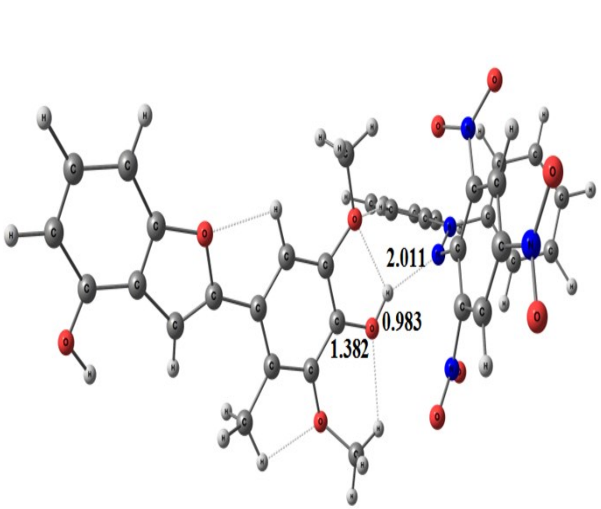	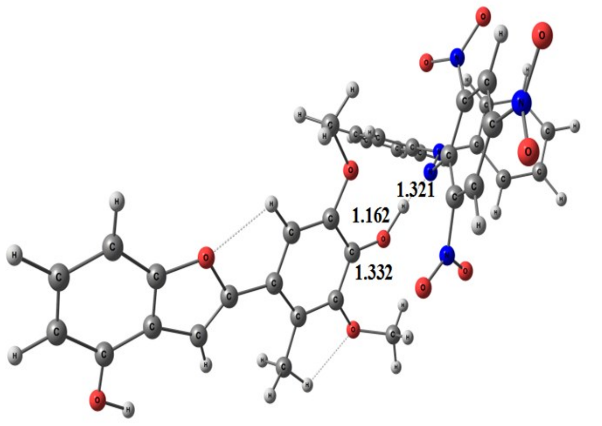	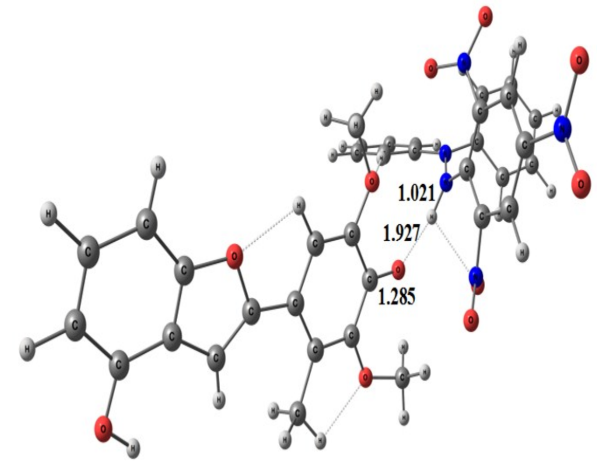
**13-**3’ -OH (Int-1)	**13-**3’ -OH (TS)	**13-**3’ -OH (Int-2)
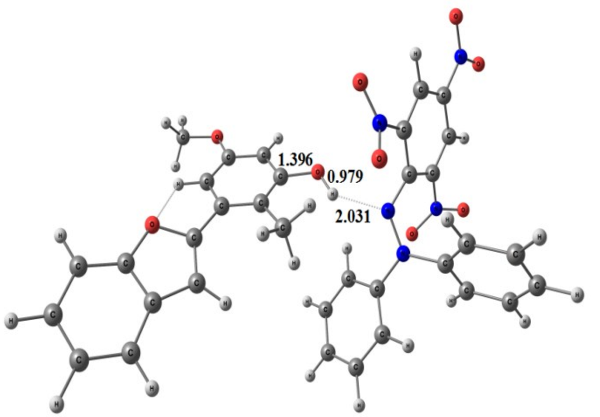	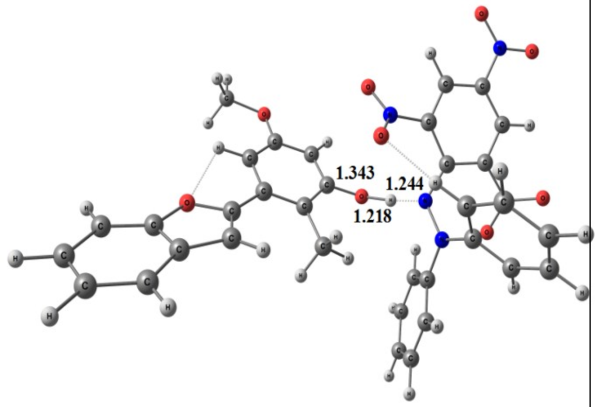	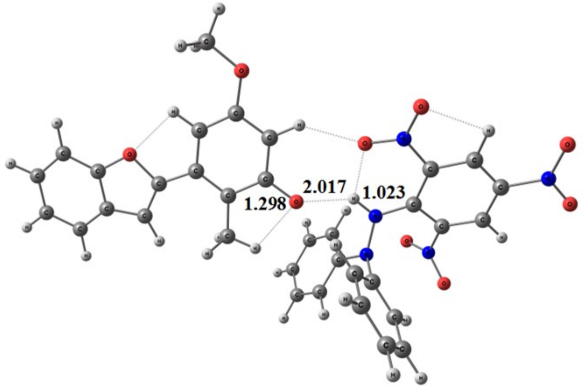

14-4’ -OH (Int-1)

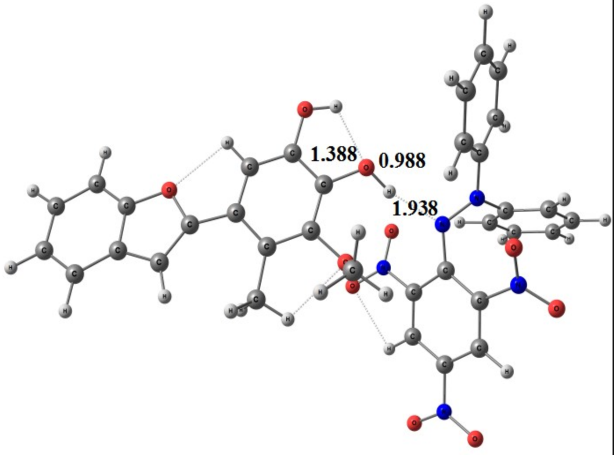

14-4’ -OH (TS)

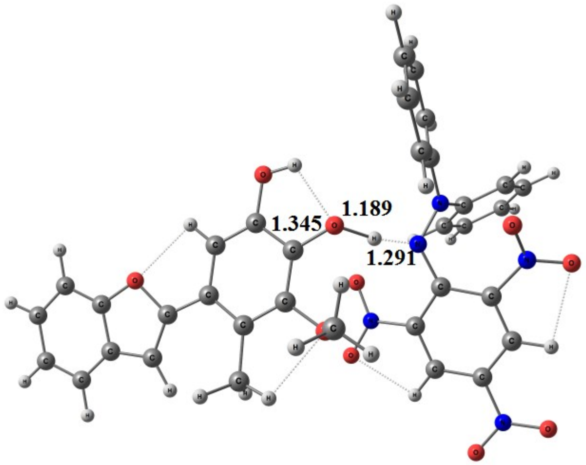

14-4’ -OH (Int-2)

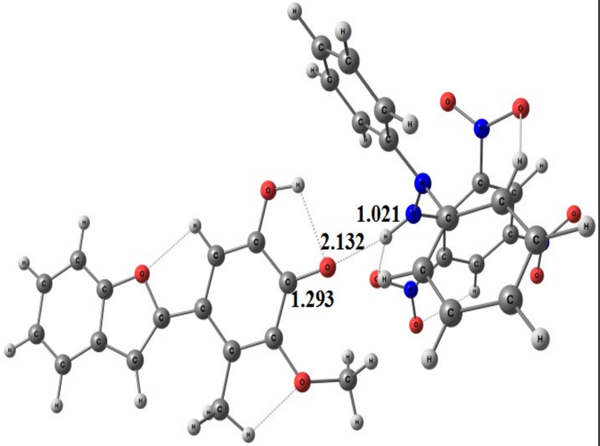

14-5’ -OH

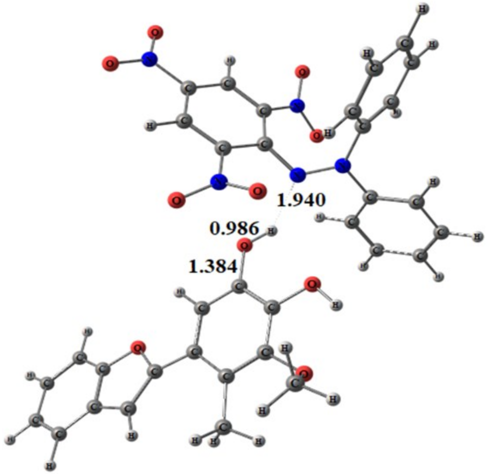

14-5’ -OH (TS)

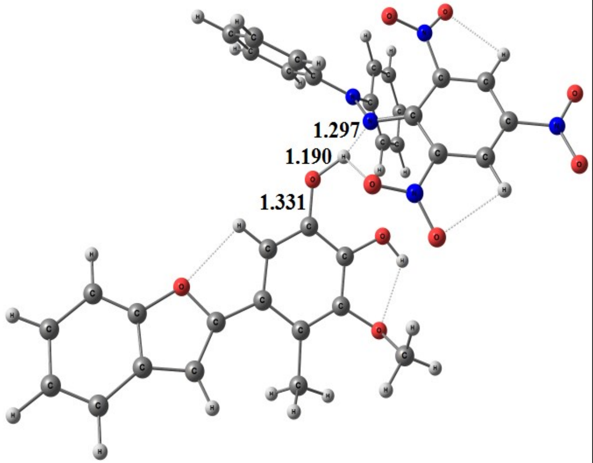

14-5’ -OH (Int-2)

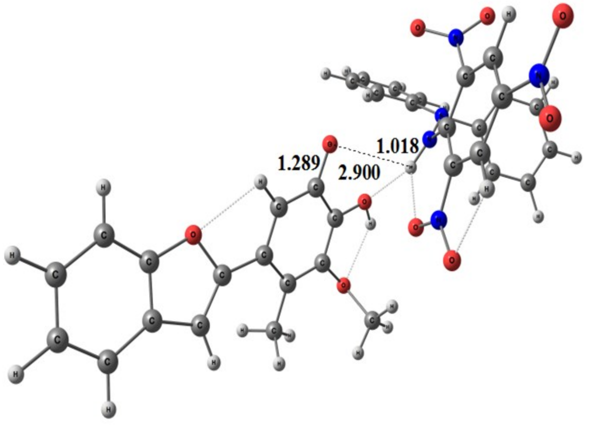

15-3’ -OH (Int-1)

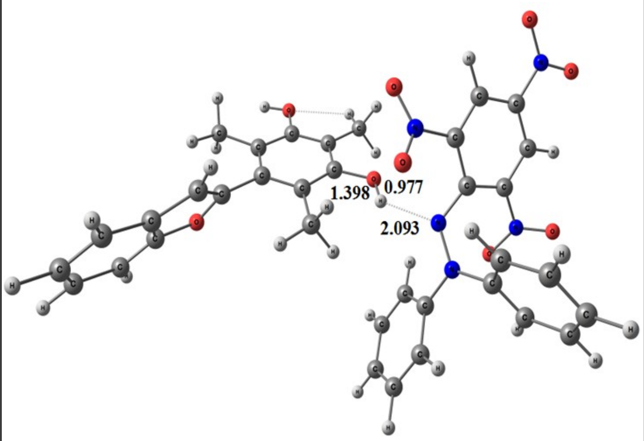

15-3’ -OH (TS)

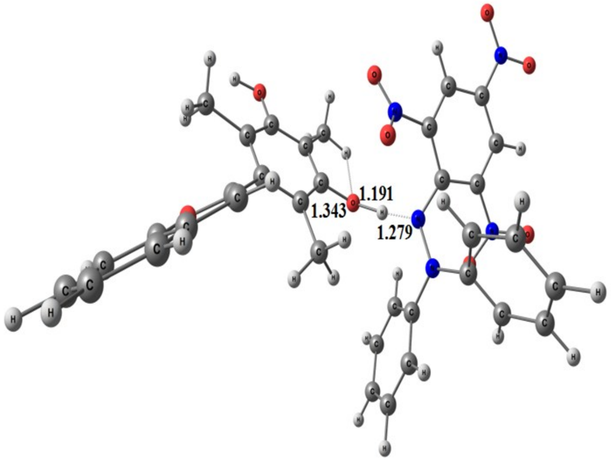

15-3’ -OH (Int-2)

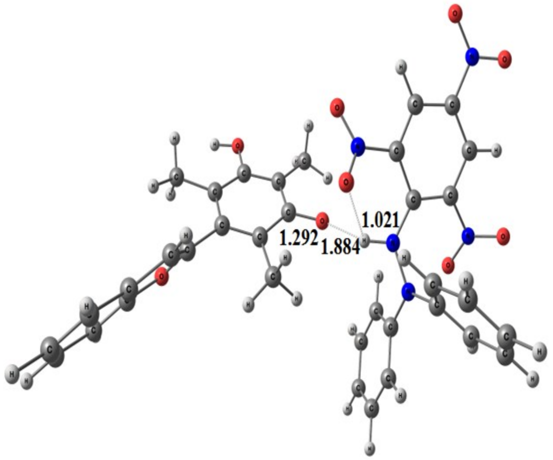

15-5’ -OH (Int-1)

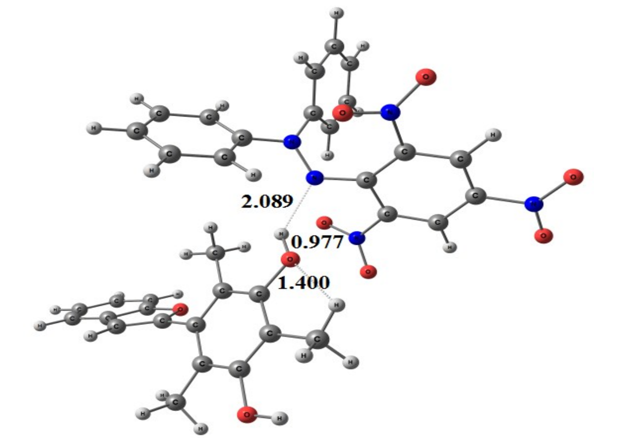

15-5’ -OH (TS)

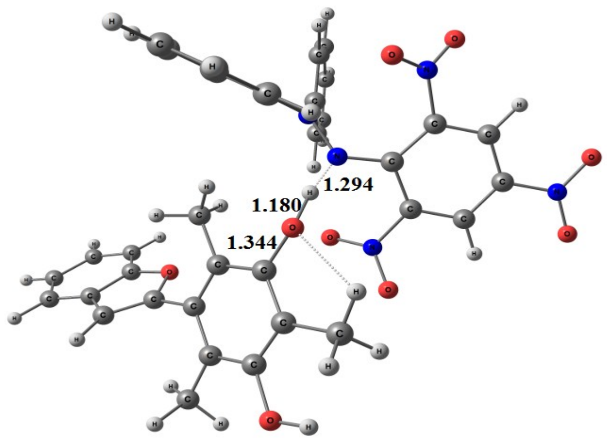

15-5’ -OH (Int-2)

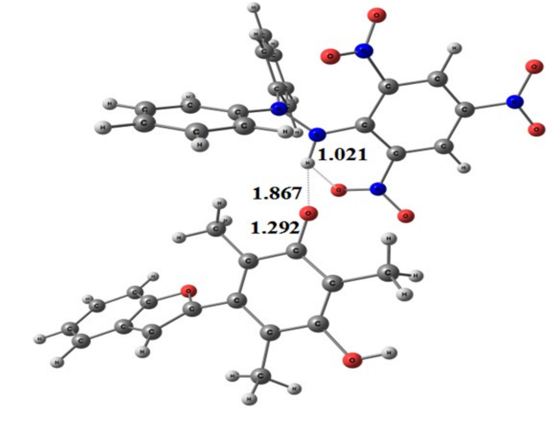

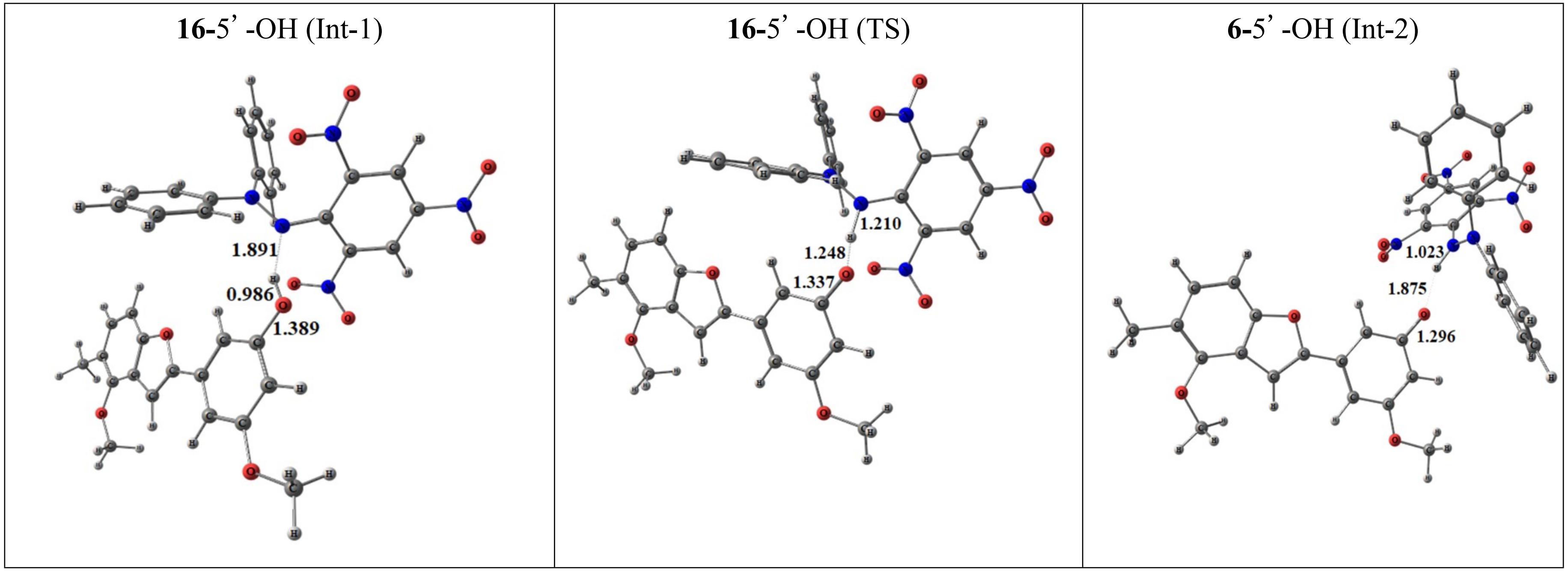

Fig. S4. The optimized structures of intermediates (Ints) and transition states (TSs) of DPPH radicals attack to the compounds **1-16** at B3LYP/6-311G level of theory

Table S1. The selective bond lengths and dihedral angles (θ) of the optimized structures **1-16** in the studied mediums at B3LYP/6-311++G(d,p) level of theory

No	Mediums	Bond lengths				Dihedral angles
		4(O-H)	3'(O-H)	4'(O-H)	5'(O-H)	θ_2 (C3-C2-C1'-C2')
**1**	Gas		0.963		0.963	0.002
	Water		0.965		0.965	−0.003
	Methanol		0.965		0.965	0.001
	Acetone		0.965		0.965	0.001
**2**	Gas	0.962	0.963			0.001
	Water	0.964	0.965			−0.001
	Methanol	0.964	0.965			0.001
	Acetone	0.964	0.965			0.001
**3**	Gas		0.963		0.963	0.003
	Water		0.964		0.965	0.002
	Methanol		0.964		0.965	0.003
	Acetone		0.964		0.964	0.001
**4**	Gas	0.962	0.963			−26.050
	Water	0.964	0.965			−28.794
	Methanol	0.964	0.965			−28.865
	Acetone	0.964	0.964			−28.860
**5**	Gas	0.962	0.962			−28.747
	Water	0.964	0.964			−28.747
	Methanol	0.964	0.964			−28.865
	Acetone	0.964	0.964			−28.709
**6**	Gas	0.962	0.962			−65.083
	Water	0.964	0.964			−64.588
	Methanol	0.964	0.964			−64.778
	Acetone	0.964	0.964			−64.923
**7**	Gas	0.962	0.962			−33.318

	Water	0.964	0.963			−34.109
	Methanol	0.964	0.963			−34.203
	Acetone	0.964	0.963			−34.270
**8**	Gas	0.962	0.962			−64.922
	Water	0.964	0.964			−64.490
	Methanol	0.964	0.964			−64.669
	Acetone	0.964	0.964			−64.769
**9**	Gas	0.962				22.810
	Water	0.964				26.707
	Methanol	0.964				26.527
	Acetone	0.964				26.253
**10**	Gas	0.962				22.831
	Water	0.964				26.603
	Methanol	0.964				26.334
	Acetone	0.964				25.893
**11**	Gas		0.962			−63.758
	Water		0.964			−64.015
	Methanol		0.964			−64.115
	Acetone		0.964			−64.065
**12**	Gas	0.962				−22.214
	Water	0.964				−25.627
	Methanol	0.964				−25.611
	Acetone	0.964				−25.635
**13**	Gas		0.962			−33.412
	Water		0.964			−34.049
	Methanol		0.964			−34.078
	Acetone		0.964			−34.049
**14**	Gas			0.968	0.966	23.540

	Water			0.969	0.967	24.723
	Methanol			0.969	0.967	24.592
	Acetone			0.969	0.967	24.521
**15**	Gas		0.962		0.962	64.499
	Water		0.964		0.964	67.615
	Methanol		0.964		0.964	67.505
	Acetone		0.964		0.964	67.435
**16**	Gas				0.963	0.005
	Water				0.965	0.004
	Methanol				0.965	0.010
	Acetone				0.965	0.008

Table S2. The relative energies depended on torsional angles θ_2 (C3-C2-C1'-C2') in the gaseous medium at B3LYP/6-31G(d,p) of theory

Degree	Compound **1** (au)	ΔE (kcal/mol)	Compound **2** (au)	ΔE (kcal/mol)	Compound **3** (au)	ΔE (kcal/mol)	Compound **4** (au)	ΔE (kcal/mol)
−180	−765.372125	**0.52**	−879.924052	**0.63**	−804.699862	**0.58**	−919.244155	**2.25**
−170	−765.372077	0.55	−879.924011	0.66	−804.699812	0.61	−919.244616	1.96
−160	−765.371847	0.70	−879.923804	0.79	−804.699580	0.75	−919.245372	1.48
−150	−765.371286	1.05	−879.923275	1.12	−804.699020	1.10	−919.245903	1.15
−140	−765.370364	1.63	−879.922367	1.69	−804.698101	1.68	−919.246046	**1.06**
−130	−765.369074	2.44	−879.921097	2.49	−804.696819	2.49	−919.245798	1.22
−120	−765.367490	3.43	−879.919550	3.46	−804.695242	3.47	−919.245220	1.58
−110	−765.365895	4.43	−879.917977	4.44	−804.693661	4.47	−919.244388	2.10
−100	−765.364597	5.25	−879.916745	5.22	−804.692385	5.27	−919.243508	2.65
−90	−765.364177	**5.51**	−879.916381	**5.45**	−804.691967	**5.53**	−919.243049	**2.94**
−80	−765.364748	5.15	−879.916957	5.08	−804.692520	5.18	−919.243135	2.89
−70	−765.366106	4.30	−879.918350	4.21	−804.693864	4.34	−919.243977	2.36
−60	−765.367771	3.26	−879.920025	3.16	−804.695520	3.30	−919.245167	1.61

−50	−765.369450	2.20	−879.921660	2.13	−804.697200	2.25	−919.246321	0.89
−40	−765.370867	1.31	−879.923045	1.26	−804.698622	1.35	−919.247198	0.34
−30	−765.371918	0.65	−879.924062	0.63	−804.699688	0.68	−919.247694	0.03
−20	−765.372581	0.24	−879.924666	0.25	−804.700369	0.26	−919.247735	**0.00**
−10	−765.372885	0.05	−879.924966	0.06	−804.700693	0.05	−919.247544	0.12
0	−765.372961	**0.00**	−879.925059	**0.00**	−804.700779	**0.00**	−919.247420	**0.20**
10	−765.372885	0.05	−879.924966	0.06	−804.700693	0.05	−919.247544	0.12
20	−765.372581	0.24	−879.924666	0.25	−804.700369	0.26	−919.247735	**0.00**
30	−765.371918	0.65	−879.924062	0.63	−804.699688	0.68	−919.247694	0.03
40	−765.370866	1.31	−879.923045	1.26	−804.698622	1.35	−919.247198	0.34
50	−765.369450	2.20	−879.921660	2.13	−804.697200	2.25	−919.246321	0.89
60	−765.367771	3.26	−879.920025	3.16	−804.695520	3.30	−919.245167	1.61
70	−765.366106	4.30	−879.918350	4.21	−804.693864	4.34	−919.243977	2.36
80	−765.364748	5.15	−879.916957	5.08	−804.692520	5.18	−919.243135	2.89
90	−765.364177	**5.51**	−879.916381	**5.45**	−804.691967	**5.53**	−919.243049	**2.94**
100	−765.364596	5.25	−879.916746	5.22	−804.692385	5.27	−919.243508	2.65
110	−765.365894	4.43	−879.917977	4.44	−804.693661	4.47	−919.244388	2.10
120	−765.367490	3.43	−879.919550	3.46	−804.695242	3.47	−919.245220	1.58
130	−765.369074	2.44	−879.921097	2.49	−804.696819	2.49	−919.245798	1.22

140	−765.370364	1.63	−879.922367	1.69	−804.698101	1.68	−919.246046	**1.06**
150	−765.371286	1.05	−879.923275	1.12	−804.699020	1.10	−919.245903	1.15
160	−765.371847	0.70	−879.923804	0.79	−804.699580	0.75	−919.245372	1.48
170	−765.372077	0.55	−879.924011	0.66	−804.699812	0.61	−919.244617	1.96
180	−765.372125	**0.52**	−879.924052	**0.63**	−804.699862	**0.58**	−919.244155	**2.25**
Degree	Compound **5** (au)	ΔE (kcal/mol)	Compound **6** (au)	ΔE (kcal/mol)	Compound **7** (au)	ΔE (kcal/mol)	Compound **8** (au)	ΔE (kcal/mol)
−180	−958.571491	**2.31**	−997.882821	**5.66**	−997.897863	**2.09**	−1037.210252	**5.66**
−170	−958.571979	2.00	−997.883945	4.95	−997.898866	1.47	−1037.211379	4.95
−160	−958.572755	1.52	−997.887424	2.77	−997.899883	0.83	−1037.214851	2.77
−150	−958.573291	1.18	−997.889040	1.76	−997.900562	0.40	−1037.216459	1.76
−140	−958.573437	**1.09**	−997.890173	1.04	−997.900789	**0.26**	−1037.217602	1.04
−130	−958.573201	1.24	−997.890762	0.67	−997.900583	0.39	−1037.218198	0.67
−120	−958.572627	1.60	−997.890931	**0.57**	−997.899992	0.76	−1037.218361	**0.57**
−110	−958.571803	2.12	−997.890802	0.65	−997.899133	1.30	−1037.218232	0.65
−100	−958.570945	2.65	−997.890768	**0.67**	−997.898233	1.86	−1037.218189	**0.67**
−90	−958.570496	**2.94**	−997.890983	0.54	−997.897709	2.19	−1037.218403	0.54
−80	−958.570583	2.88	−997.891370	0.29	−997.897701	**2.20**	−1037.218791	0.30
−70	−958.571423	2.35	−997.891707	0.08	−997.898407	1.75	−1037.219133	0.08

−60	−958.572603	1.61	−997.891837	**0.00**	−997.899432	1.11	−1037.219265	**0.00**
−50	−958.573752	0.89	−997.891565	0.17	−997.900395	0.51	−1037.218997	0.17
−40	−958.574626	0.34	−997.890771	0.67	−997.901009	0.12	−1037.218204	0.67
−30	−958.575125	0.03	−997.889408	1.52	−997.901202	**0.00**	−1037.216841	1.52
−20	−958.575174	**0.00**	−997.887393	2.79	−997.900923	0.17	−1037.214825	2.79
−10	−958.574983	0.12	−997.884827	4.40	−997.900278	0.58	−1037.212256	4.40
0	−958.574855	**0.20**	−997.882166	**6.07**	−997.899511	**1.06**	−1037.209580	**6.08**
10	−958.574983	0.12	−997.883615	5.16	−997.900279	0.58	−1037.211046	5.16
20	−958.575174	**0.00**	−997.886041	3.64	−997.900923	0.17	−1037.213466	3.64
30	−958.575124	0.03	−997.888104	2.34	−997.901202	**0.00**	−1037.215527	2.35
40	−958.574627	0.34	−997.889575	1.42	−997.901009	0.12	−1037.216996	1.42
50	−958.573752	0.89	−997.890433	0.88	−997.900395	0.51	−1037.217856	0.88
60	−958.572603	1.61	−997.890835	0.63	−997.899432	1.11	−1037.218253	0.63
70	−958.571423	2.35	−997.890930	0.57	−997.898407	1.75	−1037.218348	0.58
80	−958.570583	2.88	−997.890890	0.59	−997.897696	**2.20**	−1037.218322	0.59
90	−958.570496	**2.94**	−997.890896	0.59	−997.897709	2.19	−1037.218311	0.60
100	−958.570945	2.65	−997.891124	0.45	−997.898233	1.86	−1037.218526	0.46
110	−958.571803	2.12	−997.891441	0.25	−997.899133	1.30	−1037.218865	0.25
120	−958.572627	1.60	−997.891627	**0.13**	−997.899992	0.76	−1037.219054	**0.13**

130	−958.573201	1.24	−997.891547	0.18	−997.900586	0.39	−1037.218964	0.19
140	−958.573437	**1.09**	−997.891034	0.50	−997.900792	**0.26**	−1037.218443	0.52
150	−958.573291	1.18	−997.889987	1.16	−997.900567	0.40	−1037.217401	1.17
160	−958.572755	1.52	−997.888345	2.19	−997.899428	1.11	−1037.215773	2.19
170	−958.571979	2.00	−997.886183	3.55	−997.898893	1.45	−1037.213612	3.55
180	−958.571491	**2.31**	−997.882821	**5.66**	−997.897863	**2.09**	−1037.210252	**5.66**
Degree	Compound **9** (au)	ΔE (kcal/mol)	Compound **10** (au)	ΔE (kcal/mol)	Compound **11** (au)	ΔE (kcal/mol)	Compound **12** (au)	ΔE (kcal/mol)
−180	−1037.204413	**2.18**	−997.876969	**2.19**	−922.641814	**5.04**	−1033.792483	**2.45**
−170	−1037.205045	1.79	−997.877605	1.79	−922.643366	4.07	−1033.792945	2.16
−160	−1037.205807	1.31	−997.878375	1.31	−922.645367	2.81	−1033.793673	1.71
−150	−1037.206339	0.97	−997.878905	0.97	−922.647013	1.78	−1033.794206	1.37
−140	−1037.206454	**0.90**	−997.879015	**0.90**	−922.648121	1.09	−1033.794347	**1.28**
−130	−1037.206177	1.08	−997.878733	1.08	−922.648702	0.72	−1033.794110	1.43
−120	−1037.205522	1.49	−997.878090	1.48	−922.648871	**0.62**	−1033.793509	1.81
−110	−1037.204614	2.06	−997.877194	2.05	−922.648725	**0.71**	−1033.792675	2.33
−100	−1037.203607	2.69	−997.876203	2.67	−922.648750	0.69	−1033.791781	2.89
−90	−1037.202775	3.21	−997.875377	3.19	−922.648931	0.58	−1033.791260	**3.22**
−80	−1037.202551	**3.35**	−997.875149	**3.33**	−922.649329	0.33	−1033.791474	3.09

−70	−1037.203242	2.92	−997.875830	2.90	−922.649686	0.10	−1033.792334	2.55
−60	−1037.204400	2.19	−997.876978	2.18	−922.649851	**0.00**	−1033.793551	1.78
−50	−1037.205593	1.44	−997.878164	1.44	−922.649628	0.14	−1033.794751	1.03
−40	−1037.206544	0.85	−997.879109	0.84	−922.648874	0.61	−1033.795690	0.44
−30	−1037.207112	0.49	−997.879676	0.49	−922.647506	1.47	−1033.796231	0.10
−20	−1037.207357	0.34	−997.879924	0.33	−922.645522	2.72	−1033.796392	**0.00**
−10	−1037.207418	0.30	−997.879987	0.29	−922.642978	4.31	−1033.796308	0.05
0	−1037.207559	0.21	−997.880126	0.21	−922.640317	**5.98**	−1033.796264	**0.08**
10	−1037.207773	0.07	−997.880332	0.08	−922.641740	5.09	−1033.796308	0.05
20	−1037.207892	**0.00**	−997.880456	**0.00**	−922.644227	3.53	−1033.796392	**0.00**
30	−1037.207733	0.10	−997.880298	0.10	−922.646286	2.24	−1033.796231	0.10
40	−1037.207149	0.47	−997.879722	0.46	-922.647741	1.32	−1033.795690	0.44
50	−1037.206160	1.09	−997.878740	1.08	−922.648614	0.78	−1033.794751	1.03
60	−1037.204866	1.90	−997.877449	1.89	−922.648991	0.54	−1033.793551	1.78
70	−1037.203609	2.69	−997.876206	2.67	−922.649015	0.52	−1033.792334	2.55
80	−1037.202700	3.26	−997.875316	3.23	−922.648879	0.61	−1033.791474	3.09
90	−1037.202594	**3.32**	−997.875191	**3.30**	−922.648895	0.60	−1033.791260	**3.22**
100	−1037.203122	2.99	−997.875724	2.97	−922.649076	0.49	−1033.791781	2.89
110	−1037.203836	2.55	−997.876442	2.52	−922.649397	0.28	−1033.792675	2.33

120	−1037.204626	2.05	−997.877221	2.03	−922.649620	**0.14**	−1033.793509	1.81
130	−1037.205231	1.67	−997.877814	1.66	−922.649532	0.20	−1033.794110	1.43
140	−1037.205516	**1.49**	−997.878089	**1.49**	−922.649012	0.53	−1033.794347	**1.28**
150	−1037.205403	1.56	−997.877973	1.56	−922.647936	1.20	−1033.794206	1.37
160	−1037.204973	1.83	−997.877531	1.84	−922.646265	2.25	−1033.793673	1.71
170	−1037.204449	2.16	−997.877003	2.17	−922.644125	3.59	−1033.792945	2.16
180	−1037.204413	**2.18**	−997.876969	**2.19**	−922.641814	**5.04**	−1033.792483	**2.45**
Degree	Compound **13** (au)	ΔE (kcal/mol)	Compound **14** (au)	ΔE (kcal/mol)	Compound **15** (au)	ΔE (kcal/mol)	Compound **16** (au)	ΔE (kcal/mol)
−180	−844.000636	**2.05**	−919.245285	**1.26**	−883.333994	**6.47**	−958.557874	**0.69**
−170	−844.001612	1.43	−919.245814	0.93	−883.336674	4.78	−958.557822	0.72
−160	−844.002577	0.83	−919.246436	0.54	−883.339305	3.13	−958.557602	0.86
−150	−844.003235	0.42	−919.246825	0.30	−883.341412	1.81	−958.557093	1.18
−140	−844.003403	**0.31**	−919.246814	**0.30**	−883.342900	0.88	−958.556206	1.73
−130	−844.003144	0.47	−919.246428	0.55	−883.343765	0.33	−958.554927	2.54
−120	−844.002504	0.88	−919.245682	1.01	−883.344079	**0.14**	−958.553374	3.51
−110	−844.001687	1.39	−919.244696	1.63	−883.344005	0.18	−958.551798	4.50
−100	−844.000789	1.95	−919.243608	2.32	−883.343733	0.35	−958.550558	5.28
−90	−844.000251	**2.29**	−919.242685	2.90	−883.343628	**0.42**	−958.550202	**5.50**

−80	−844.000288	2.27	−919.242272	**3.15**	−883.343814	0.30	−958.550799	5.13
−70	−844.000988	1.83	−919.242499	3.01	−883.344125	0.11	−958.552206	4.24
−60	−844.002053	1.16	−919.243481	2.40	−883.344297	**0.00**	−958.553885	3.19
−50	−844.003034	0.54	−919.244652	1.66	−883.344134	0.10	−958.555541	2.15
−40	−844.003709	0.12	−919.245591	1.07	−883.343471	0.52	−958.556938	1.27
−30	−844.003898	**0.00**	−919.246163	0.71	−883.342216	1.31	−958.557950	0.64
−20	−844.003623	0.17	−919.246446	0.54	−883.340371	2.46	−958.558565	0.25
−10	−844.002969	0.58	−919.246596	0.44	−883.338059	3.91	−958.558875	0.06
0	−844.002227	**1.05**	−919.246782	0.32	−883.335807	**5.33**	−958.558968	**0.00**
10	−844.002969	0.58	−919.247107	0.12	−883.338059	3.91	−958.558875	0.06
20	−844.003623	0.17	−919.247299	**0.00**	−883.340371	2.46	−958.558565	0.25
30	−844.003898	**0.00**	−919.247156	0.09	−883.342216	1.31	−958.557950	0.64
40	−844.003709	0.12	−919.246608	0.43	−883.343471	0.52	−958.556938	1.27
50	−844.003034	0.54	−919.245662	1.03	−883.344134	0.10	−958.555541	2.15
60	−844.002053	1.16	−919.244457	1.78	−883.344297	**0.00**	−958.553885	3.19
70	−844.000988	1.83	−919.243296	2.51	−883.344125	0.11	−958.552206	4.24
80	−844.000288	2.27	−919.242562	2.97	−883.343814	0.30	−958.550798	5.13
90	−844.000251	**2.29**	−919.242559	**2.97**	−883.343628	**0.42**	−958.550202	**5.50**
100	−844.000789	1.95	−919.243088	2.64	−883.343733	0.35	−958.550558	5.28

110	−844.001687	1.39	−919.243942	2.11	−883.344005	0.18	−958.551798	4.50
120	−844.002504	0.88	−919.244772	1.59	−883.344079	**0.14**	−958.553374	3.51
130	−844.003144	0.47	−919.245407	1.19	−883.343765	0.33	−958.554927	2.54
140	−844.003403	**0.31**	−919.245754	0.97	−883.342900	0.88	−958.556206	1.73
150	−844.003235	0.42	−919.245791	**0.95**	−883.341412	1.81	−958.557093	1.18
160	−844.002577	0.83	−919.245514	1.12	−883.339305	3.13	−958.557602	0.86
170	−844.001612	1.43	−919.245211	**1.31**	−883.336674	4.78	−958.557822	0.72
180	−844.000636	**2.05**	−919.245285	1.26	−883.333994	**6.47**	−958.557874	**0.69**

Table S3. Condensed Fukui index $\Delta f_k = f_k^{+} - f_k^{-}$ of studied compounds **1-16** in gas medium at B3LYP/6-311++G(d,p) level of theory

Position	**1**	**2**	**3**	**4**	**5**	**6**	**7**	**8**	**9**	**10**	**11**	**12**	**13**	**14**	**15**	**16**
C−2	−0.042	0.059	−0.44	0.819	0.792	0.304	0.416	0.315	0.846	0.658	0.384	0.495	0.413	0.367	0.251	0.300
C−3	0.167	0.035	0.147	−0.181	−0.122	0.015	−0.236	−0.079	0.238	0.294	−0.054	0.286	0.734	−0.003	0.041	−0.397
C−3a	−0.236	−0.058	0.056	−1.309	−1.191	−0.325	−1.199	−0.196	−0.158	−0.45	−0.468	−0.120	−0.593	−0.352	−0.134	−0.242
C−4	−0.011	0.020	0.129	−0.932	−0.760	−0.088	−0.333	0.179	0.051	−0.018	−0.06	−0.047	−0.042	−0.196	−0.016	0.125
C−5	0.033	0.041	0.053	−0.333	−0.250	0.043	−0.242	1.775	−0.067	−0.001	−0.006	0.075	0.003	−0.155	0.008	−0.348
C−6	−0.02	−0.037	−0.037	0.090	0.031	−0.004	−0.184	−0.302	−0.230	−0.106	−0.007	−0.136	−0.067	0.05	0.015	0.750
C−7	−0.036	−0.019	−0.033	0.215	0.199	0.035	0.483	0.221	0.121	0.029	−0.059	−0.056	−0.116	−0.132	−0.169	0.051
C−7a	0.111	−0.167	−0.052	2.028	1.656	−0.273	1.707	−0.793	−0.344	−0.082	−0.05	−0.299	0.082	0.619	−0.127	−0.202
C−1'	0.012	0.008	−0.061	0.376	0.052	−0.162	0.081	−0.161	0.177	0.091	−0.201	0.253	0.430	−0.204	−0.078	0.213
C−2'	−0.096	0.070	−0.227	−0.013	−0.342	0.162	−0.436	0.104	−0.783	−0.635	0.033	−0.469	−0.418	−0.602	0.011	−0.166
C−3'	−0.035	−0.032	0.278	−0.324	−0.290	0.275	0.431	0.327	1.309	1.139	0.248	0.139	−0.050	0.537	−0.261	0.095
C−4'	0.359	−0.015	1.014	−0.128	0.339	0.037	0.186	0.129	−0.299	−0.326	0.041	0.090	0.043	−0.08	0.702	−0.124
C−5'	0.057	0.119	−1.019	1.294	0.350	0.078	−0.111	−0.103	−0.318	−0.209	0.085	0.206	−0.069	0.398	0.361	0.468
C−6'	0.057	−0.075	0.066	−0.383	−0.378	0.626	−0.295	0.608	−0.412	−0.457	0.738	−0.526	−0.114	−0.205	0.006	−0.404
2−O	0.000	−0.008	−0.003	−0.017	−0.018	−0.122	−0.044	−0.119	−0.003	−0.003	−0.109	−0.007	−0.014	−0.019	−0.065	0.100
$4-\underline{O}H$ or $(4-\underline{O}CH_3)$		0.036		0.022	0.016	0.044	0.018	0.055	0.017	0.012		0.011				−0.002

3'−OH or (3'-OCH_3)	−0.002	−0.005	0.012	−0.012	−0.007	0.033	0.016	0.030	−0.046	−0.040	0.046	−0.006	−0.006	−0.012	−0.005	0.057
4'−OH												0.040		0.043		
5'−OH or (5'-OCH_3)	0.013	0.014	0.027	0.006	0.001	0.021	−0.014	0.025	0.003	0.004	0.018	−0.009	0.010	0.029	0.029	−0.003

Table S4. Condensed Fukui index f_k^o of studied compounds **1-16** in gas medium at B3LYP/6-311++G(d,p) level of theory

Position	**1**	**2**	**3**	**4**	**5**	**6**	**7**	**8**	**9**	**10**	**11**	**12**	**13**	**14**	**15**	**16**
C−2	0.028	−0.098	−0.168	0.130	0.104	−0.028	0.077	−0.068	−0.048	−0.009	−0.018	0.157	0.063	0.051	−0.018	−0.236
C−3	0.100	−0.060	−0.078	−0.025	−0.040	−0.039	−0.015	−0.068	−0.046	−0.047	−0.079	−0.001	−0.098	−0.086	−0.074	−0.160
C−3a	−0.020	0.119	0.068	0.027	0.025	0.030	0.059	0.006	0.047	0.050	−0.001	0.017	0.044	−0.002	−0.011	−0.027
C−4	0.065	−0.086	−0.012	−0.089	−0.091	−0.135	−0.105	−0.177	−0.105	−0.072	−0.122	−0.107	−0.153	−0.100	−0.110	−0.189
C−5	0.021	−0.017	−0.014	−0.020	−0.007	0.037	0.056	0.107	0.086	0.025	−0.008	0.019	0.015	−0.001	−0.005	0.428
C−6	0.027	−0.030	−0.042	−0.046	−0.040	−0.029	−0.043	0.031	0.009	−0.033	−0.038	−0.037	−0.054	−0.036	−0.038	0.047
C−7	0.051	−0.020	−0.051	−0.031	−0.041	−0.038	−0.011	−0.042	−0.024	−0.035	−0.042	−0.044	−0.013	−0.038	−0.054	−0.085
C−7a	−0.028	−0.015	−0.013	−0.112	−0.096	−0.059	−0.153	−0.028	−0.053	−0.048	0.009	−0.114	−0.010	0.009	0.010	0.045
C−1'	−0.030	−0.012	0.004	0.084	0.033	−0.006	0.008	0.001	−0.007	−0.006	−0.027	0.030	0.049	−0.036	−0.008	−0.113
C−2'	−0.033	0.000	−0.103	0.080	−0.013	0.070	−0.057	0.064	0.008	0.009	0.064	−0.204	0.021	−0.011	0.005	0.052
C−3'	0.025	−0.038	0.072	−0.151	−0.230	−0.080	−0.366	−0.058	0.117	0.049	−0.052	0.159	−0.139	−0.030	−0.161	−0.011
C−4'	0.080	−0.086	0.627	−0.120	0.148	0.197	0.166	0.188	−0.030	−0.011	0.179	0.065	−0.059	−0.048	0.203	0.189
C−5'	0.070	−0.071	−0.657	0.485	−0.074	−0.121	0.194	−0.136	−0.128	−0.096	−0.101	−0.178	−0.030	−0.039	−0.020	−0.319
C−6'	0.079	0.001	−0.048	−0.038	−0.022	0.030	−0.072	0.046	−0.043	−0.088	0.052	−0.168	−0.003	−0.022	0.041	0.025
2−O	0.052	−0.051	−0.049	−0.045	−0.045	−0.041	−0.046	−0.043	−0.044	−0.044	−0.037	−0.043	−0.045	−0.046	−0.040	−0.051
4−<u>O</u>H or (4-<u>O</u>CH_3)		−0.068		−0.066	−0.064	−0.063	−0.061	−0.063	−0.064	−0.063		−0.061				−0.047

3'−OH<br>or<br>(3'-OCH_3)	−0.038	−0.035	−0.033	−0.034	−0.029	−0.045	−0.039	−0.043	−0.014	−0.013	−0.050	−0.007	−0.037	−0.004	−0.032	−0.016
4'−OH												−0.082		−0.083		
5'−OH<br>or<br>(5'-OCH_3)	−0.048	−0.029	−0.035	−0.031	−0.025	−0.003	−0.019	−0.003	−0.023	−0.024	−0.004	−0.012	−0.035	−0.049	−0.042	−0.039

Table S5. Theoretical and experimental ^{1}H-NMR chemical shift values with respect to TMS in acetone-d_6 and $CDCl_3$ solutions of studied compounds at B3LYP/6-311++G(d,p) level of theory

**No**	**1**		**2**		**3**		**4**		**5**		**6**		**7**		**8**	
	Theo.	Exp.	Theo.	Exp.	Theo.	Exp.	Theo.	Exp.	Theo.	Exp.	Theo.	Exp.	Theo.	Exp.	Theo.	Exp.
3	7.34	7.16	7.32	7.27	7.30	7.03	6.90	7.09	6.86	7.03	6.67	6.80	6.85	7.10	6.66	6.84
4	7.75	7.62			7.69	7.59										
5	7.48	7.23	6.81	6.69	7.46	7.22	7.06	6.70	7.07	6.69	7.06	6.71				
5-Me													2.36	2.31	2.33	2.31
6	7.57	7.30	7.29	7.12	7.55	7.27	7.55	7.15	7.54	7.13	7.47	7.12	7.27	7.05	7.33	7.04
7	7.73	7.52	7.23	7.05	7.77	7.50	7.20	7.07	7.19	7.06	7.26	7.03	7.08	6.98	7.19	6.96
2'	7.09	6.93	7.11	7.00	7.44	6.99										
2'-Me							2.44	2.36	2.50	2.40	2.08	2.08	2.37	2.38	2.07	2.07
4'	6.45	6.42	6.55	6.44			6.49	6.56								
4'-Me					2.15	2.14			2.17	2.17	2.27	2.25	2.20	2.17	2.27	2.24
5'-OMe			3.90	3.83			3.85	3.80	3.90	3.87	3.66	3.69	3.90	3.87	3.66	3.69
6'	7.12	6.93	7.06	6.98	6.83	6.99	7.06	6.90	7.10	6.94			6.99	6.93		
6'-Me											2.07	2.06			2.07	2.05
**No**	**9**		**10**		**11**		**12**		**13**		**14**		**15**		**16**	

	Theo.	Exp.	Theo.	Exp.	Theo.	Exp.	Theo.	Exp.	Theo.	Exp.	Theo.	Exp.	Theo.	Exp.	Theo.	Exp.
3	6.79	7.18	6.64	7.10	6.78	6.76	6.69	6.99	6.89	6.86	6.78	6.78	6.83	6.63	7.66	7.09
4					7.79	7.52			7.88	7.61	7.73	7.50	7.88	7.51		
4-OMe															4.42	4.05
5			7.05	6.71	7.55	7.27	7.05	6.68	7.45	7.25	7.48	7.28	7.40	7.30		
5-Me	2.36	2.30													2.13	2.33
6	7.28	7.06	7.43	7.15	7.53	7.31	7.47	7.07	7.48	7.30	7.45	7.23	7.41	7.26	7.25	7.07
7	7.18	6.99	7.22	7.07	7.71	7.67	7.16	7.04	7.68	7.62	7.68	7.58	7.69	7.61	7.09	7.14
2'															6.98	6.91
2'-Me	2.49	2.41	2.46	2.43	2.06	2.07	2.41	2.39	2.32	2.34	2.46	2.42	1.85	2.06		
3'-OMe	3.69	3.72	3.67	3.73			3.92	3.81			3.80	3.85				
4'									6.80	6.51					6.38	6.39
4'-Me	2.21	2.17	2.22	2.17	2.26	2.25							2.22	2.24		
5'																
5'-OMe	3.98	3.90	3.96	3.91	3.63	3.69	4.03	3.90	3.84	3.86					3.79	3.86
6'	7.45	7.18	7.47	7.19			7.42	7.17	7.02	6.91	7.79	7.23			6.96	6.97
6'-Me					2.04	2.05							2.04	2.06		

^{1}H-NMR experience of **1-12** were measured in acetone-d_6, while ^{1}H-NMR experience of **13-16** were recorded in $CDCl_3$. In

comparison between observed and calculated ^{1}H-NMR values, the R^2 coefficients were found in compounds **1**: $R^2 = 0.9802$, **2**: $R^2 = 0.9986$, **3**: $R^2 = 0.9912$, **4**: $R^2 = 0.9889$, **5**: $R^2 = 0.9937$, **6**: $R^2 = 0.9964$, **7**: $R^2 = 0.9969$, **8**: $R^2 = 0.9968$, **9**: $R^2 = 0.9930$, **10**: $R^2 = 0.9886$, **11**: $R^2 = 0.9988$, **12**: $R^2 = 0.9882$, **13**: $R^2 = 0.9978$, **14**: $R^2 = 0.9938$, **15**: $R^2 = 0.9986$, and **16**: $R^2 = 0.9860$

Table S6. Theoretical and experimental ^{13}C-NMR chemical shift values with respect to TMS in acetone-d_6 and $CDCl_3$ solutions of studied compounds at B3LYP/6-311++G(d,p) level of theory

**No**	**1**		**2**		**3**		**4**		**5**		**6**		**7**		**8**	
	Theo.	Exp.	Theo.	Exp.	Theo.	Exp.	Theo.	Exp.	Theo.	Exp.	Theo.	Exp.	Theo.	Exp.	Theo.	Exp.
2	162.3		161.9	155.0	162.7	157.3	161.4	154.7	162.0	155.0	162.6	154.0	161.9	155.0	162.6	
3	106.9	102.3	101.3	100.0	105.6	101.4	106.1	103.2	105.1	103.1	106.7	104.2	106.0	103.0	106.5	104.0
3a	135.9		121.7	119.4	136.1	130.2	121.5	119.6	121.6	119.4	121.0	119.0	121.7	119.6	120.3	
4	125.7	121.9	156.4	157.2	125.3	121.7	156.3	156.7	156.2	156.9	156.4	156.4	153.1	156.8	153.8	
5	128.4	124.0	111.7	108.8	128.3	123.8	112.3	108.3	112.2	108.6	112.6	108.6	123.2	117.6	123.4	
5-Me													17.08	15.70	14.18	15.60
6	129.7	125.2	130.7	126.2	129.2	124.9	131.0	125.9	130.6	125.9	130.4	125.5	132.1	127.9	131.3	127.5
7	115.3	111.7	106.8	103.5	115.3	111.6	106.6	103.2	106.5	103.5	106.8	103.6	105.8	103.1	106.2	103.2
7a	161.3		162.4	152.1	161.3	155.5	162.4	151.8	162.5	151.9	162.7	151.8	160.7	148.7	161.4	
1'	138.1		136.9	133.2	134.7	129.2	137.5	132.6	134.2	129.2	134.1	130.3	133.9	129.3	134.3	
2'	105.2	104.3	106.5	105.1	106.4	104.1	120.5	115.5	119.8	122.6	123.6	123.2	116.6	116.2	122.5	
2'-Me							13.1	12.90	13.5	13.80	12.1	14.10	12.0	13.80	12.08	14.00

3'	165.5		165.2	159.8	164.3	157.5	162.6	157.1	161.2	155.4	158.2	152.8	160.0	155.2	158.2	
4'	104.8	104.1	107.7	102.4	113.7	113.0	106.5	102.3	115.6	114.0	125.1	120.3	118.3	114.0	125.0	
4'-Me					6.02	8.70			5.69	9.10	9.58	9.90	8.62	9.10	9.53	9.80
5'	165.2		168.8	162.4	162.9	157.5	165.1	159.3	161.9	157.1	160.8	157.3	163.5	157.1	160.8	
5'-OMe			55.9	55.7			55.91	55.4	55.9	56.0	60.9	60.20	56.0	55.90	60.9	60.2
6'	106.4	104.3	100.3	102.6	104.8	104.1	104.2	105.1	103.6	103.4	131.2	121.4	104.3	103.2	131.1	
6'-Me											14.17	13.50			14.6	13.50
**No**	**9**		**10**		**11**		**12**		**13**		**14**		**15**		**16**	
	Theo.	Exp.	Theo.	Exp.	Theo.	Exp.	Theo.	Exp.	Theo.	Exp.	Theo.	Exp.	Theo.	Exp.	Theo.	Exp.
2	161.9	154.7	162.1	154.8	164.2		162.1		163.1	155.7	162.6	155.4	162.8	154.7	160.0	156.9
3	104.6	103.4	104.5	103.4	112.1		103.5	103.1	111.1	106.1	109.1	104.4	112.9	106.5	104.3	99.80
3a	121.5	119.4	121.7	119.4	134.7		121.8		135.1	129.5	135.3	129.2	134.9	129.0	119.8	120.3
4	153.8	155.1	156.6	156.8	124.4		155.9	157.2	125.3	121.5	124.5	120.7	125.1	120.8	157.3	150.8
4-OMe															58.3	60.20
5	123.2	117.4	112.2	108.7	128.4		111.8	108.9	127.2	123.1	127.4	122.7	127.0	122.7	126.6	122.2
5-Me	17.1	15.70													17.6	15.80

6	132.1	128.1	130.4	126.1	128.5		130.0	126.4	129.0	124.6	128.3	124.0	128.2	123.9	131.3	127.4
7	105.8	103.1	106.6	103.4	115.0		106.2	103.7	115.1	111.5	114.8	111.0	115.3	111.3	105.4	105.8
7a	160.9	148.8	162.5	152.0	161.1		161.9	155.9	161.0	155.0	160.8	154.3	161.4	154.8	162.7	154.8
1'	133.4	129.8	133.1	129.7	133.7		126.6		137.6	132.2	128.1	122.8	134.8	128.7	137.9	
2'	127.8	121.8	128.0	121.9	121.9		130.1	123.5	116.5	117.8	126.7	121.2	118.8	108.6	103.4	104.5
2'-Me	14.23	13.80	14.32	13.8	12.05		14.5	13.8	11.4	13.0	15.28	13.70	12.4	13.10		
3'	164.2	159.0	164.1	159.0	162.1		150.7	148.0	163.4	155.7	150.8	146.1	157.1	150.6	165.9	155.1
3'-OMe	61.02	60.40	61.06	60.3			59.94	60.90			61.3	61.0				
4'	127.7	120.7	127.8	120.8	125.9		147.0	142.1	108.0	99.8	142.4	136.9	113.4	115.6	99.5	101.9
4'-Me	9.37	9.50	9.42	9.50	9.48								5.77	8.80		
5'	163.6	157.5	163.7	157.5	162.1		151.0	148.2	165.9	159.7	148.9	142.2	159.8	150.6	169.0	161.8
5'-OMe	56.1	56.0	56.06	56.0	60.4		56.6	56.80	55.5	60.0					55.6	56.00
6'	107.6	106.4	107.6	106.6	131.4		107.0	108.7	104.9	107.2	114.4	111.5	122.2	108.6	108.1	103.0
6'-Me					13.9								14.19	13.10		

^{1}H-NMR experience of **1-10**, and **12** were measured in acetone-d_6, while ^{1}H-NMR experience of **13-16** were recorded in $CDCl_3$. In comparison between observed and calculated ^{13}C-NMR values, the R^2 coefficients were found in compounds **2**: R^2 = 0.9929, **3**: R^2 = 0.9991, **4**: R^2 = 0.9964, **5**: R^2 = 0.9966, **6**: R^2 = 0.9971, **7**: R^2 = 0.9964, **9**: R^2 = 0.9972, **10**: R^2 = 0.9976, **13**: R^2 = 0.9953, **14**: R^2 = 0.9993,

and **15**: $R^2 = 0.9957$.

Table S7. Optimized geometries (in Cartesian coordinates) of studied compounds **1-16** at B3LYP/6-311++G(d,p) level of theory in gas medium

**Compound**	Cartesian Coordinate			
1	C	2.516189000000	-0.826945000000	-0.000073000000
	C	2.386767000000	0.572636000000	0.000112000000
	C	3.464208000000	1.444971000000	0.000243000000
	C	4.731406000000	0.865987000000	0.000183000000
	C	4.896094000000	-0.530415000000	0.000000000000
	C	3.802058000000	-1.387156000000	-0.000129000000
	C	1.172213000000	-1.334750000000	-0.000163000000
	H	3.322906000000	2.518547000000	0.000383000000
	H	5.606783000000	1.504942000000	0.000281000000
	H	0.869715000000	-2.369422000000	-0.000310000000
	C	0.338951000000	-0.254411000000	-0.000028000000
	O	1.065571000000	0.921621000000	0.000139000000
	C	-1.113456000000	-0.121673000000	-0.000031000000
	C	-1.922604000000	-1.263514000000	-0.000166000000
	C	-1.702542000000	1.152534000000	0.000108000000
	C	-3.307770000000	-1.124123000000	-0.000175000000
	C	-3.089810000000	1.267618000000	0.000100000000
	C	-3.904350000000	0.135927000000	-0.000050000000
	H	-1.076430000000	2.036245000000	0.000221000000
	H	-1.501083000000	-2.259668000000	-0.000261000000
	H	-4.982054000000	0.256393000000	-0.000069000000

	H	5.898377000000	-0.943179000000	-0.000042000000
	H	3.939986000000	-2.462310000000	-0.000271000000
	O	-4.046671000000	-2.275403000000	-0.000331000000
	H	-4.985435000000	-2.061559000000	-0.000134000000
	O	-3.722539000000	2.480438000000	0.000231000000
	H	-3.067782000000	3.186644000000	0.000294000000
**2**	C	2.573209000000	-0.440933000000	-0.000055000000
	C	2.205481000000	0.914818000000	0.000170000000
	C	3.108363000000	1.967332000000	0.000343000000
	C	4.455386000000	1.611592000000	0.000284000000
	C	4.869976000000	0.271676000000	0.000065000000
	C	3.941030000000	-0.762011000000	-0.000105000000
	C	1.339264000000	-1.179450000000	-0.000183000000
	H	2.781473000000	2.998583000000	0.000513000000
	H	5.209215000000	2.390112000000	0.000412000000
	H	1.204805000000	-2.249652000000	-0.000365000000
	C	0.329796000000	-0.261053000000	-0.000029000000
	O	0.842919000000	1.020731000000	0.000187000000
	C	-1.123634000000	-0.386225000000	-0.000055000000
	C	-1.715972000000	-1.651828000000	-0.000247000000
	C	-1.923686000000	0.770551000000	0.000113000000
	C	-3.105480000000	-1.757779000000	-0.000282000000
	C	-3.311370000000	0.641801000000	0.000080000000
	C	-3.909732000000	-0.623294000000	-0.000126000000

	H	-1.447537000000	1.738416000000	0.000264000000
	H	-1.126597000000	-2.558936000000	-0.000372000000
	H	-4.992046000000	-0.692415000000	-0.000160000000
	H	5.923623000000	0.021212000000	0.000025000000
	O	-3.630133000000	-3.022125000000	-0.000491000000
	H	-4.591822000000	-2.975921000000	-0.000378000000
	O	-4.178417000000	1.693484000000	0.000228000000
	O	4.400923000000	-2.050584000000	-0.000321000000
	H	3.661024000000	-2.665702000000	-0.000394000000
	C	-3.649261000000	3.014681000000	0.000404000000
	H	-3.046141000000	3.202336000000	0.894671000000
	H	-3.046121000000	3.202564000000	-0.893802000000
	H	-4.512429000000	3.678071000000	0.000480000000
**3**	C	-2.883639000000	-0.817773000000	0.000051000000
	C	-2.739704000000	0.580477000000	-0.000167000000
	C	-3.807713000000	1.464056000000	-0.000334000000
	C	-5.081356000000	0.898824000000	-0.000274000000
	C	-5.260625000000	-0.495579000000	-0.000058000000
	C	-4.175458000000	-1.363934000000	0.000108000000
	C	-1.544885000000	-1.339638000000	0.000167000000
	H	-3.654867000000	2.536083000000	-0.000498000000
	H	-5.949824000000	1.547155000000	-0.000399000000
	H	-1.252842000000	-2.377317000000	0.000343000000
	C	-0.700263000000	-0.268016000000	0.000018000000

	O	-1.414751000000	0.915680000000	-0.000186000000
	C	0.752561000000	-0.149901000000	0.000035000000
	C	1.555878000000	-1.294416000000	0.000174000000
	C	1.359993000000	1.112979000000	-0.000105000000
	C	2.940259000000	-1.165092000000	0.000206000000
	C	2.748205000000	1.216499000000	-0.000077000000
	C	3.575881000000	0.086068000000	0.000097000000
	H	0.746464000000	2.005848000000	-0.000234000000
	H	1.130896000000	-2.289312000000	0.000259000000
	H	-6.267175000000	-0.897919000000	-0.000018000000
	H	-4.324965000000	-2.437564000000	0.000274000000
	O	3.661964000000	-2.328736000000	0.000382000000
	H	4.604368000000	-2.132500000000	0.000056000000
	O	3.368481000000	2.441114000000	-0.000216000000
	H	2.702197000000	3.135928000000	-0.000221000000
	C	5.079132000000	0.199474000000	0.000166000000
	H	5.522049000000	-0.271363000000	0.886650000000
	H	5.522116000000	-0.270926000000	-0.886521000000
	H	5.386436000000	1.243268000000	0.000422000000
**4**	C	2.618033000000	-0.350330000000	0.133855000000
	C	2.268991000000	0.939991000000	-0.293574000000
	C	3.185373000000	1.946264000000	-0.561858000000
	C	4.525069000000	1.612300000000	-0.376623000000
	C	4.920844000000	0.336690000000	0.052207000000

	C	3.978727000000	-0.652127000000	0.309512000000
	C	1.375949000000	-1.060645000000	0.283984000000
	H	2.873385000000	2.927518000000	-0.893783000000
	H	5.288446000000	2.357097000000	-0.568992000000
	H	1.232453000000	-2.069527000000	0.630876000000
	C	0.375355000000	-0.192375000000	-0.050311000000
	O	0.911693000000	1.035721000000	-0.402658000000
	C	-1.086960000000	-0.265081000000	-0.047987000000
	C	-1.778280000000	-1.486251000000	-0.170010000000
	C	-1.783376000000	0.953465000000	0.094112000000
	C	-3.183208000000	-1.432171000000	-0.104296000000
	C	-3.170941000000	0.962036000000	0.140847000000
	C	-3.876467000000	-0.240183000000	0.049144000000
	H	-1.215398000000	1.866926000000	0.174257000000
	H	-4.960029000000	-0.219972000000	0.097779000000
	H	5.969318000000	0.101510000000	0.187515000000
	O	-3.854557000000	-2.625733000000	-0.205311000000
	H	-4.802933000000	-2.461036000000	-0.205909000000
	O	-3.935599000000	2.083253000000	0.282368000000
	O	4.418949000000	-1.879781000000	0.724064000000
	H	3.671511000000	-2.472134000000	0.851648000000
	C	-3.280499000000	3.341802000000	0.384137000000
	H	-2.631181000000	3.383231000000	1.264973000000
	H	-2.691973000000	3.558783000000	-0.513411000000

	H	-4.073521000000	4.080988000000	0.484436000000
	C	-1.113218000000	-2.820582000000	-0.398278000000
	H	-1.810595000000	-3.514364000000	-0.865715000000
	H	-0.237779000000	-2.721232000000	-1.042267000000
	H	-0.787939000000	-3.282284000000	0.541728000000
**5**	C	2.912367000000	-0.368389000000	0.133706000000
	C	2.576959000000	0.927308000000	-0.288079000000
	C	3.503598000000	1.926428000000	-0.547607000000
	C	4.839712000000	1.579291000000	-0.359805000000
	C	5.221944000000	0.297776000000	0.063539000000
	C	4.269485000000	-0.683447000000	0.312079000000
	C	1.663039000000	-1.067749000000	0.276906000000
	H	3.201840000000	2.912294000000	-0.875397000000
	H	5.610577000000	2.318024000000	-0.545625000000
	H	1.508875000000	-2.076625000000	0.619288000000
	C	0.671470000000	-0.188550000000	-0.056372000000
	O	1.220862000000	1.036226000000	-0.401387000000
	C	-0.790642000000	-0.246870000000	-0.059508000000
	C	-1.500064000000	-1.455669000000	-0.182089000000
	C	-1.480282000000	0.972733000000	0.079463000000
	C	-2.902605000000	-1.383468000000	-0.117274000000
	C	-2.867289000000	0.999574000000	0.123663000000
	C	-3.611805000000	-0.188935000000	0.034063000000
	H	-0.906022000000	1.882021000000	0.160710000000

	H	6.267687000000	0.051959000000	0.201180000000
	O	4.696654000000	-1.917833000000	0.721579000000
	H	3.942708000000	-2.503295000000	0.842479000000
	O	-3.577395000000	-2.576527000000	-0.216126000000
	H	-4.525661000000	-2.413154000000	-0.229758000000
	O	-3.595584000000	2.150444000000	0.263475000000
	C	-0.851792000000	-2.798694000000	-0.408638000000
	H	-1.553864000000	-3.482297000000	-0.883709000000
	H	0.030419000000	-2.708546000000	-1.044608000000
	H	-0.540293000000	-3.266798000000	0.532976000000
	C	-5.118667000000	-0.186218000000	0.096436000000
	H	-5.565855000000	-0.586873000000	-0.822531000000
	H	-5.490712000000	-0.782251000000	0.938876000000
	H	-5.494662000000	0.826649000000	0.222050000000
	C	-2.903168000000	3.388282000000	0.362173000000
	H	-2.252048000000	3.412630000000	1.242268000000
	H	-2.309068000000	3.586112000000	-0.536045000000
	H	-3.673770000000	4.151258000000	0.461571000000
**6**	C	-2.994521000000	0.260879000000	0.330152000000
	C	-2.688967000000	-0.650895000000	-0.693150000000
	C	-3.639397000000	-1.371716000000	-1.402511000000
	C	-4.965718000000	-1.148622000000	-1.041183000000
	C	-5.317230000000	-0.251534000000	-0.021707000000
	C	-4.342315000000	0.456591000000	0.671057000000

	C	-1.725048000000	0.778121000000	0.779567000000
	H	-3.360530000000	-2.062670000000	-2.186866000000
	H	-5.753487000000	-1.682835000000	-1.559382000000
	H	-1.527923000000	1.492754000000	1.564189000000
	C	-0.765040000000	0.173726000000	0.030370000000
	O	-1.334779000000	-0.703951000000	-0.875342000000
	C	0.706990000000	0.263597000000	0.031254000000
	C	1.341589000000	1.473347000000	-0.314914000000
	C	1.452299000000	-0.875396000000	0.393779000000
	C	2.740145000000	1.521939000000	-0.257687000000
	C	2.850280000000	-0.774717000000	0.398636000000
	C	3.519053000000	0.417044000000	0.098736000000
	H	-6.356120000000	-0.097285000000	0.242700000000
	O	-4.740415000000	1.321766000000	1.655008000000
	H	-3.970878000000	1.718243000000	2.075021000000
	O	3.433028000000	2.670206000000	-0.560894000000
	H	2.817579000000	3.381244000000	-0.763930000000
	O	3.601485000000	-1.871484000000	0.786802000000
	C	3.955419000000	-2.759379000000	-0.275097000000
	H	3.065813000000	-3.175607000000	-0.759715000000
	H	4.567545000000	-2.250662000000	-1.028133000000
	H	4.532757000000	-3.566745000000	0.175768000000
	C	0.589373000000	2.703474000000	-0.769156000000
	H	0.987008000000	3.083849000000	-1.718233000000

	H	-0.465778000000	2.492021000000	-0.929408000000
	H	0.646829000000	3.517350000000	-0.034989000000
	C	5.018399000000	0.540148000000	0.161721000000
	H	5.448812000000	0.653918000000	-0.838736000000
	H	5.305562000000	1.430441000000	0.726025000000
	H	5.457357000000	-0.335720000000	0.636400000000
	C	0.783042000000	-2.163406000000	0.812197000000
	H	0.506051000000	-2.776927000000	-0.050924000000
	H	1.450093000000	-2.748563000000	1.444911000000
	H	-0.135360000000	-1.964315000000	1.368320000000
**7**	C	2.559156000000	-0.335847000000	0.131884000000
	C	2.225482000000	0.911245000000	-0.415718000000
	C	3.160964000000	1.872219000000	-0.761751000000
	C	4.493367000000	1.537511000000	-0.528707000000
	C	4.892454000000	0.305778000000	0.022230000000
	C	3.912138000000	-0.632888000000	0.352515000000
	C	1.306921000000	-1.018116000000	0.333353000000
	H	2.872361000000	2.824355000000	-1.186957000000
	H	5.265099000000	2.256368000000	-0.781702000000
	H	1.145028000000	-1.985008000000	0.781376000000
	C	0.319593000000	-0.175422000000	-0.092286000000
	O	0.868126000000	1.009666000000	-0.549666000000
	C	-1.143390000000	-0.237488000000	-0.085850000000
	C	-1.855413000000	-1.442142000000	-0.234545000000

	C	-1.833243000000	0.973502000000	0.096898000000
	C	-3.259425000000	-1.383831000000	-0.130347000000
	C	-3.220735000000	0.988300000000	0.166286000000
	C	-3.967056000000	-0.197857000000	0.073656000000
	H	-1.260767000000	1.883034000000	0.193020000000
	O	4.324094000000	-1.828104000000	0.886902000000
	H	3.561044000000	-2.383431000000	1.073518000000
	O	-4.020563000000	-2.522920000000	-0.243407000000
	H	-3.449359000000	-3.294619000000	-0.295662000000
	O	-3.957322000000	2.126526000000	0.340593000000
	C	-3.277282000000	3.369180000000	0.458516000000
	H	-2.614817000000	3.381599000000	1.330588000000
	H	-2.697067000000	3.594115000000	-0.442565000000
	H	-4.054713000000	4.121116000000	0.584837000000
	C	6.347134000000	-0.015191000000	0.258902000000
	H	6.542705000000	-0.223212000000	1.315057000000
	H	6.655230000000	-0.904758000000	-0.298584000000
	H	6.981536000000	0.817938000000	-0.048958000000
	C	-1.197573000000	-2.765323000000	-0.550852000000
	H	-1.780882000000	-3.335632000000	-1.283119000000
	H	-0.213548000000	-2.619860000000	-0.993962000000
	H	-1.067763000000	-3.400742000000	0.334939000000
	C	-5.468251000000	-0.175375000000	0.179577000000
	H	-5.858031000000	-1.170171000000	0.388010000000

	H	-5.787483000000	0.511358000000	0.965643000000
	H	-5.922286000000	0.176678000000	-0.753421000000
**8**	C	-2.662048000000	0.256681000000	0.277398000000
	C	-2.343589000000	-0.656025000000	-0.739338000000
	C	-3.292616000000	-1.373136000000	-1.450179000000
	C	-4.620594000000	-1.143353000000	-1.097865000000
	C	-5.003857000000	-0.244911000000	-0.084977000000
	C	-4.010985000000	0.455189000000	0.604633000000
	C	-1.397378000000	0.775406000000	0.740168000000
	H	-3.016068000000	-2.068679000000	-2.231468000000
	H	-5.401930000000	-1.680426000000	-1.624661000000
	H	-1.208283000000	1.491539000000	1.525544000000
	C	-0.428484000000	0.169590000000	0.002935000000
	O	-0.986077000000	-0.710031000000	-0.907190000000
	C	1.043457000000	0.261334000000	0.021140000000
	C	1.680777000000	1.471962000000	-0.316982000000
	C	1.786258000000	-0.876874000000	0.391443000000
	C	3.078511000000	1.522374000000	-0.243443000000
	C	3.183926000000	-0.774403000000	0.412450000000
	C	3.854731000000	0.418422000000	0.121406000000
	O	-4.407763000000	1.327222000000	1.587590000000
	H	-3.636325000000	1.722721000000	2.004846000000
	O	3.773192000000	2.671979000000	-0.538131000000
	H	3.158759000000	3.382126000000	-0.747238000000

	O	3.932206000000	-1.870685000000	0.808115000000
	C	4.299522000000	-2.756569000000	-0.250809000000
	H	3.416107000000	-3.173690000000	-0.745890000000
	H	4.919150000000	-2.245845000000	-0.996342000000
	H	4.873211000000	-3.563557000000	0.205407000000
	C	-6.454766000000	-0.027315000000	0.265407000000
	H	-6.650423000000	-0.261423000000	1.315958000000
	H	-6.749272000000	1.015867000000	0.116029000000
	H	-7.099446000000	-0.655975000000	-0.351445000000
	C	0.932590000000	2.701242000000	-0.780104000000
	H	1.340107000000	3.081191000000	-1.725173000000
	H	-0.120604000000	2.488580000000	-0.951149000000
	H	0.981539000000	3.515742000000	-0.045962000000
	C	5.353077000000	0.543439000000	0.202645000000
	H	5.796151000000	0.654074000000	-0.792662000000
	H	5.632211000000	1.435942000000	0.767447000000
	H	5.786889000000	-0.330422000000	0.685754000000
	C	1.113950000000	-2.165982000000	0.801511000000
	H	0.187830000000	-1.968154000000	1.345082000000
	H	0.849394000000	-2.780205000000	-0.064990000000
	H	1.773659000000	-2.749719000000	1.443219000000
**9**	C	-2.757519000000	-0.379375000000	0.063778000000
	C	-2.497697000000	0.966611000000	-0.229111000000
	C	-3.487482000000	1.921671000000	-0.391498000000

	C	-4.798105000000	1.472181000000	-0.242239000000
	C	-5.124412000000	0.135843000000	0.053487000000
	C	-4.090956000000	-0.791694000000	0.204955000000
	C	-1.469024000000	-1.015879000000	0.143797000000
	H	-3.255410000000	2.953213000000	-0.621197000000
	H	-5.610708000000	2.180961000000	-0.359271000000
	H	-1.258548000000	-2.043318000000	0.385384000000
	C	-0.528971000000	-0.052445000000	-0.098545000000
	O	-1.149322000000	1.165494000000	-0.326163000000
	C	0.933747000000	-0.021878000000	-0.111682000000
	C	1.723324000000	-1.173293000000	-0.301375000000
	C	1.550968000000	1.229536000000	0.077022000000
	C	3.122569000000	-1.015882000000	-0.258674000000
	C	2.932705000000	1.345375000000	0.086654000000
	C	3.754893000000	0.216943000000	-0.083479000000
	H	0.922761000000	2.095733000000	0.215050000000
	O	-4.432481000000	-2.089468000000	0.491541000000
	H	-3.639899000000	-2.632114000000	0.545788000000
	O	3.918630000000	-2.131764000000	-0.466684000000
	O	3.595890000000	2.528954000000	0.260871000000
	C	4.259758000000	-2.850033000000	0.719568000000
	H	4.821234000000	-2.218875000000	1.417062000000
	H	3.365887000000	-3.231716000000	1.225409000000
	H	4.883266000000	-3.687826000000	0.406692000000

	C	2.835841000000	3.720671000000	0.412853000000
	H	2.211723000000	3.911476000000	-0.466526000000
	H	2.203222000000	3.680450000000	1.306027000000
	H	3.562957000000	4.523922000000	0.521761000000
	C	-6.557819000000	-0.308219000000	0.206220000000
	H	-6.811514000000	-1.091368000000	-0.514388000000
	H	-6.742632000000	-0.724759000000	1.200903000000
	H	-7.240159000000	0.530113000000	0.054644000000
	C	1.147674000000	-2.535243000000	-0.601846000000
	H	0.797811000000	-3.047214000000	0.302699000000
	H	0.298095000000	-2.460055000000	-1.283680000000
	H	1.904958000000	-3.166979000000	-1.063925000000
	C	5.252491000000	0.366759000000	-0.093836000000
	H	5.632588000000	0.627584000000	0.899650000000
	H	5.731029000000	-0.553814000000	-0.423462000000
	H	5.549698000000	1.178954000000	-0.761422000000
**10**	C	-3.090622000000	-0.455468000000	0.100402000000
	C	-2.857638000000	0.895664000000	-0.197287000000
	C	-3.858811000000	1.842317000000	-0.357113000000
	C	-5.163324000000	1.378873000000	-0.200068000000
	C	-5.444187000000	0.037664000000	0.099428000000
	C	-4.418485000000	-0.887987000000	0.250438000000
	C	-1.791176000000	-1.068940000000	0.173492000000
	H	-3.635035000000	2.874750000000	-0.590399000000

	H	-5.989202000000	2.071444000000	-0.312819000000
	H	-1.562216000000	-2.092180000000	0.415693000000
	C	-0.870158000000	-0.090033000000	-0.077608000000
	O	-1.514746000000	1.116436000000	-0.303456000000
	C	0.591467000000	-0.032874000000	-0.102369000000
	C	1.399431000000	-1.170556000000	-0.296912000000
	C	1.187732000000	1.229623000000	0.079747000000
	C	2.796018000000	-0.988417000000	-0.265679000000
	C	2.567244000000	1.369804000000	0.078248000000
	C	3.407760000000	0.255653000000	-0.096993000000
	H	0.545639000000	2.084903000000	0.221921000000
	O	-4.748075000000	-2.184392000000	0.541877000000
	H	-3.952246000000	-2.722902000000	0.590814000000
	O	3.609995000000	-2.089937000000	-0.479414000000
	O	3.210866000000	2.564990000000	0.245164000000
	C	3.973073000000	-2.802408000000	0.703974000000
	H	4.527134000000	-2.161058000000	1.398033000000
	H	3.090292000000	-3.201707000000	1.215636000000
	H	4.610387000000	-3.627733000000	0.385936000000
	C	2.431687000000	3.744051000000	0.399582000000
	H	1.797949000000	3.921574000000	-0.475679000000
	H	1.806657000000	3.695713000000	1.297661000000
	H	3.145837000000	4.559821000000	0.500729000000
	C	0.845481000000	-2.542759000000	-0.591219000000

	H	0.511819000000	-3.059643000000	0.316625000000
	H	-0.010377000000	-2.483647000000	-1.266761000000
	H	1.610151000000	-3.161685000000	-1.058438000000
	C	4.902276000000	0.431793000000	-0.119469000000
	H	5.285965000000	0.696339000000	0.871654000000
	H	5.394086000000	-0.479316000000	-0.455774000000
	H	5.179436000000	1.251030000000	-0.787000000000
	H	-6.467192000000	-0.298492000000	0.215224000000
**11**	C	3.204516000000	0.455362000000	-0.591398000000
	C	2.979821000000	-0.412325000000	0.490923000000
	C	3.995312000000	-1.052369000000	1.186215000000
	C	5.296707000000	-0.800031000000	0.758506000000
	C	5.555022000000	0.060075000000	-0.323017000000
	C	4.522487000000	0.693001000000	-1.005551000000
	C	1.895257000000	0.890954000000	-1.006253000000
	H	3.781050000000	-1.712854000000	2.017189000000
	H	6.125321000000	-1.276878000000	1.269094000000
	H	1.649688000000	1.553302000000	-1.821627000000
	C	0.995381000000	0.287587000000	-0.184921000000
	O	1.640190000000	-0.516704000000	0.741803000000
	C	-0.477816000000	0.311352000000	-0.123380000000
	C	-1.154817000000	1.514644000000	0.159681000000
	C	-1.183357000000	-0.883973000000	-0.364971000000
	C	-2.555215000000	1.495812000000	0.165425000000

	C	-2.583300000000	-0.846777000000	-0.310123000000
	C	-3.295181000000	0.333080000000	-0.068246000000
	H	6.581082000000	0.231024000000	-0.628089000000
	H	4.733275000000	1.354456000000	-1.838221000000
	O	-3.288942000000	2.632107000000	0.413781000000
	H	-2.700332000000	3.384904000000	0.525118000000
	O	-3.297102000000	-2.003266000000	-0.578527000000
	C	-3.560424000000	-2.820142000000	0.563359000000
	H	-2.631219000000	-3.157668000000	1.034831000000
	H	-4.161612000000	-2.281493000000	1.304357000000
	H	-4.118319000000	-3.684937000000	0.203627000000
	C	-0.444588000000	2.809391000000	0.483050000000
	H	-0.820590000000	3.245473000000	1.416883000000
	H	0.625395000000	2.658741000000	0.609989000000
	H	-0.571215000000	3.558733000000	-0.308847000000
	C	-4.799992000000	0.384140000000	-0.066846000000
	H	-5.188483000000	0.575342000000	0.938891000000
	H	-5.155581000000	1.202572000000	-0.697394000000
	H	-5.217983000000	-0.551405000000	-0.434484000000
	C	-0.473514000000	-2.168745000000	-0.722551000000
	H	-0.126895000000	-2.703880000000	0.167095000000
	H	-1.141356000000	-2.827988000000	-1.276735000000
	H	0.406815000000	-1.970757000000	-1.337546000000
	C	-3.123028000000	-0.449894000000	-0.123543000000

**12**	C	-2.860743000000	0.857611000000	0.311251000000
	C	-3.841645000000	1.787045000000	0.623504000000
	C	-5.157961000000	1.353058000000	0.478757000000
	C	-5.468162000000	0.055946000000	0.045194000000
	C	-4.461393000000	-0.853510000000	-0.257860000000
	C	-1.835984000000	-1.060662000000	-0.327511000000
	H	-3.594950000000	2.785545000000	0.959147000000
	H	-5.969208000000	2.034247000000	0.707447000000
	H	-1.633237000000	-2.051525000000	-0.694298000000
	C	-0.891500000000	-0.122447000000	-0.013709000000
	O	-1.511212000000	1.055832000000	0.377222000000
	C	0.569068000000	-0.072491000000	-0.048565000000
	C	1.380247000000	-1.227675000000	-0.031880000000
	C	1.163748000000	1.205370000000	-0.099667000000
	C	2.777671000000	-1.067866000000	-0.095849000000
	C	2.537086000000	1.336365000000	-0.165417000000
	C	3.357700000000	0.199588000000	-0.179603000000
	H	0.526640000000	2.076496000000	-0.098968000000
	H	-6.499296000000	-0.258526000000	-0.058956000000
	O	3.571237000000	-2.187816000000	-0.148661000000
	O	3.232789000000	2.518435000000	-0.243515000000
	C	2.497903000000	3.738152000000	-0.268950000000
	H	1.834154000000	3.778662000000	-1.138163000000
	H	1.912370000000	3.862732000000	0.647135000000

	H	3.238756000000	4.532655000000	-0.338438000000
	C	4.480667000000	-2.381245000000	0.944236000000
	H	5.221986000000	-1.582301000000	0.989954000000
	H	3.933681000000	-2.437046000000	1.891929000000
	H	4.976241000000	-3.332859000000	0.754387000000
	C	0.823203000000	-2.625494000000	0.064054000000
	H	1.604321000000	-3.326153000000	0.351475000000
	H	0.011435000000	-2.678358000000	0.792743000000
	H	0.429791000000	-2.967369000000	-0.900119000000
	O	-4.819784000000	-2.106874000000	-0.676190000000
	H	-4.033233000000	-2.637504000000	-0.836098000000
	O	4.709167000000	0.331886000000	-0.285697000000
	H	4.901714000000	1.278486000000	-0.341087000000
**13**	C	2.814218000000	-0.665477000000	0.345289000000
	C	2.590653000000	0.572402000000	-0.279576000000
	C	3.605240000000	1.445709000000	-0.641525000000
	C	4.905860000000	1.042674000000	-0.347004000000
	C	5.163501000000	-0.187190000000	0.283716000000
	C	4.131496000000	-1.049439000000	0.634860000000
	C	1.508670000000	-1.238466000000	0.532835000000
	H	3.391851000000	2.390164000000	-1.126368000000
	H	5.734457000000	1.690386000000	-0.608677000000
	H	1.277349000000	-2.170197000000	1.021292000000
	C	0.602684000000	-0.349991000000	0.029403000000

	O	1.252519000000	0.768106000000	-0.469375000000
	C	-0.861357000000	-0.302783000000	-0.015499000000
	C	-1.648430000000	-1.465863000000	-0.131728000000
	C	-1.459236000000	0.967380000000	0.085465000000
	C	-3.047286000000	-1.300497000000	-0.085687000000
	C	-2.845490000000	1.086777000000	0.102446000000
	C	-3.644223000000	-0.053827000000	0.027744000000
	H	-0.820397000000	1.833396000000	0.162950000000
	H	-4.722605000000	0.031039000000	0.055978000000
	H	6.188933000000	-0.465675000000	0.498040000000
	H	4.341572000000	-1.995725000000	1.120244000000
	O	-3.904472000000	-2.366996000000	-0.162884000000
	O	-3.514998000000	2.271148000000	0.203195000000
	C	-2.760318000000	3.473585000000	0.281914000000
	H	-2.123846000000	3.488664000000	1.173138000000
	H	-2.141759000000	3.616277000000	-0.610446000000
	H	-3.491304000000	4.277849000000	0.347160000000
	H	-3.406376000000	-3.189485000000	-0.190886000000
	C	-1.076506000000	-2.847073000000	-0.348573000000
	H	-0.079392000000	-2.798600000000	-0.783917000000
	H	-1.000995000000	-3.427614000000	0.579524000000
	H	-1.688729000000	-3.426074000000	-1.049995000000
	C	-3.128352000000	-0.778876000000	0.185093000000
	C	-3.076598000000	0.592589000000	-0.111834000000

**14**	C	-4.200418000000	1.389547000000	-0.267287000000
	C	-5.433903000000	0.760873000000	-0.109769000000
	C	-5.520866000000	-0.609651000000	0.191122000000
	C	-4.380564000000	-1.390406000000	0.341422000000
	C	-1.757558000000	-1.206452000000	0.253767000000
	H	-4.117541000000	2.444118000000	-0.498718000000
	H	-6.343078000000	1.340168000000	-0.221144000000
	H	-1.407862000000	-2.194838000000	0.494924000000
	C	-0.979141000000	-0.111099000000	0.001676000000
	O	-1.778470000000	0.999301000000	-0.222579000000
	C	0.460994000000	0.142301000000	-0.031009000000
	C	1.409330000000	-0.885939000000	-0.251425000000
	C	0.888207000000	1.469772000000	0.155627000000
	C	2.761937000000	-0.525333000000	-0.221598000000
	C	2.231229000000	1.803580000000	0.134986000000
	C	3.175898000000	0.794472000000	-0.052811000000
	H	0.162843000000	2.256143000000	0.310982000000
	H	-6.498477000000	-1.063441000000	0.307267000000
	H	-4.459308000000	-2.446461000000	0.573514000000
	O	4.498159000000	1.146429000000	-0.072270000000
	H	4.997589000000	0.372363000000	-0.369486000000
	O	2.618967000000	3.100599000000	0.303933000000
	H	3.580756000000	3.145437000000	0.228287000000
	O	3.787024000000	-1.436155000000	-0.446192000000

	C	4.112941000000	-2.271837000000	0.675928000000
	H	3.268887000000	-2.908301000000	0.952645000000
	H	4.949210000000	-2.895201000000	0.360284000000
	H	4.406476000000	-1.666830000000	1.539861000000
	C	1.040196000000	-2.310039000000	-0.585451000000
	H	0.790130000000	-2.898837000000	0.304464000000
	H	0.173649000000	-2.341412000000	-1.248341000000
	H	1.871293000000	-2.805320000000	-1.088305000000
**15**	C	-2.968766000000	-0.456930000000	0.640138000000
	C	-2.817247000000	0.311216000000	-0.526766000000
	C	-3.882350000000	0.795287000000	-1.272113000000
	C	-5.158365000000	0.485040000000	-0.807490000000
	C	-5.344209000000	-0.279294000000	0.357565000000
	C	-4.262556000000	-0.755559000000	1.089628000000
	C	-1.627797000000	-0.744028000000	1.082831000000
	H	-3.723611000000	1.381995000000	-2.168380000000
	H	-6.023702000000	0.839987000000	-1.355129000000
	H	-1.327657000000	-1.307273000000	1.952447000000
	C	-0.781532000000	-0.159142000000	0.195439000000
	O	-1.490658000000	0.496049000000	-0.800976000000
	C	0.689789000000	-0.086275000000	0.109895000000
	C	1.315437000000	1.173007000000	0.228691000000
	C	1.435765000000	-1.261092000000	-0.089491000000
	C	2.709485000000	1.229660000000	0.128456000000

	C	2.830864000000	-1.140216000000	-0.152686000000
	C	3.493312000000	0.085366000000	-0.055356000000
	H	-6.352505000000	-0.500736000000	0.688345000000
	H	-4.417444000000	-1.345282000000	1.986013000000
	O	3.385672000000	2.424113000000	0.219676000000
	H	2.756489000000	3.145165000000	0.317970000000
	O	3.534378000000	-2.306443000000	-0.332549000000
	H	4.474228000000	-2.112162000000	-0.404629000000
	C	0.540030000000	2.444774000000	0.488267000000
	H	0.465239000000	3.075553000000	-0.405728000000
	H	-0.477137000000	2.233534000000	0.813200000000
	H	1.002512000000	3.040901000000	1.285071000000
	C	0.825660000000	-2.630633000000	-0.264151000000
	H	0.978337000000	-3.251073000000	0.625680000000
	H	-0.244599000000	-2.572225000000	-0.453908000000
	H	1.302425000000	-3.156174000000	-1.094411000000
	C	4.997261000000	0.164242000000	-0.146660000000
	H	5.482254000000	-0.402894000000	0.657412000000
	H	5.366318000000	-0.226754000000	-1.103240000000
	H	5.335015000000	1.195153000000	-0.067098000000
	C	2.110563000000	0.162041000000	-0.000461000000
	C	1.879574000000	-1.230428000000	-0.000278000000
	C	2.871859000000	-2.192008000000	-0.000156000000
	C	4.180138000000	-1.715957000000	-0.000104000000

**16**	C	4.489208000000	-0.348249000000	-0.000153000000
	C	3.452810000000	0.600064000000	-0.000350000000
	C	0.790322000000	0.750891000000	-0.000348000000
	H	2.639388000000	-3.248683000000	-0.000106000000
	H	4.999416000000	-2.426569000000	-0.000009000000
	H	0.527305000000	1.792960000000	-0.000524000000
	C	-0.110558000000	-0.270960000000	-0.000229000000
	O	0.536684000000	-1.487336000000	-0.000260000000
	C	-1.568829000000	-0.303726000000	-0.000099000000
	C	-2.242874000000	-1.537154000000	0.000338000000
	C	-2.301709000000	0.884947000000	-0.000177000000
	C	-3.632252000000	-1.555217000000	0.000482000000
	C	-3.698271000000	0.845741000000	0.000021000000
	C	-4.376963000000	-0.373385000000	0.000309000000
	H	-1.817325000000	1.852159000000	-0.000471000000
	H	-1.678339000000	-2.461573000000	0.000619000000
	H	-5.455164000000	-0.441712000000	0.000415000000
	O	-4.347040000000	-2.723318000000	0.000967000000
	H	-3.741138000000	-3.471781000000	-0.001409000000
	O	3.875024000000	1.900424000000	-0.000544000000
	O	-4.318909000000	2.060379000000	0.000096000000
	C	2.942912000000	2.972661000000	0.001656000000
	H	2.315092000000	2.959399000000	-0.894133000000
	H	2.316102000000	2.956520000000	0.898113000000

	H	3.542900000000	3.881782000000	0.002716000000
	C	-5.740509000000	2.100951000000	-0.000959000000
	H	-6.153184000000	1.622213000000	-0.895293000000
	H	-6.154538000000	1.623031000000	0.893195000000
	H	-6.006532000000	3.156675000000	-0.001616000000
	C	5.923118000000	0.120282000000	-0.000066000000
	H	6.141657000000	0.736691000000	0.876873000000
	H	6.605264000000	-0.731857000000	0.000023000000
	H	6.141788000000	0.736605000000	-0.877038000000
